# Supplementary material for: Luciferase-induced photoreductive uncaging of small-molecule effectors
Source: Nat Commun. 2018 Aug 30;9:3539. doi: 10.1038/s41467-018-05916-9 (PMC6117273; doi:10.1038/s41467-018-05916-9)
Supplement: Supplementary file 1 — Supplementary Information [file 41467_2018_5916_MOESM1_ESM.pdf]

Luciferase-Induced photoreductive uncaging of small-molecule effectors

Lindberg et al.

Luciferase-Induced photoreductive uncaging of small-molecule effectors

Eric Lindberg[†], Simona Angerani[†], Marcello Anzola[†], Nicolas Winssinger[†*]

[†] Department of Organic Chemistry, NCCR Chemical Biology, Faculty of Science, University of Geneva, 30 quai Ernest-Ansermet, Geneva, Switzerland

Supplementary Information

Supplementary Figures……………………………………………………………………..S3

Supplementary Methods………………………………………………………………………S32

Supplementary References…………………………………………………………………..S48

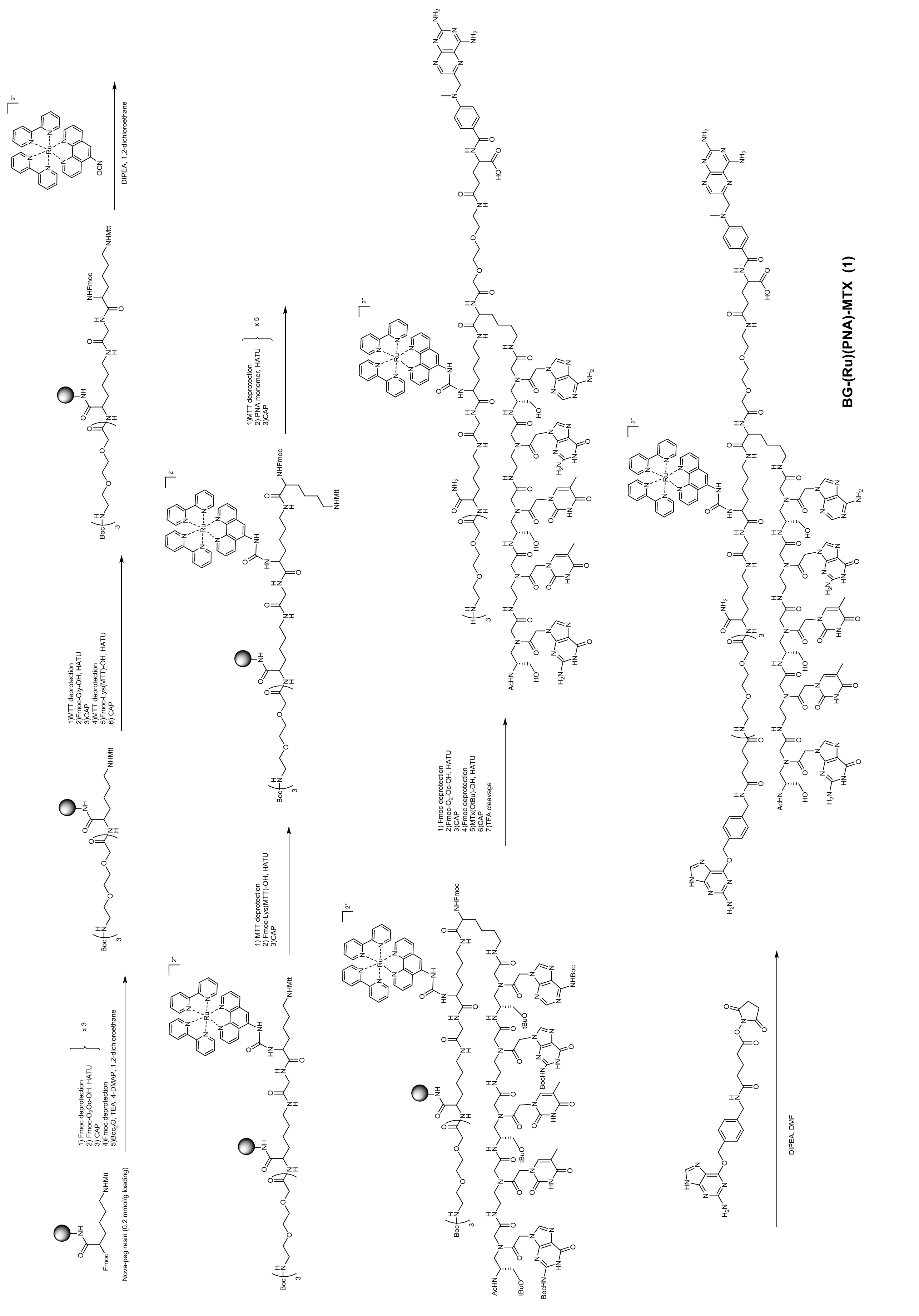

Supplementary Figure 1. Synthetic overview of **1**

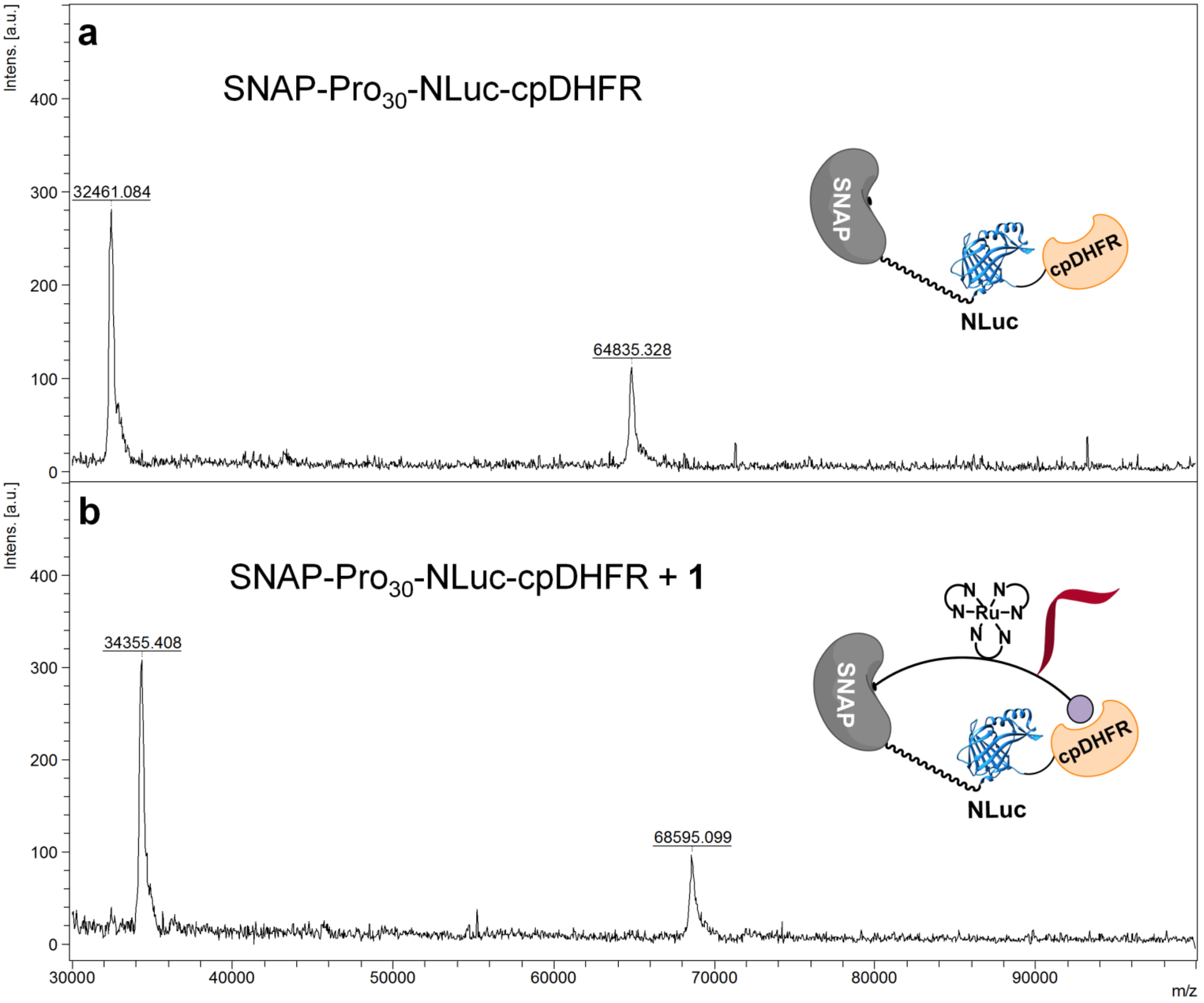

Supplementary Figure 2. MALDI-TOF spectra of SNAP-Pro$_{30}$-NLuc-cpDHFR before (**a**) and after (**b**) labeling with BG-(Ru)(PNA)-MTX (**1**).

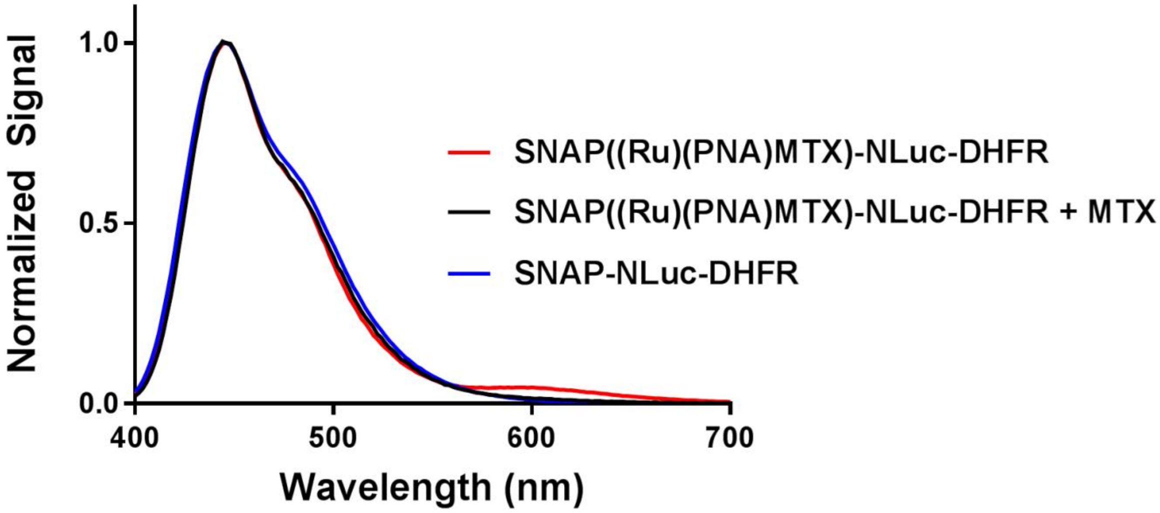

Supplementary Figure 3. Normalized luminescence spectra of SNAP-Pro_{30}-NLuc-cpDHFR labeled with BG-(Ru)(PNA)-MTX (**1**)(red); unlabeled (blue) or labeled with 100 µM methotrexate (black).

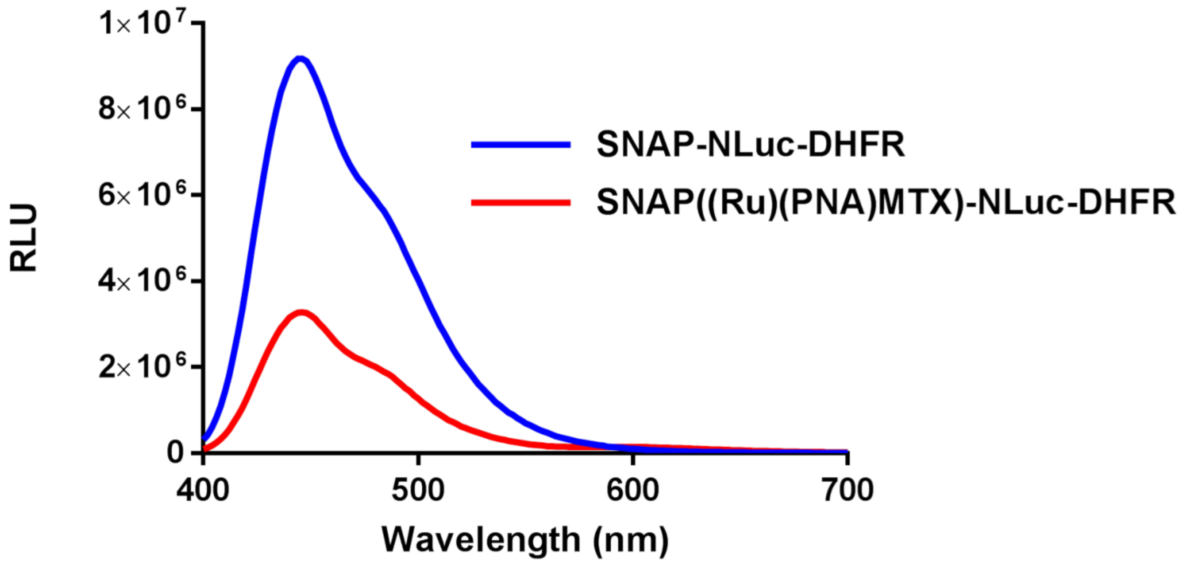

Supplementary Figure 4. Luminescence spectra of SNAP-Pro_{30}-NLuc-cpDHFR with or without labeling with **1**.

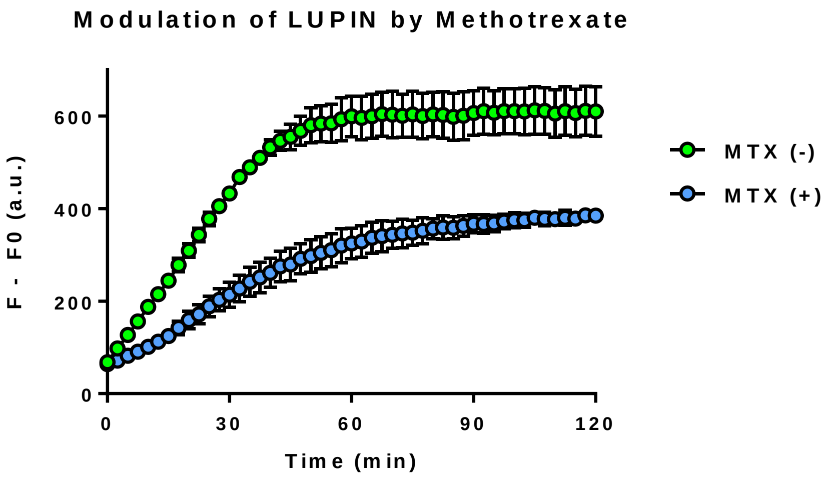

Supplementary Figure 5. Effect of methotrexate concentration on kinetics of LUPIN system. Methotrexate competes with the methotrexate on **1** of the LUPIN sensor, leading to more of the protein construct being in an open conformation, decreasing the BRET efficiency, leading to reduced reaction rate and yield. Conditions: SNAP-Pro$_{30}$-NLuc-cpDHFR labeled with **1** (10 nM), sodium ascorbate (10 mM), PNA-PyRho (**2**)(2.5 µM), and furimazine (100 µM) in HEPES (50 mM) NaCl (50 mM)(pH 7.2).

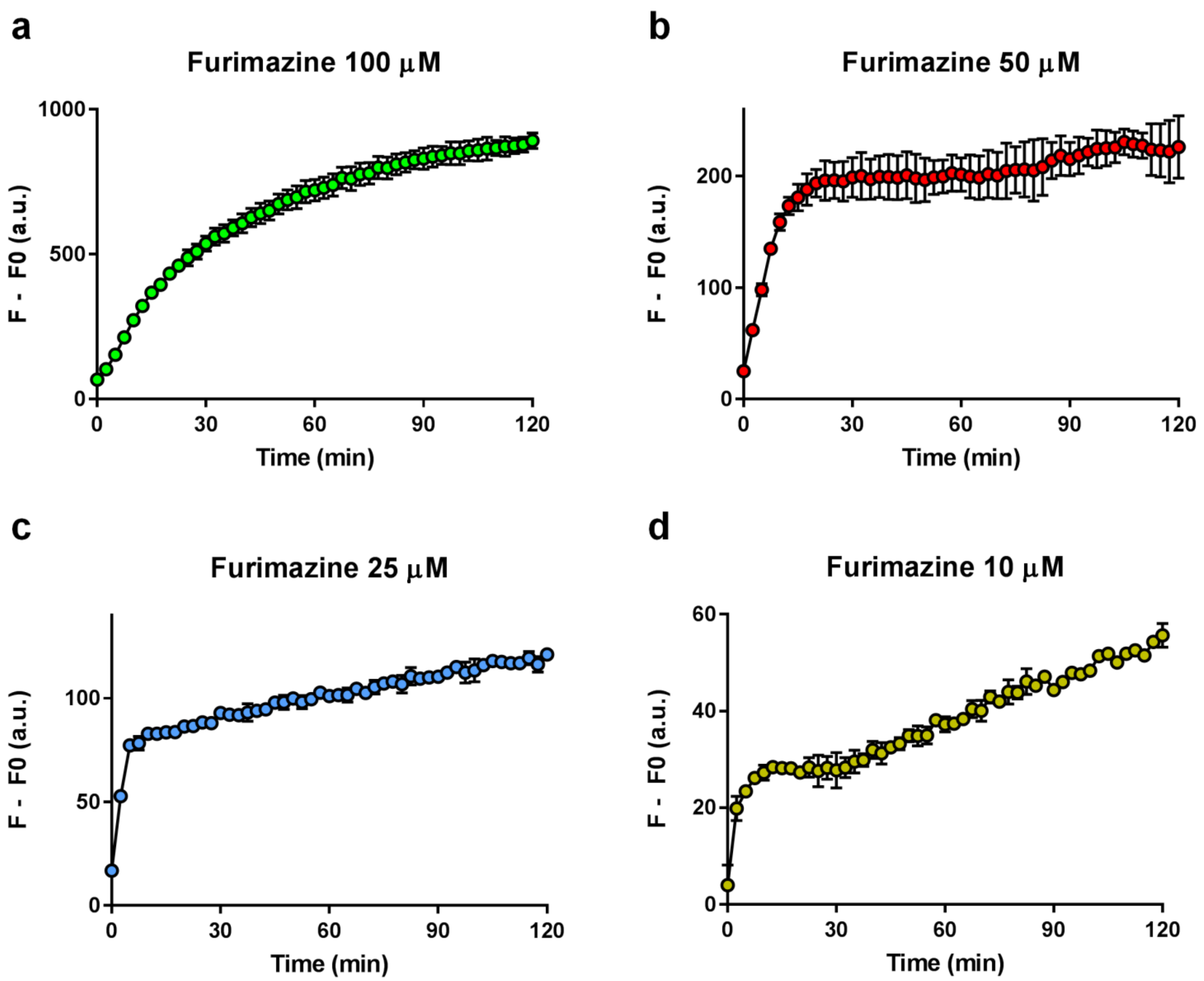

Supplementary Figure 6a-d. Effect of furimazine concentration on kinetics of LUPIN system. Conditions: SNAP-Pro$_{30}$-NLuc-cpDHFR labeled with **1** (10 nM), sodium ascorbate (10 mM), PNA-PyRho (**2**)(5 µM), in Leibovitz's L-15 medium at 37 °C.

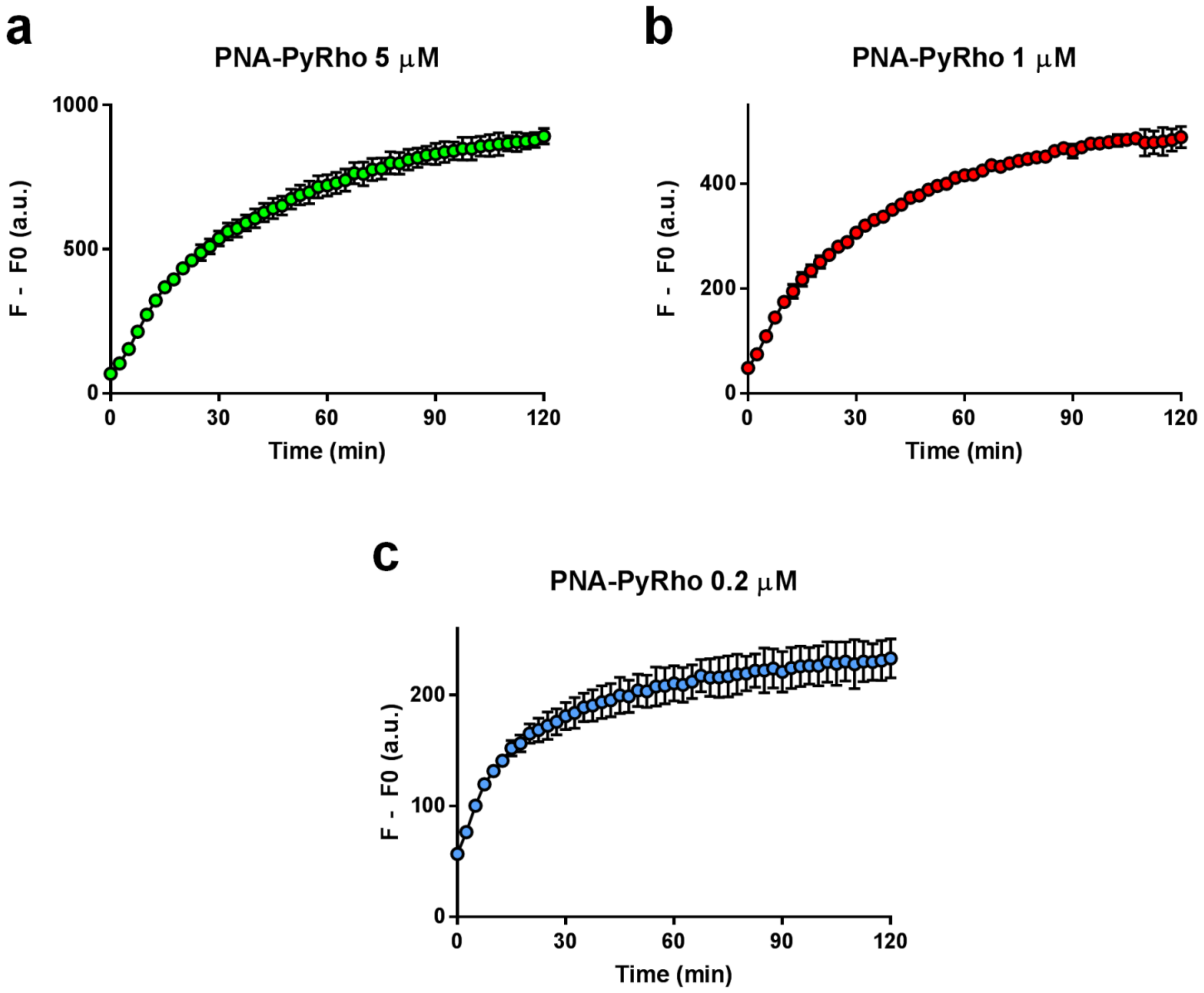

Supplementary Figure 7a-c. Effect of PNA-PyRho (**2**) concentration on kinetics of LUPIN system. Conditions: SNAP-Pro$_{30}$-NLuc-cpDHFR labeled with **1** (10 nM), sodium ascorbate (10 mM), furimazine (100 µM), in Leibovitz's L-15 medium at 37 °C.

.

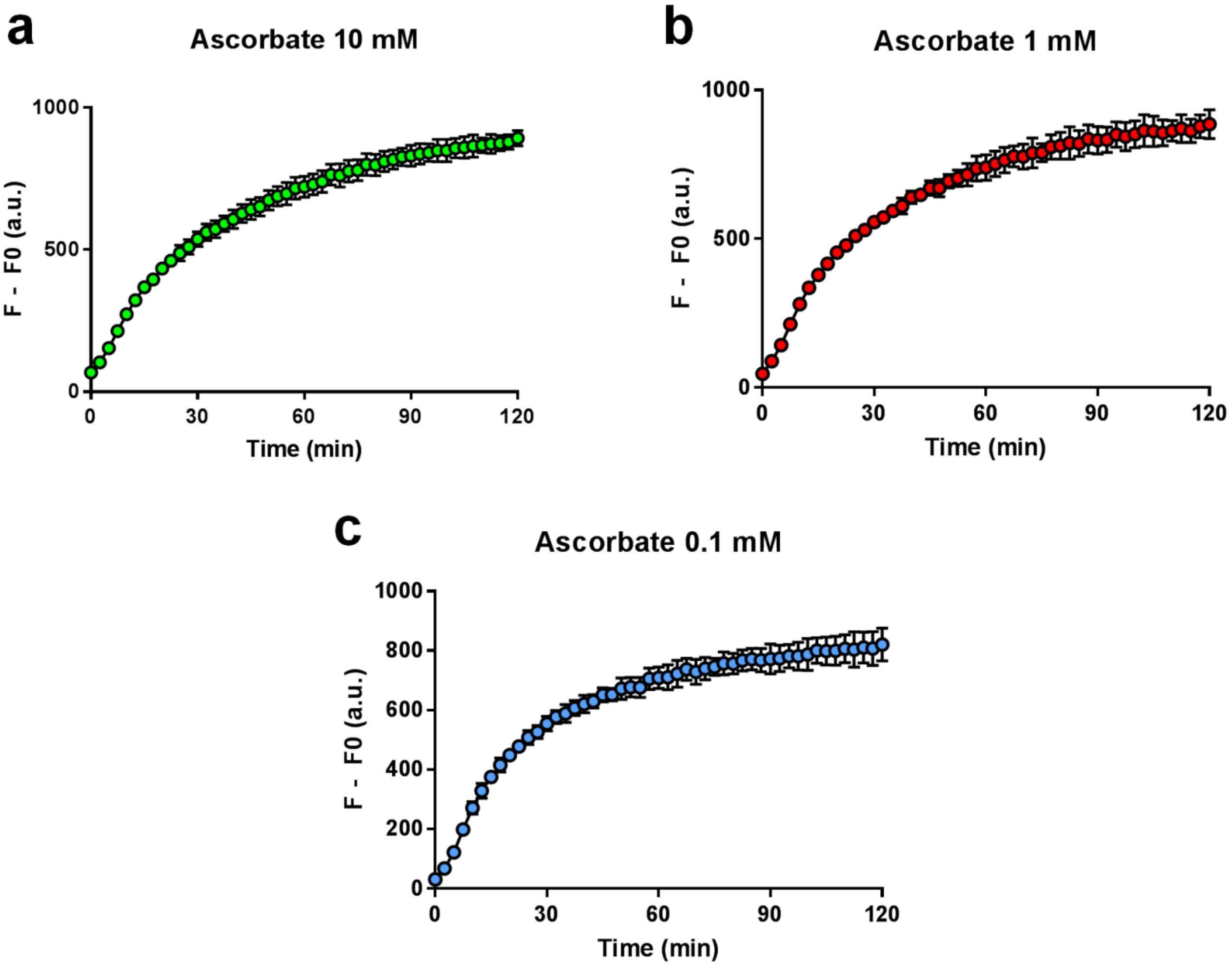

Supplementary Figure 8a-c. Effect of ascorbate concentration on release kinetics of rhodamine with LUPIN system. Conditions: SNAP-Pro$_{30}$-NLuc-cpDHFR labeled with **1** (10 nM), PNA-PyRho (**2**) (5 µM), furimazine (100 µM), in Leibovitz's L-15 medium at 37 °C.

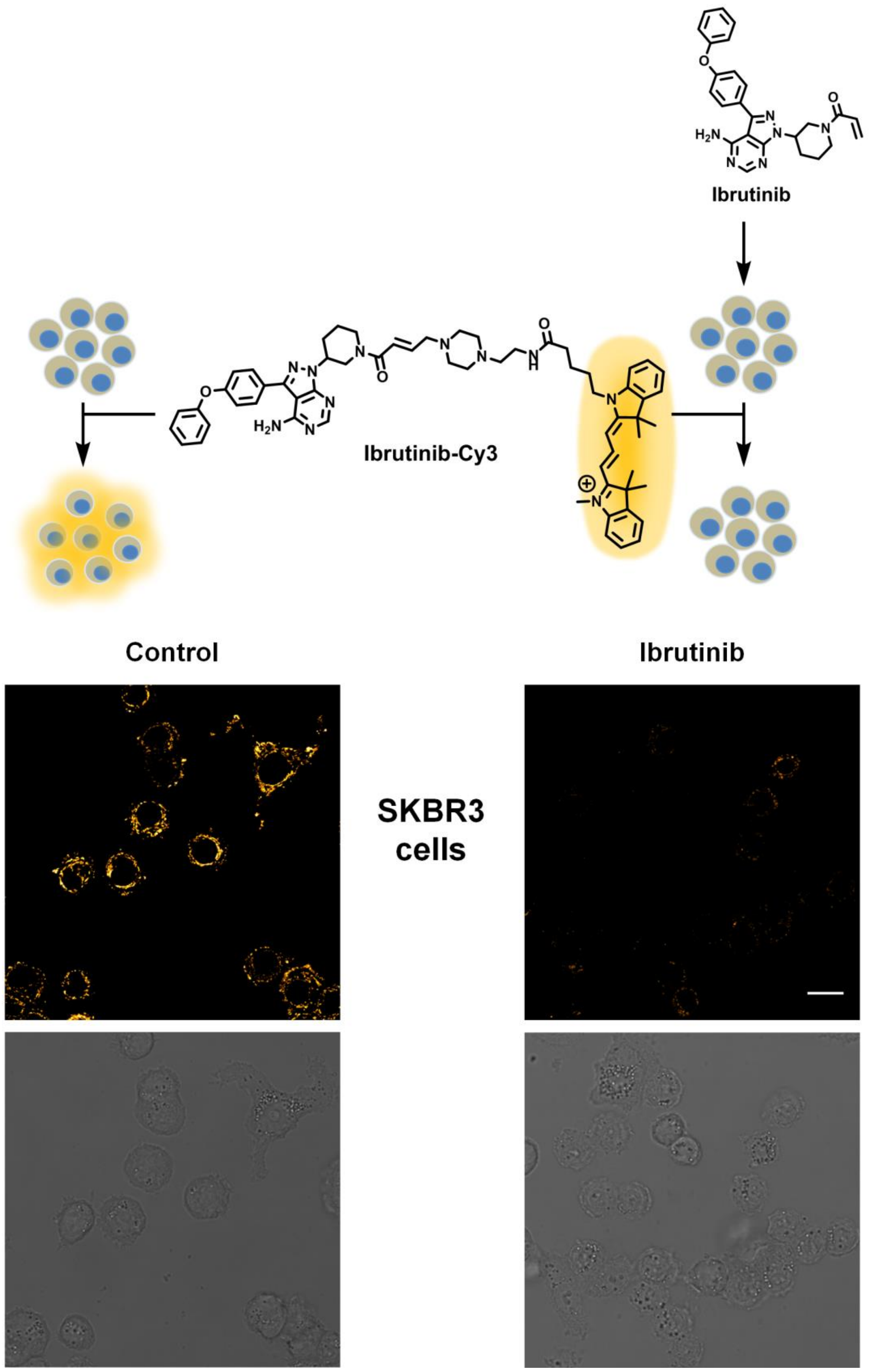

Supplementary Figure 9. Competition of free Ibrutinib versus Ibrutinib-Cy3 in SKBR3 cells. SKBR3 cells were incubated with Ibrutinib (1 µM) for 30 min, followed by Ibrutinib-Cy3 (**9**) (50 nM) for 30 min after which the cells were imaged. In the control experiment the cells were exposed to Ibrutinib-Cy3 (**9**) (50 nM) for 30 mins, followed by imaging. Scale bar: 20 µm.

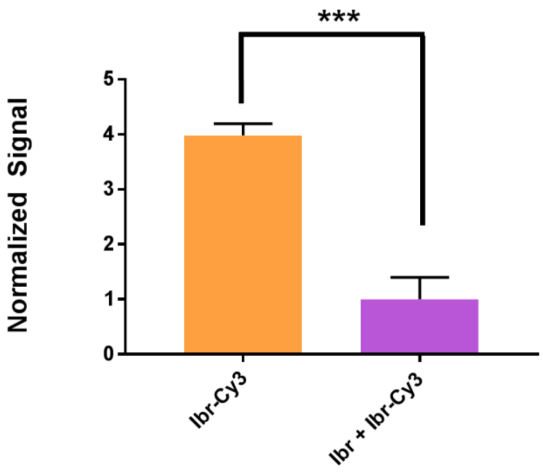

Supplementary Figure 10. Relative quantification of fluorescence in SKBR3 cells. Average fluorescence was measured and normalized to SKBR3 cell populations exposed to Ibrutinib and Ibrutinib-Cy3 (**9**). N = 7. Statistics calculated using a two-tailed *t*-test with unequal variances (Welch´s unpaired *t*-test). *** p<0.001.

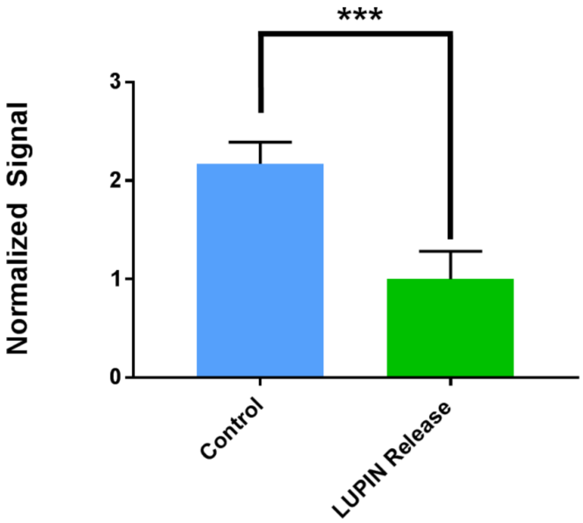

Supplementary Figure 11. Relative quantification of LUPIN release of Ibrutinib in SKBR3 cells. Average fluorescence was measured and normalized to SKBR3 cell populations exposed to LUPIN release of Ibrutinib and Ibrutinib-Cy3 (**9**). Control cell population was exposed to PNA-PyIbr (**7**) and Ibr-Cy3 (**9**). N = 9. Statistics calculated using a two-tailed *t*-test with unequal variances (Welch´s unpaired *t*-test). *** p<0.001.

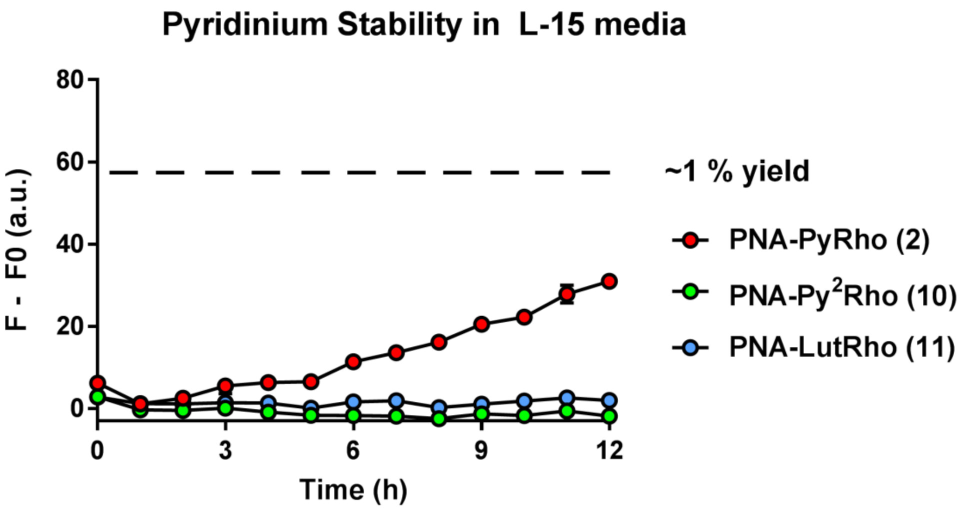

Supplementary Figure 12. Linker stability in Leibovitz's L-15 media. PNA-Rhodamine conjugates with different reductive linkers (**2,10-11**)(1 µM) were incubated in L-15 media at 37°C for 12 h. Release kinetics were compared to internal free rhodamine standards.

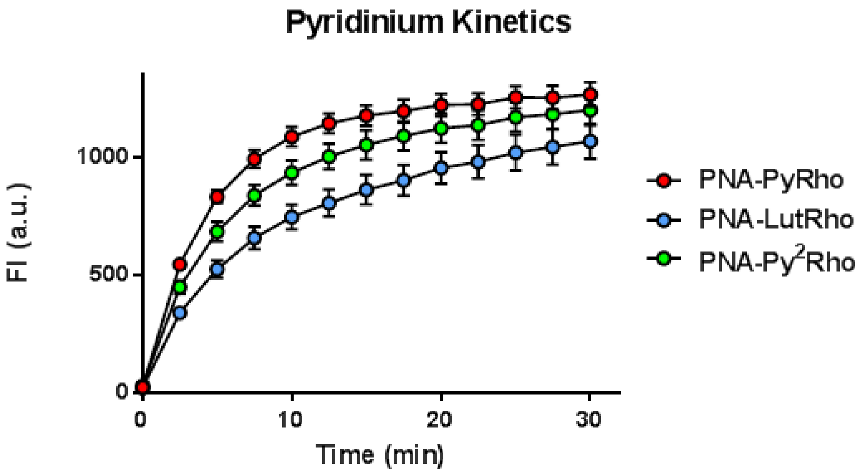

Supplementary Figure 13. Release kinetics of PNA-rhodamine conjugates with 3 different reductive linkers. Conditions: PNA-Linker-Rhodamine (**2, 10-11**) (1 µM), PNA-Ru (**5**) (0.1 µM), sodium ascorbate (10 mM) in PBS 1X (pH 7.4). Irradiation with 1W LED (455 nM) for 30 min.

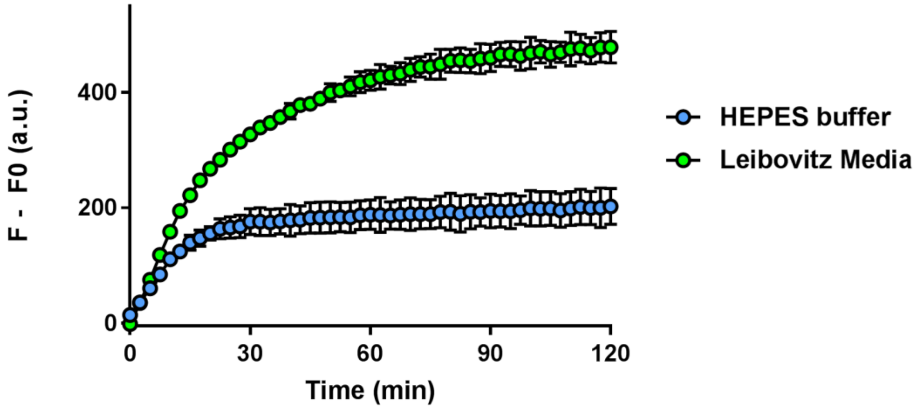

Supplementary Figure 14. Effect of reaction medium on LUPIN reaction rate. Leibovitz's L-15 medium improves reaction rate by 2-fold compared to HEPES buffer. Conditions: SNAP-Pro$_{30}$-NLuc-cpDHFR labeled with **1** (10 nM), PNA-Py^{2}Rho (**10**)(0.2 µM), sodium ascorbate (10 mM) furimazine (100 µM), in HEPES (50 mM) NaCl (50 mM)(pH 7.2)(blue) or Leibovitz's L-15 medium (green) at 37 °C.

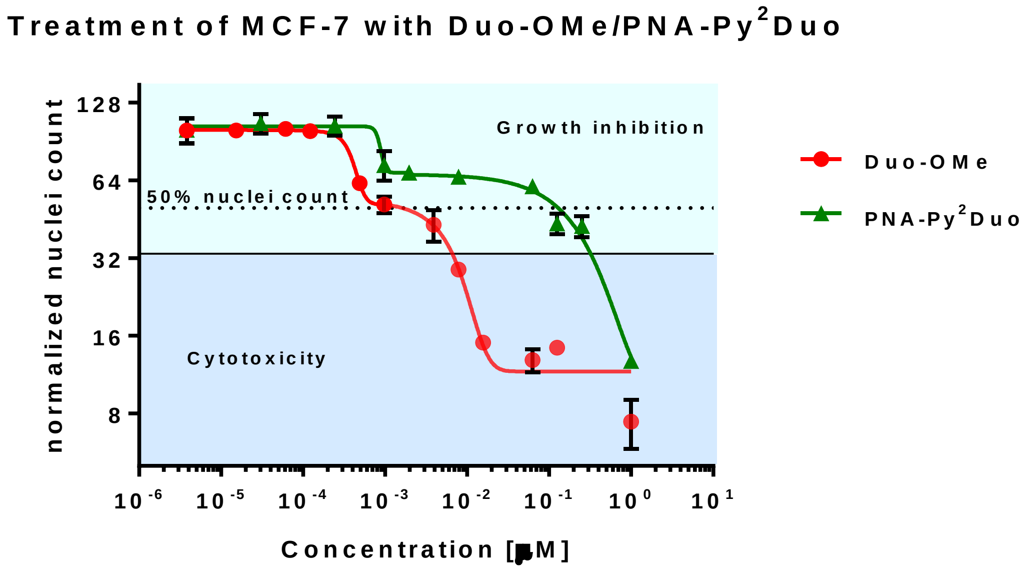

Supplementary Figure 15. Dose response curves for MCF-7 cells after 3 h treatment with Duo-OMe (**4**), PNA-Py^{2}Duo (**12**) and 72 h incubation. Relative toxicity was then evaluated via Hoechst staining, fluorescence imaging and nuclei count for each condition. X and Y-axis logarithmic scale. Cell population doubling time is 29 hours (based on ATCC website); change of background color corresponds to the cell count estimated at the start of the experiment. The experiments were run in triplicates. Error bars indicate standard error of the mean.

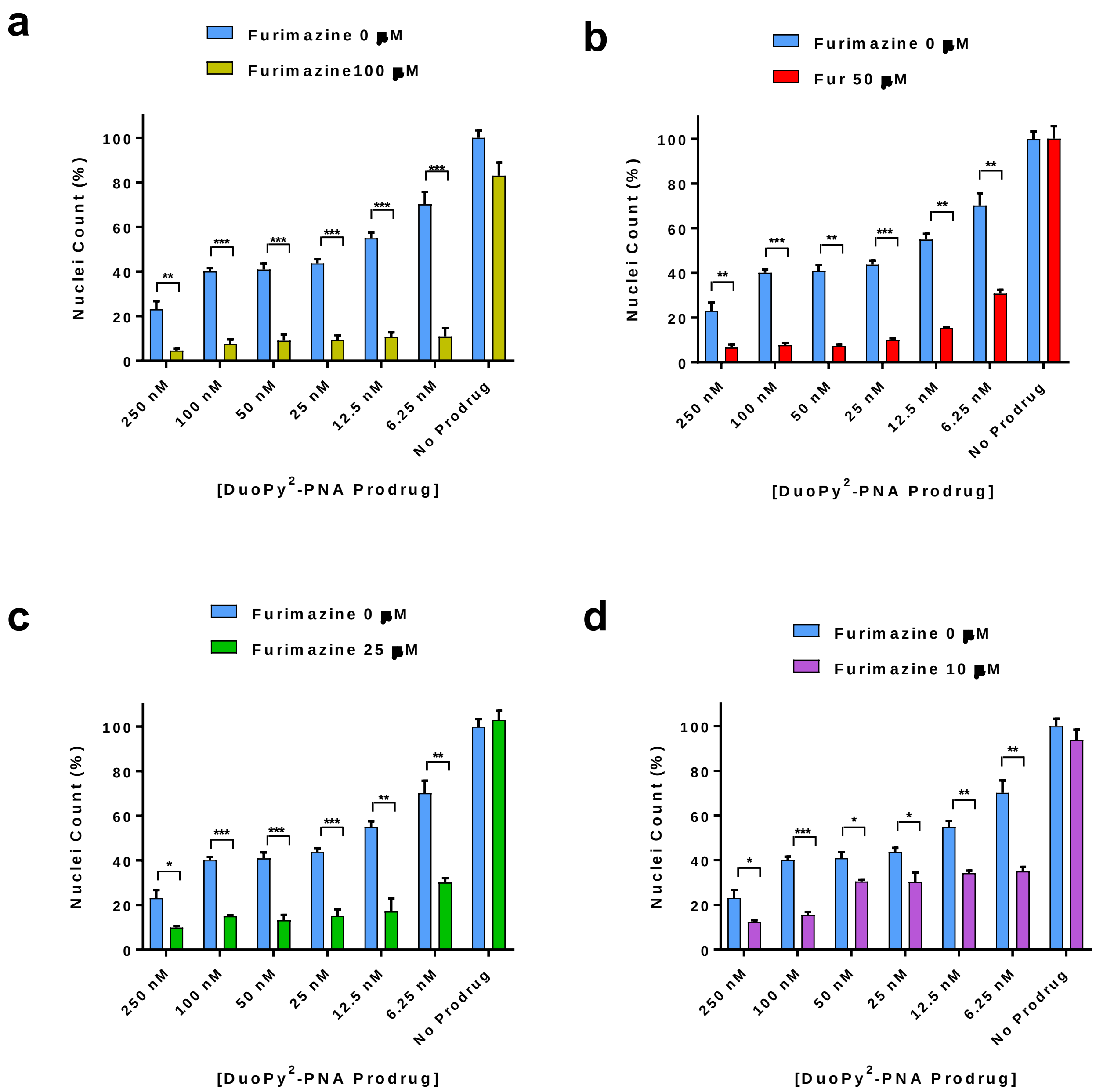

Supplementary Figure 16. Duo-OMe (**4**) release by LUPIN in MCF-7 cell culture. MCF-7 cells were exposed to release reaction for 3 hours after which the media was replaced and the cells were left to grow for 72 hours. Relative toxicity was then evaluated via Hoechst staining, fluorescence imaging and nuclei count for each condition. Conditions: SNAP-Pro$_{30}$-NLuc-cpDHFR labeled with **1** (10 nM), 1 mM Ascorbate, PNA-Py^{2}Duo (**12**) (0-250 nM), and furimazine (**a**, 100 µM; **b**, 50 µM; **c**, 25 µM; **d**, 10 µM) in Leibovitz's medium at 37°C. The data is the average of three independent experiments run in triplicates. Error bars show ± 1 standard deviation from the mean. Statistics calculated using a two-tailed *t*-test with unequal variances (Welch´s unpaired *t*-test). * $p<0.05$; ** $p<0.01$; *** $p<0.001$.

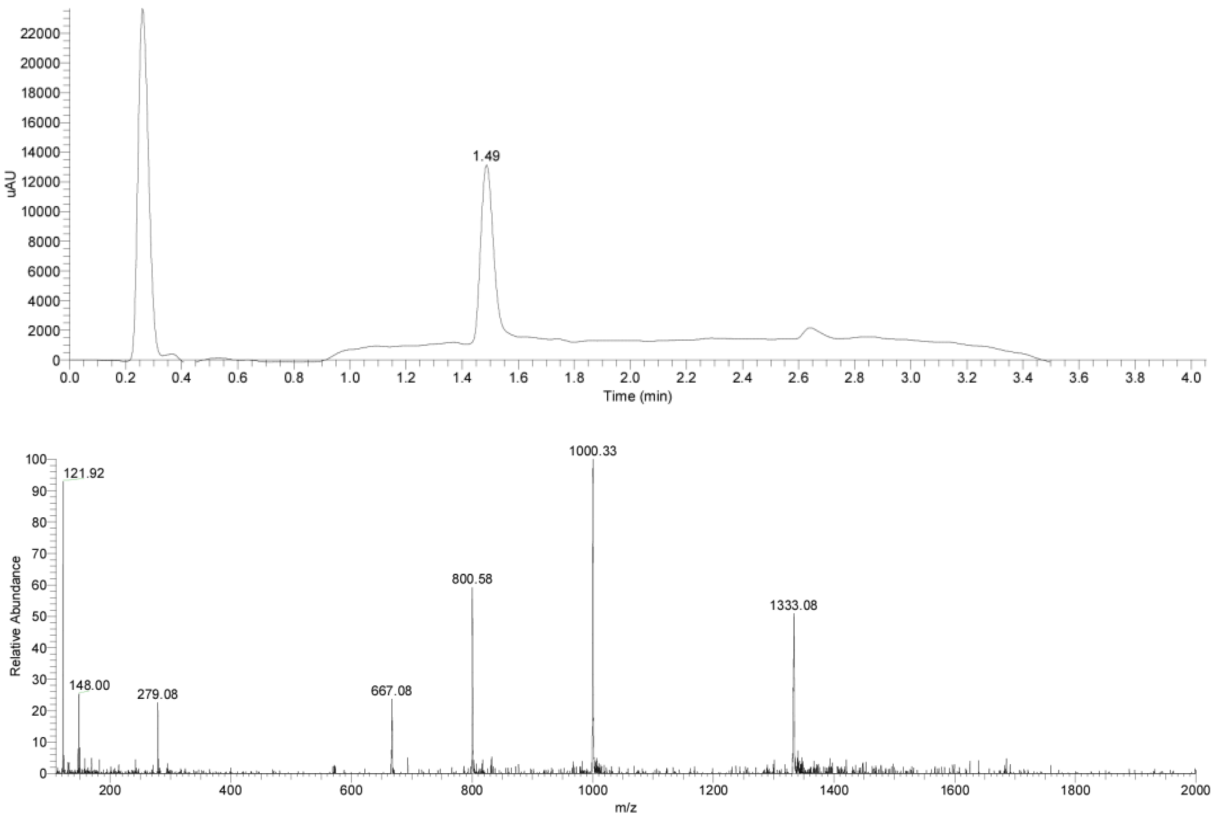

Supplementary Figure 17. LC-MS trace of compound **1**.

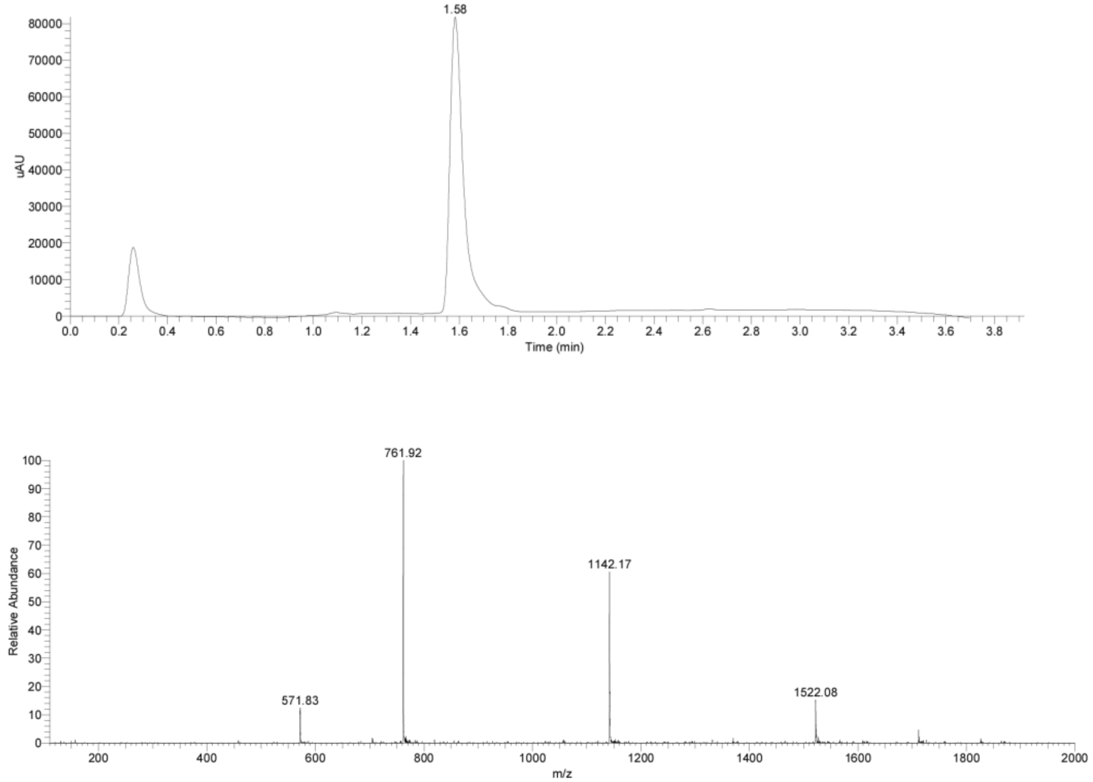

Supplementary Figure 18. LC-MS trace of compound **2**.

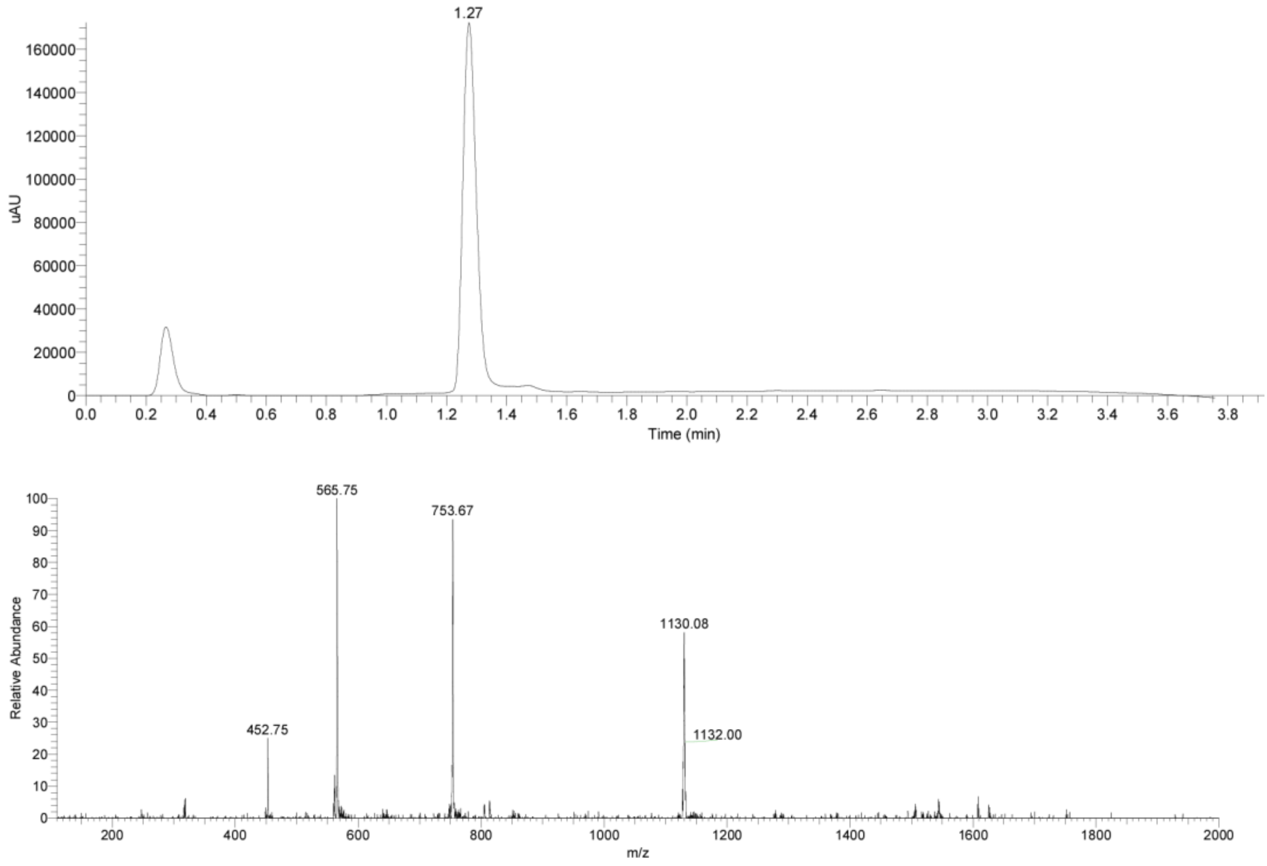

Supplementary Figure 19. LC-MS trace of compound **5**.

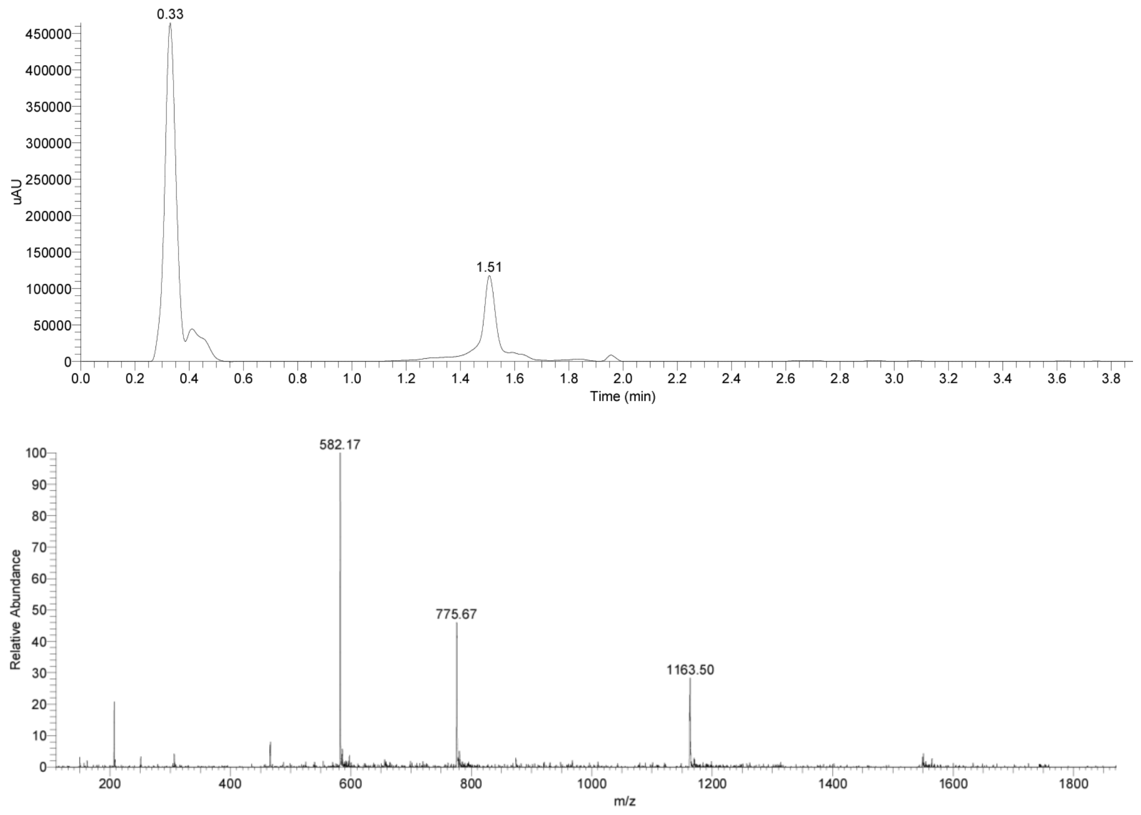

Supplementary Figure 20. LC-MS trace of compound **6**.

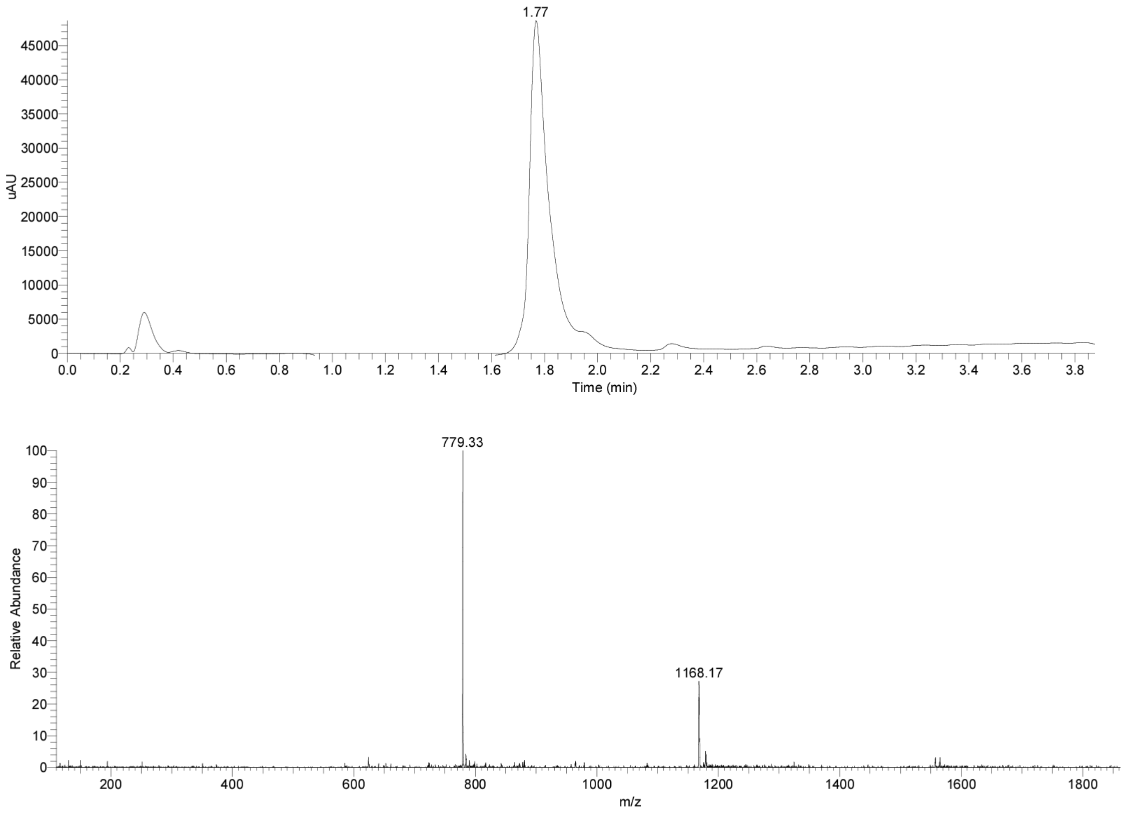

Supplementary Figure 21. LC-MS trace of compound **7**.

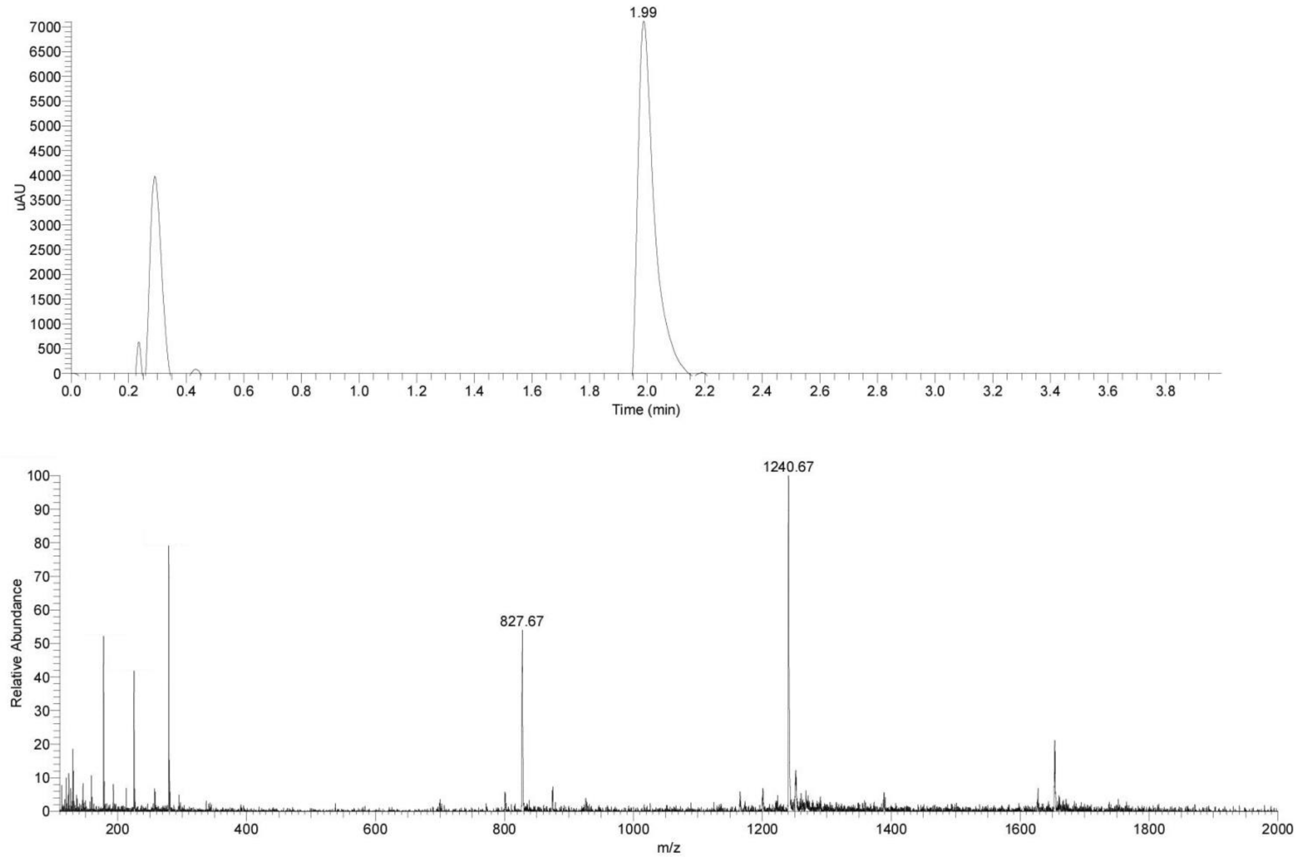

Supplementary Figure 22. LC-MS trace of compound **8**.

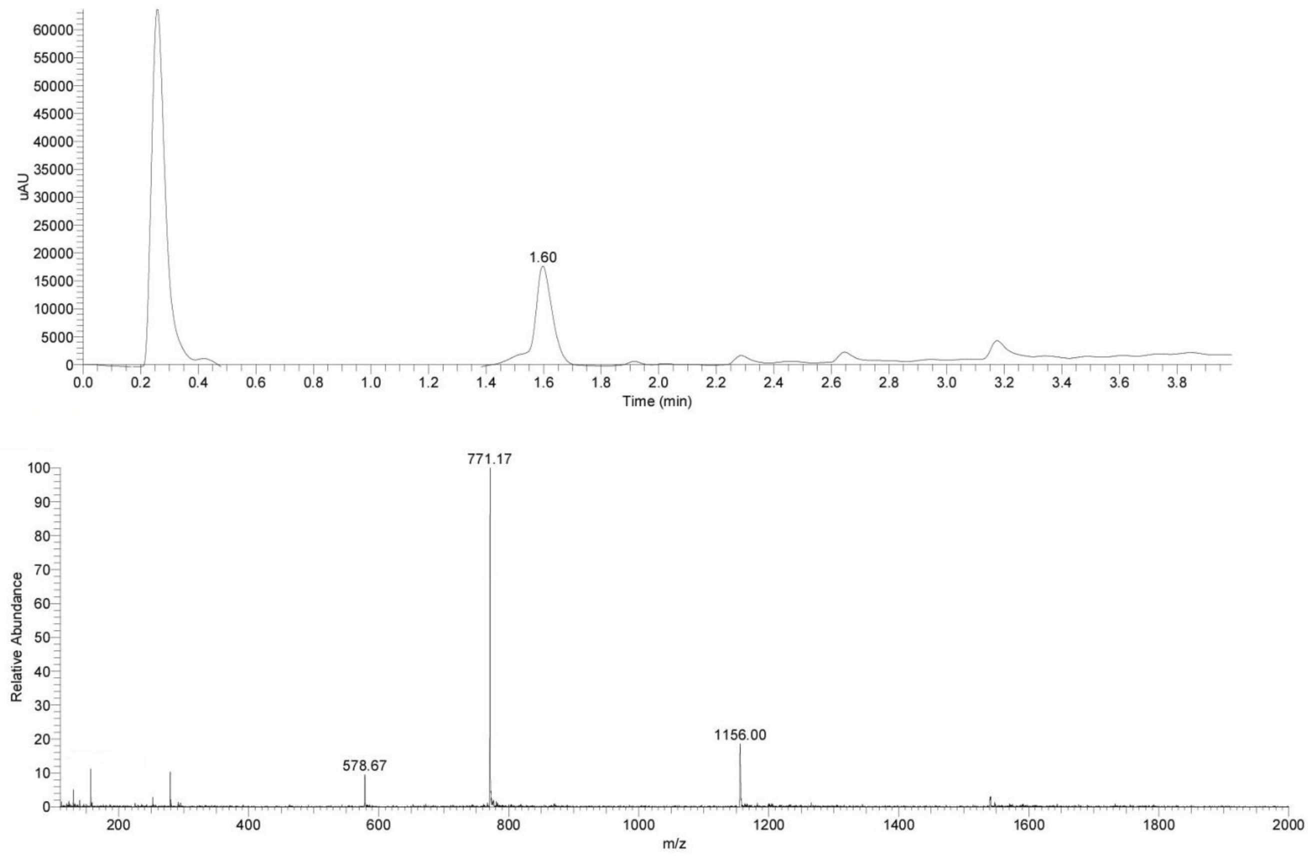

Supplementary Figure 23. LC-MS trace of compound **10**.

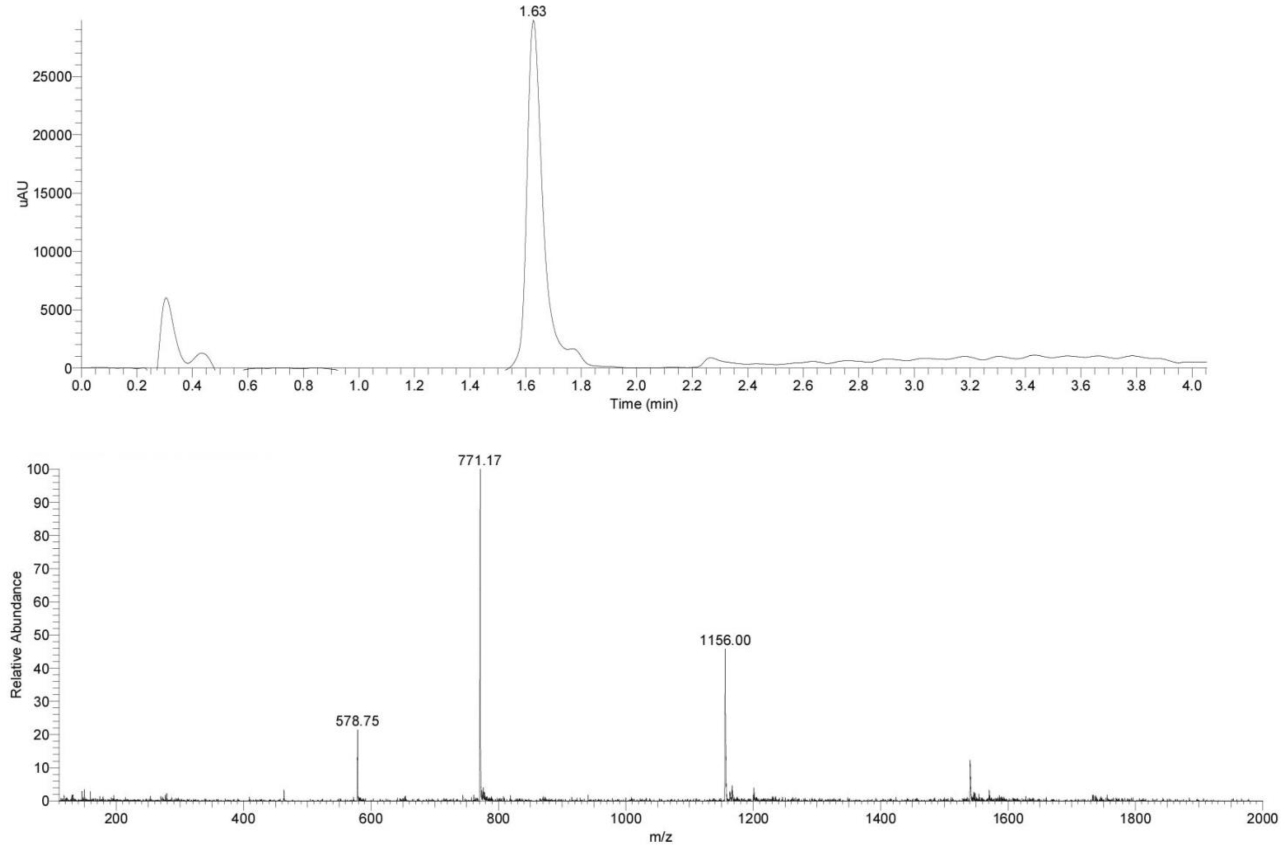

Supplementary Figure 24. LC-MS trace of compound **11**.

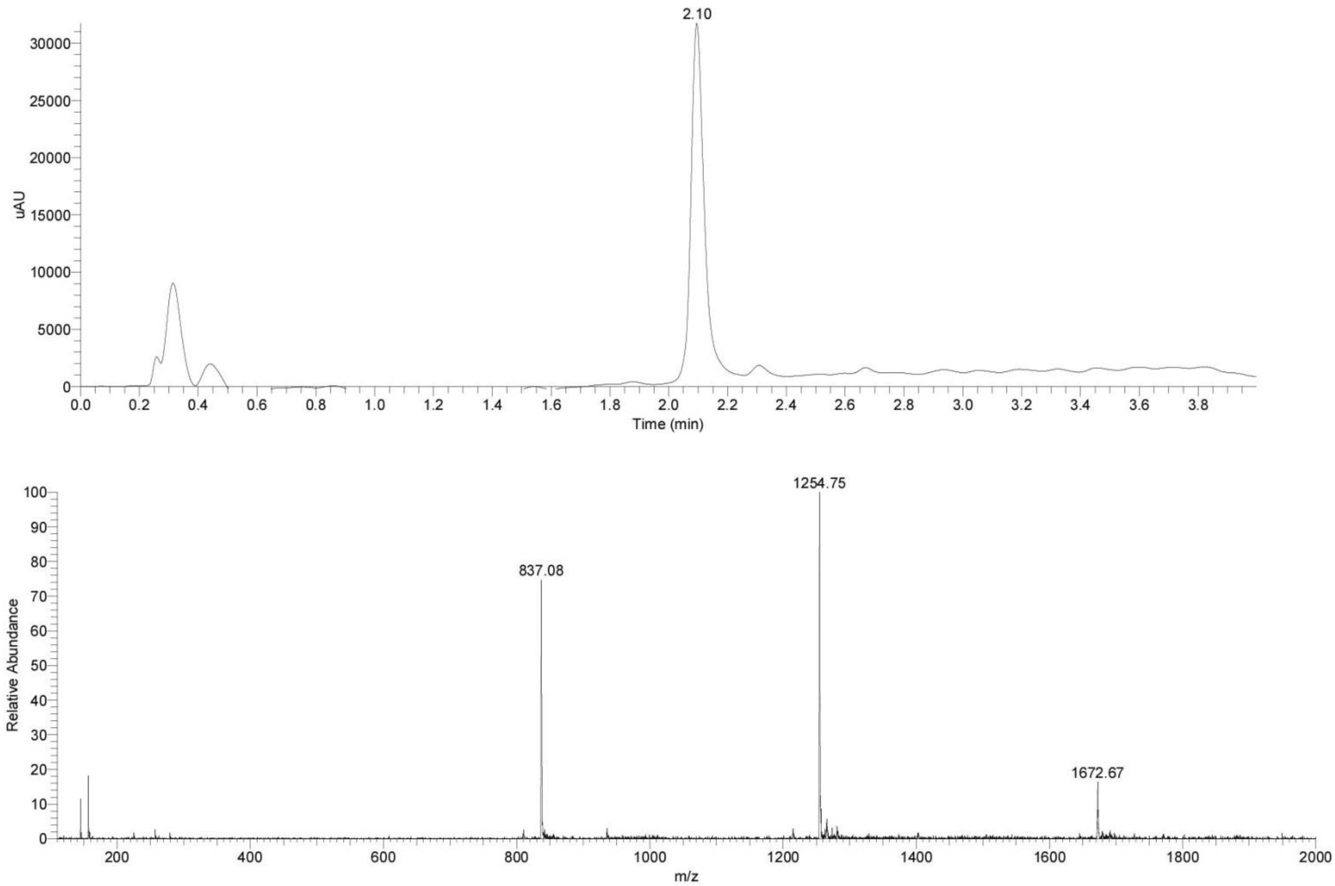

Supplementary Figure 25. LC-MS trace of compound **12**.

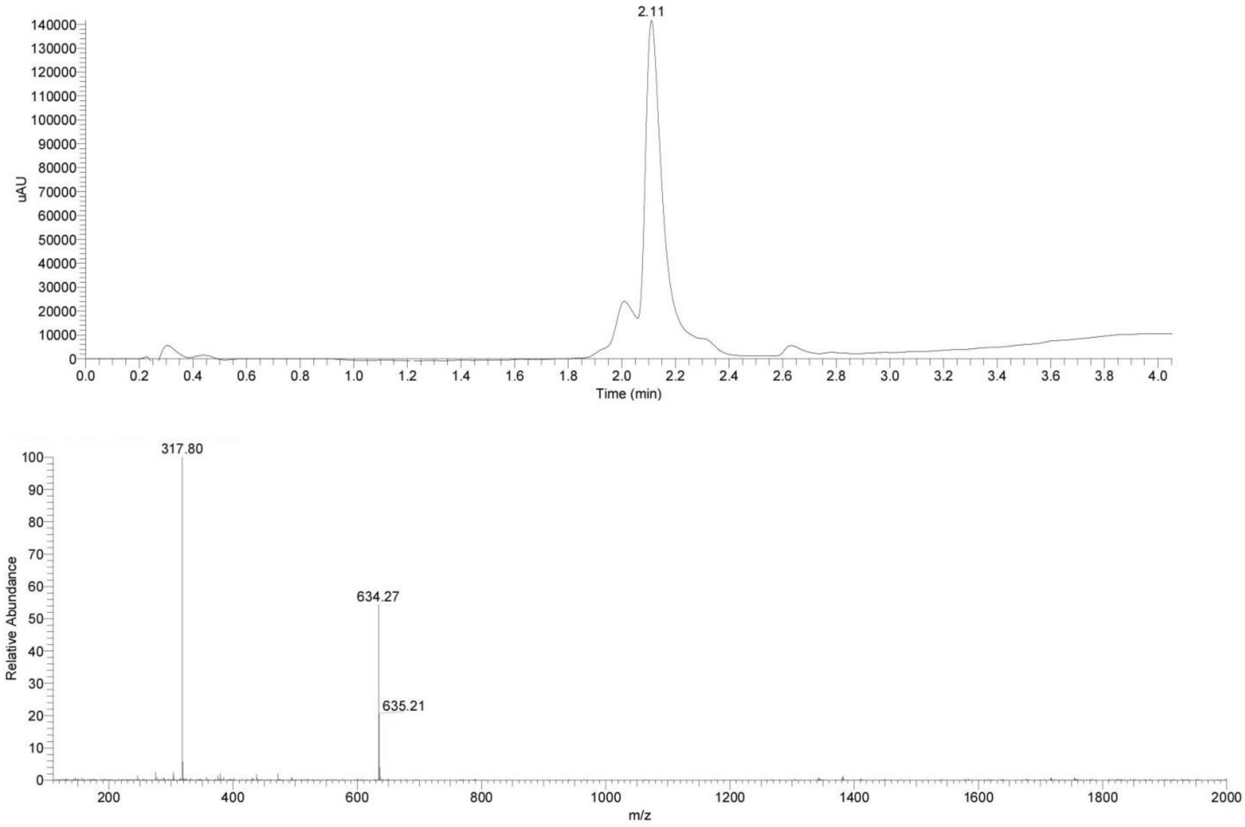

Supplementary Figure 26. LC-MS trace of compound **15**.

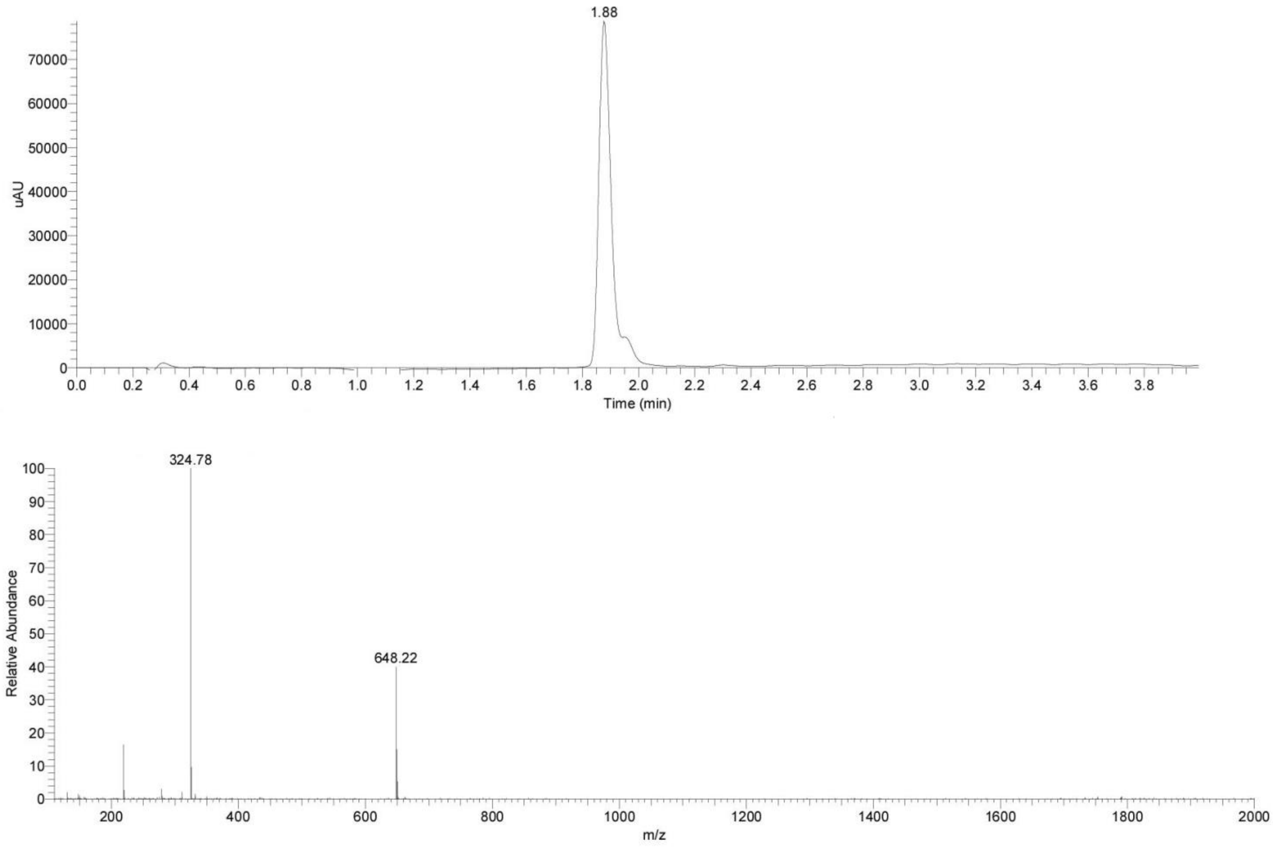

Supplementary Figure 27. LC-MS trace of compound **16**.

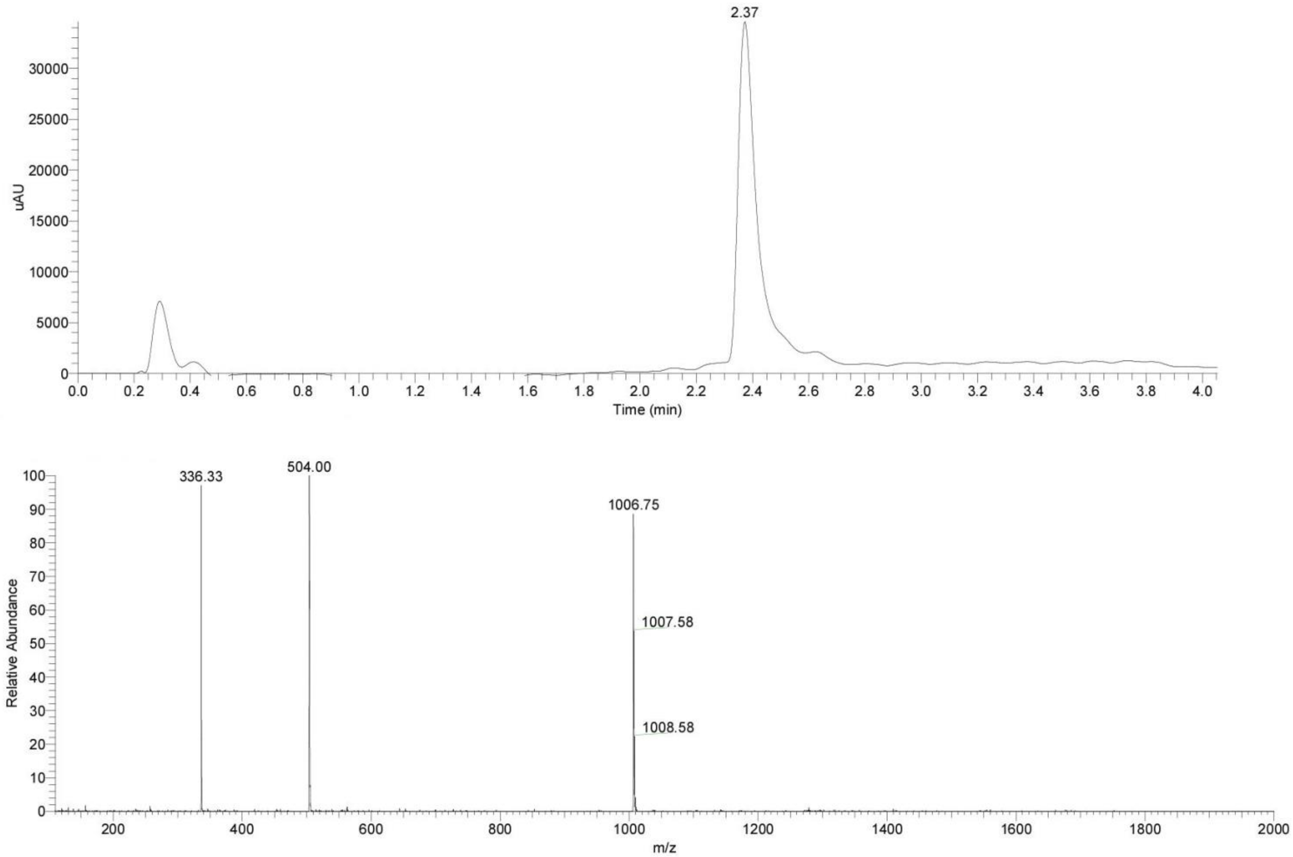

Supplementary Figure 28. LC-MS trace of compound **9**.

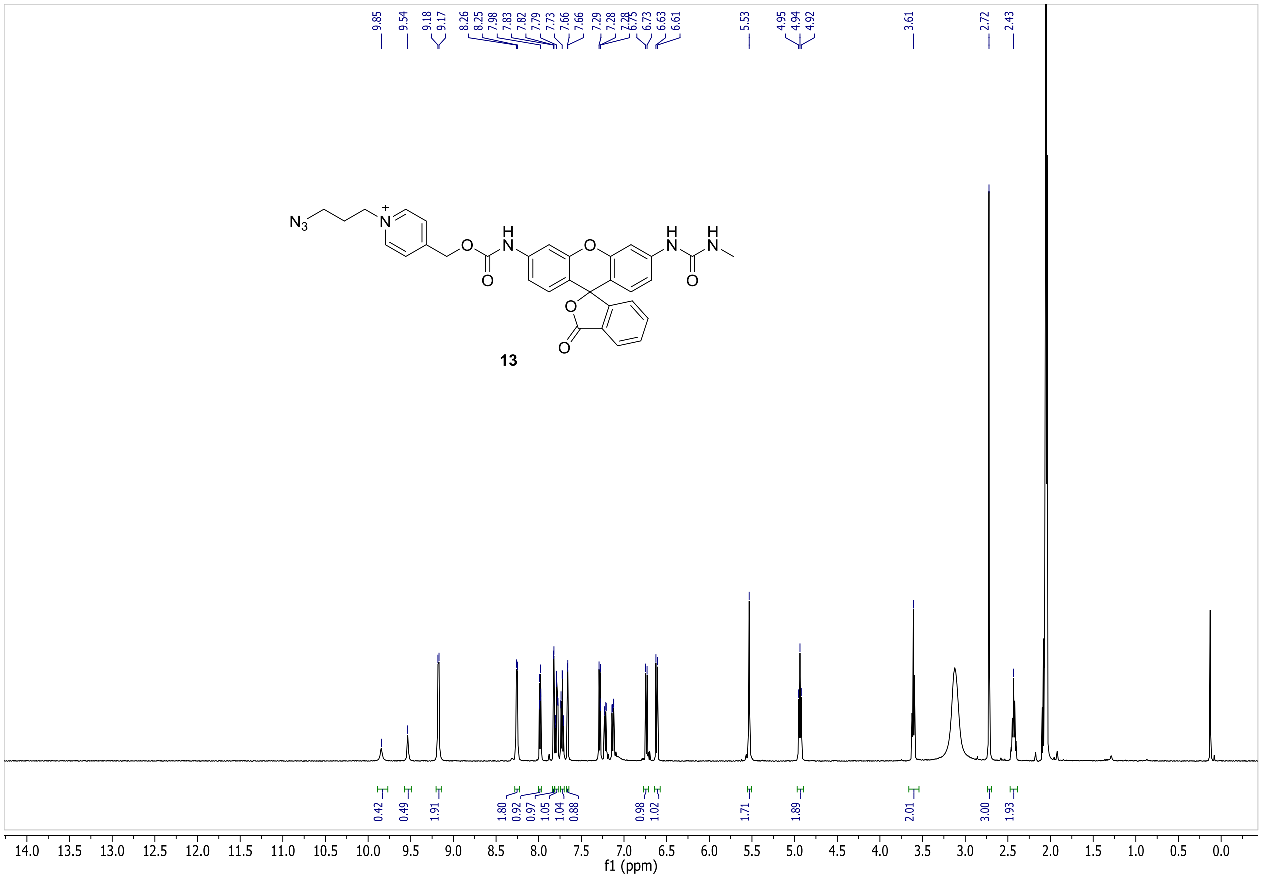

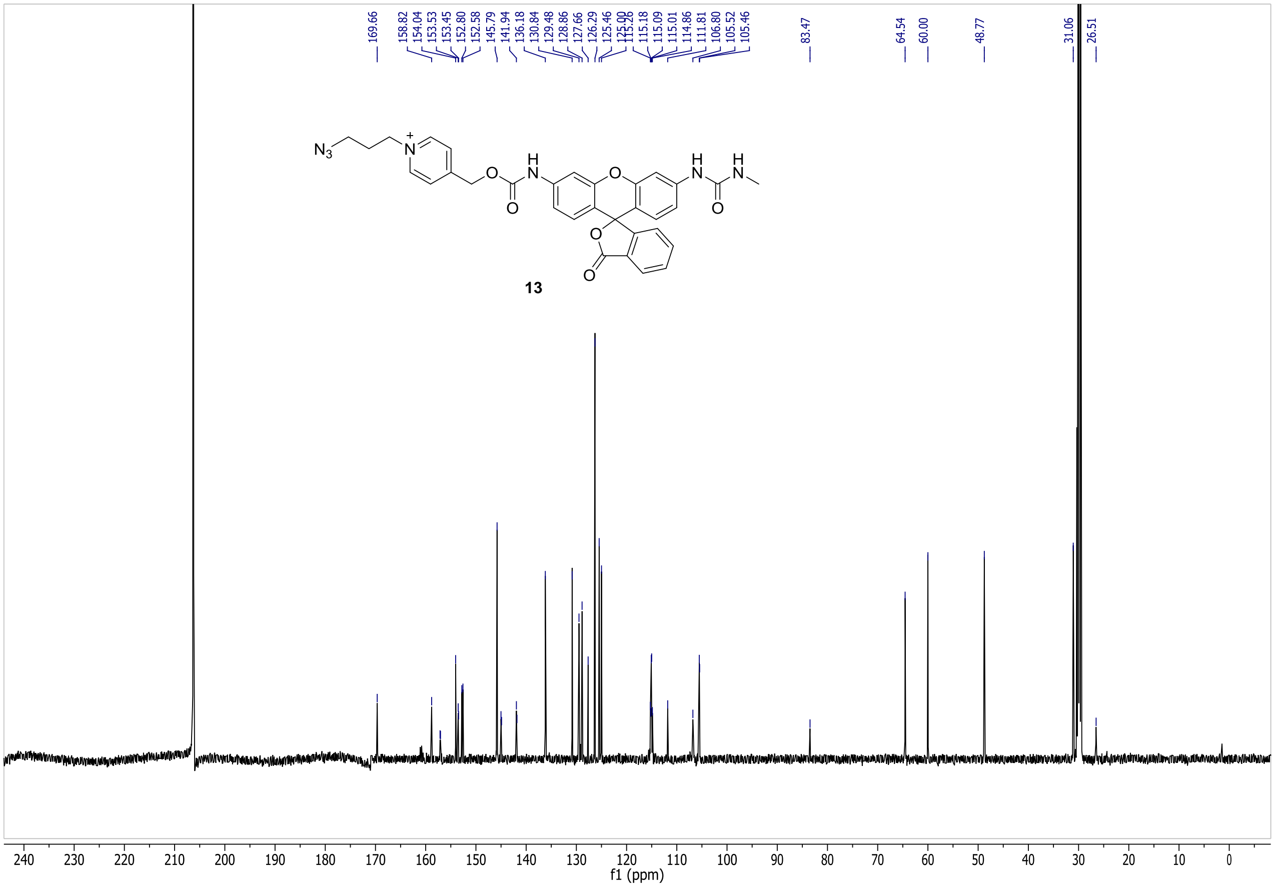

Supplementary Figure 29. ^{1}H and ^{13}C NMR of compound **13**.

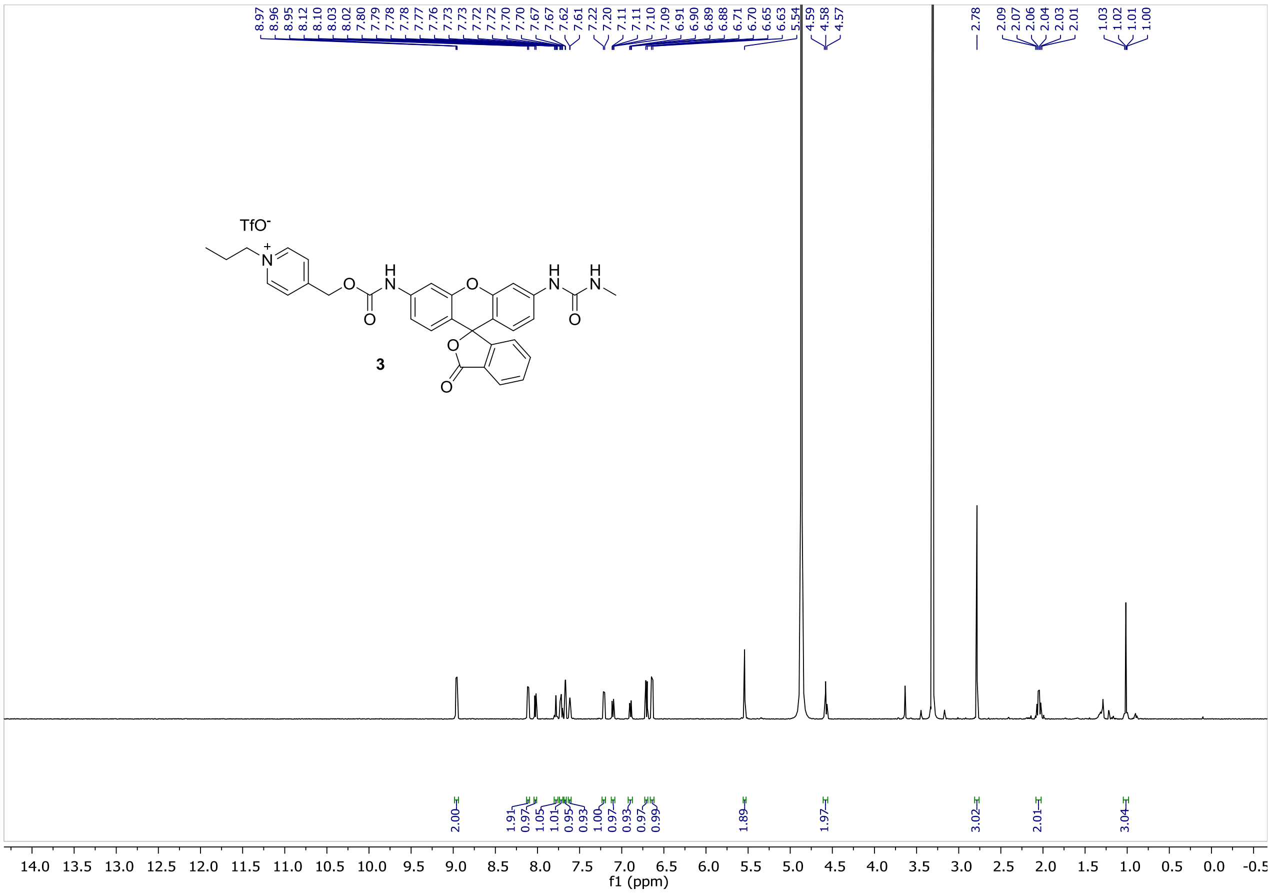

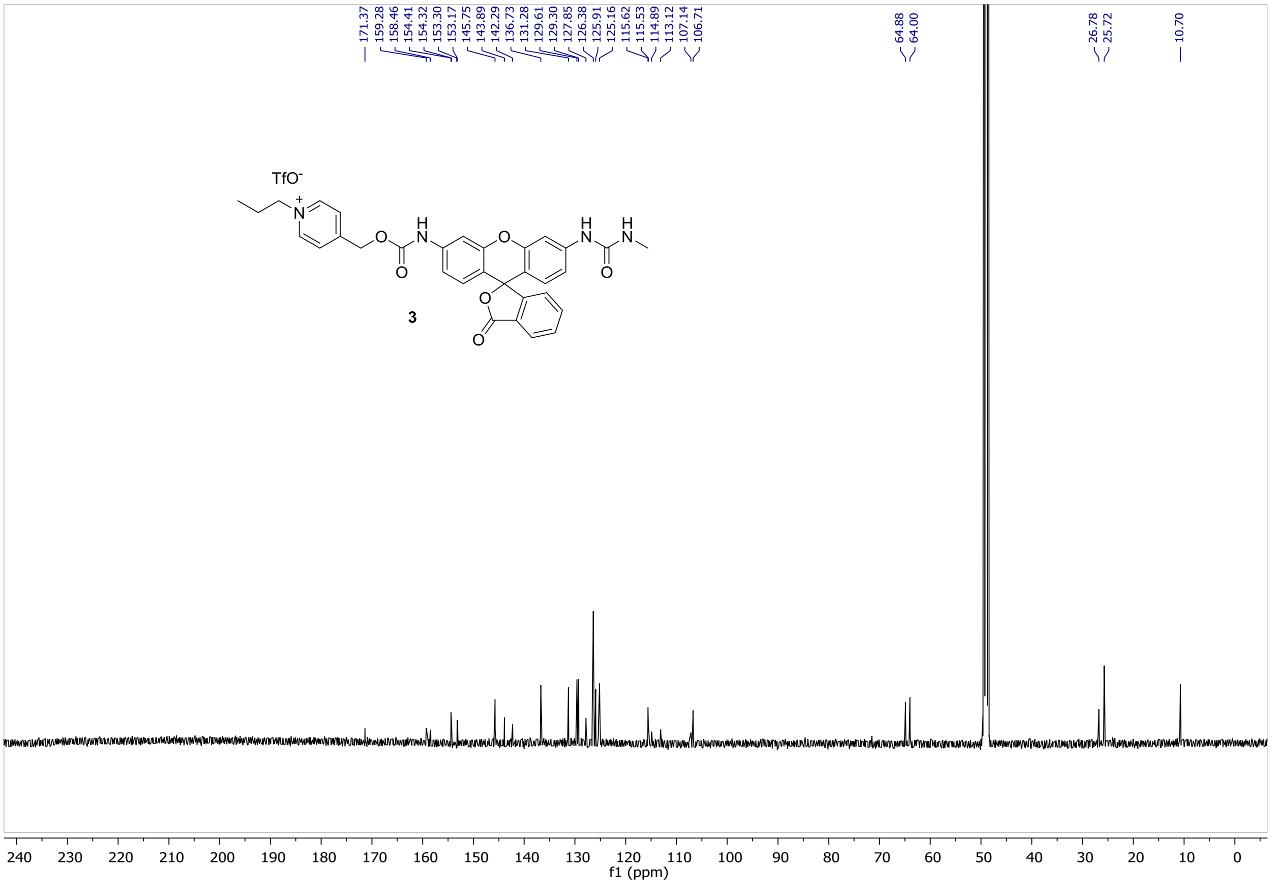

Supplementary Figure 30. ^{1}H and ^{13}C NMR of compound **3**.

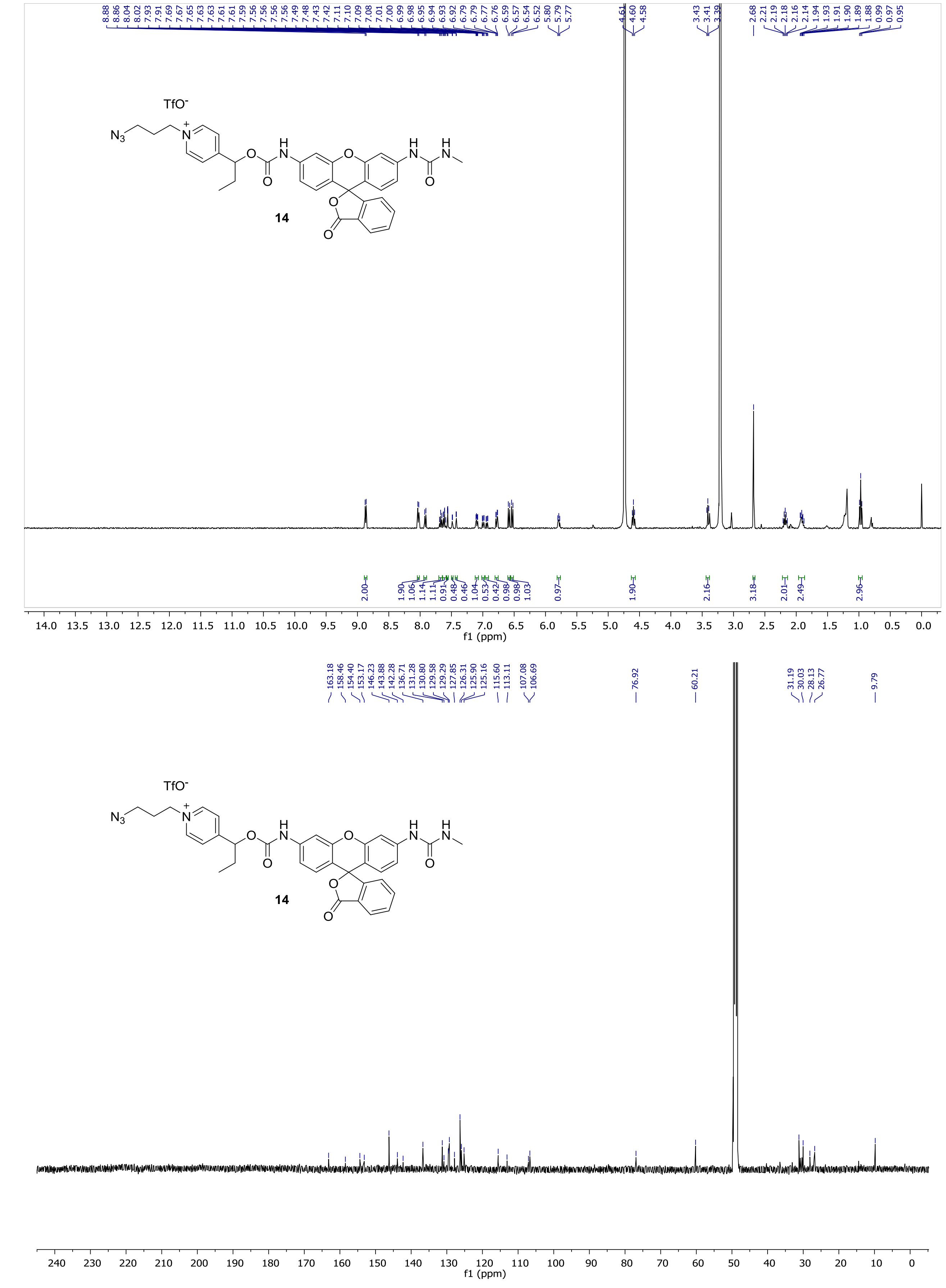

Supplementary Figure 31. ^{1}H and ^{13}C NMR of compound **14**.

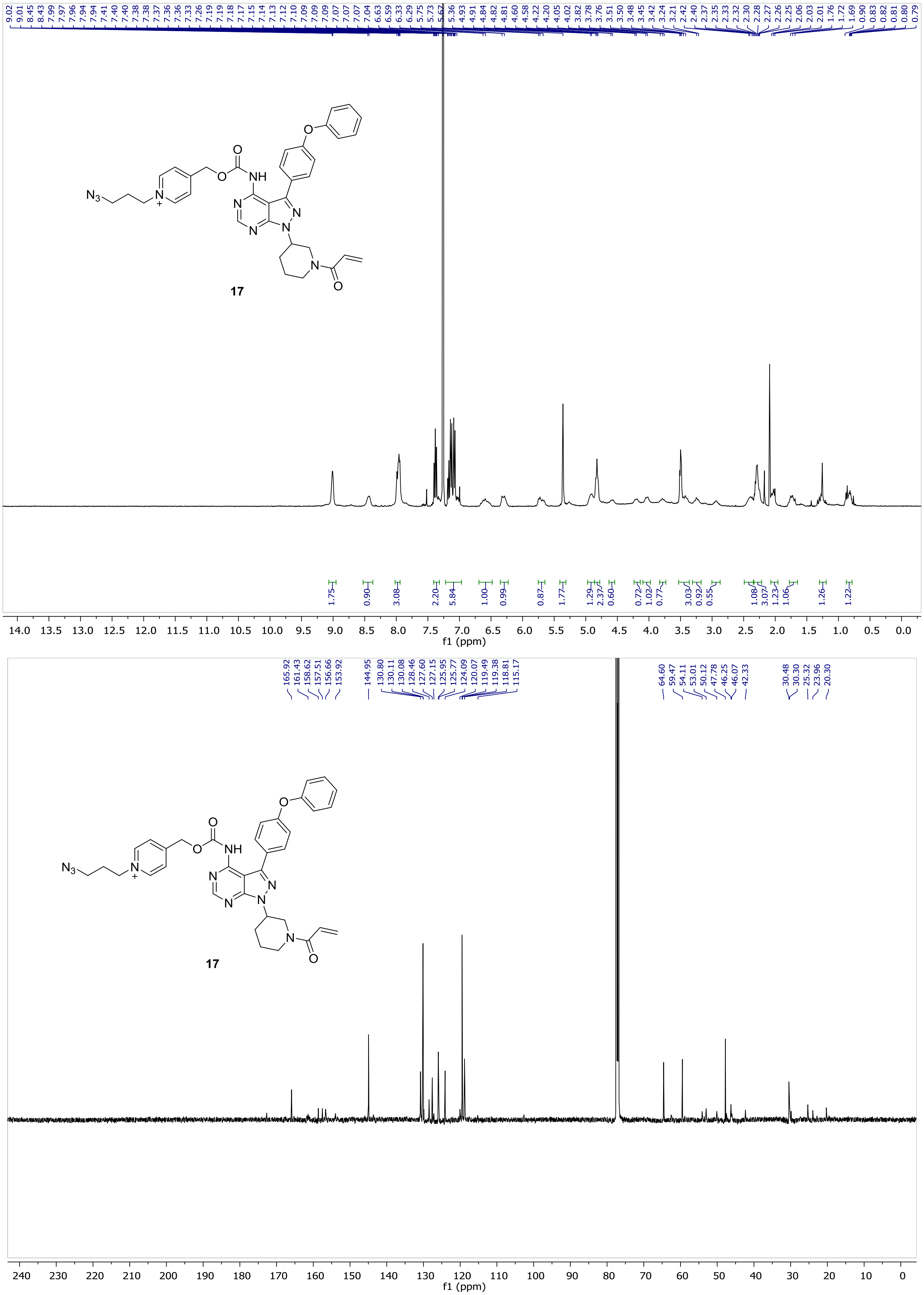

Supplementary Figure 32. ^{1}H and ^{13}C NMR of compound **17**.

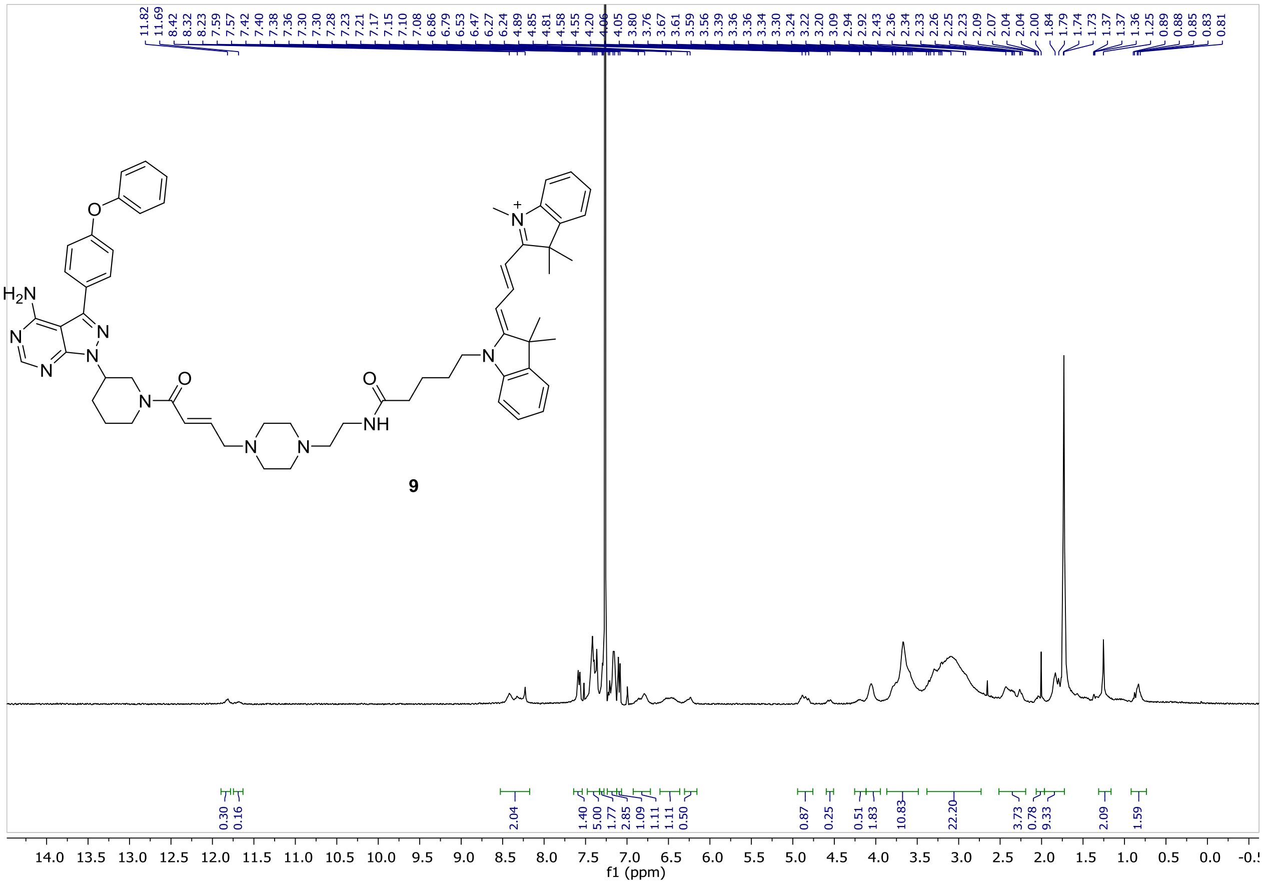

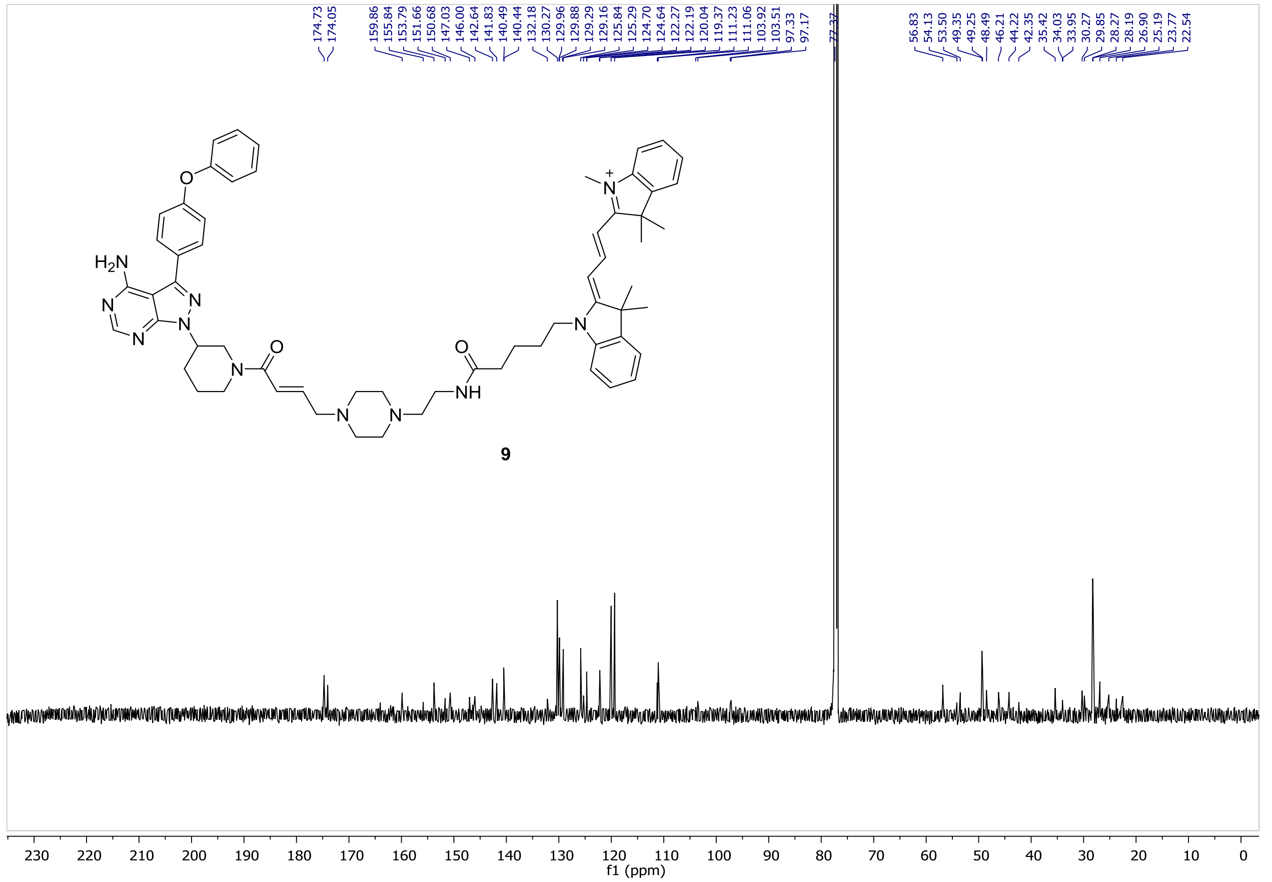

Supplementary Figure 33. ^{1}H and ^{13}C NMR of compound **9**.

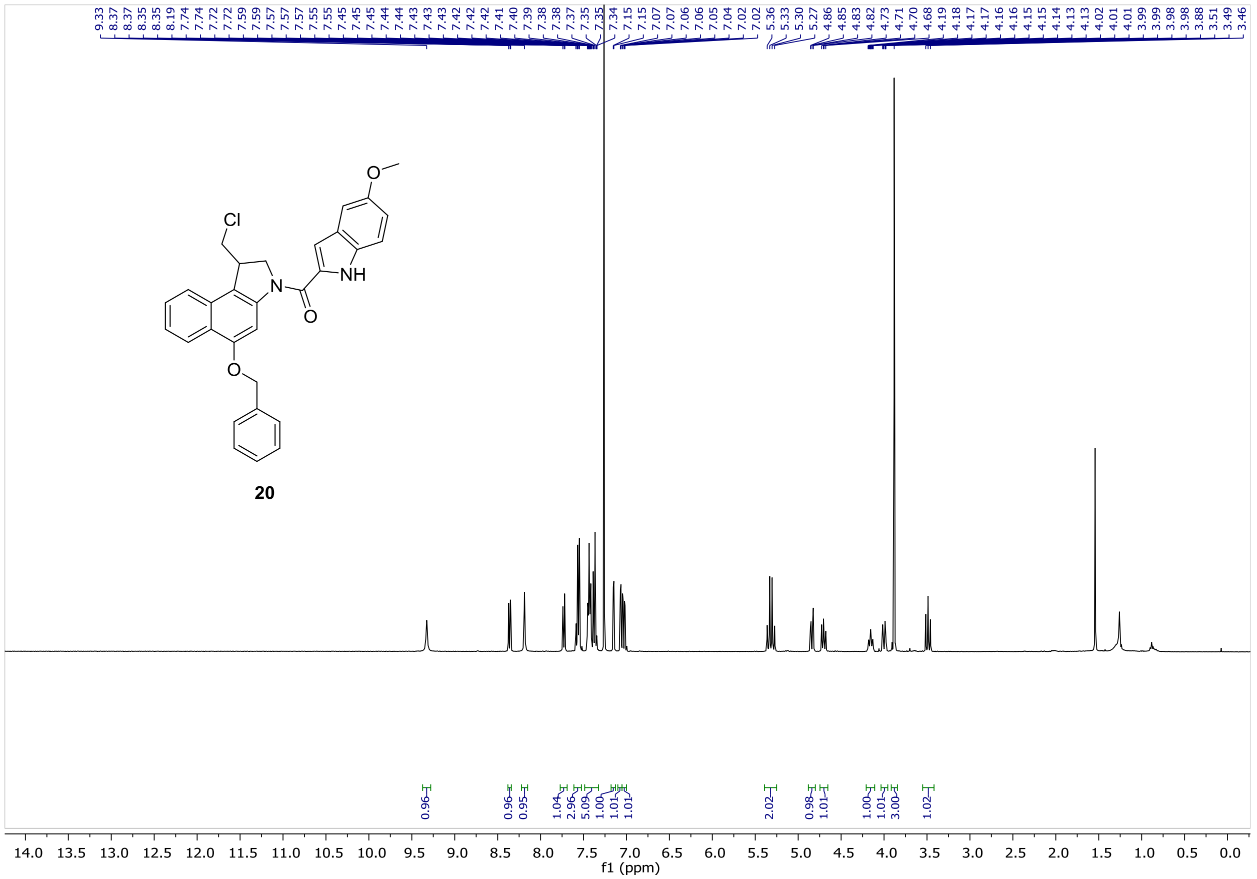

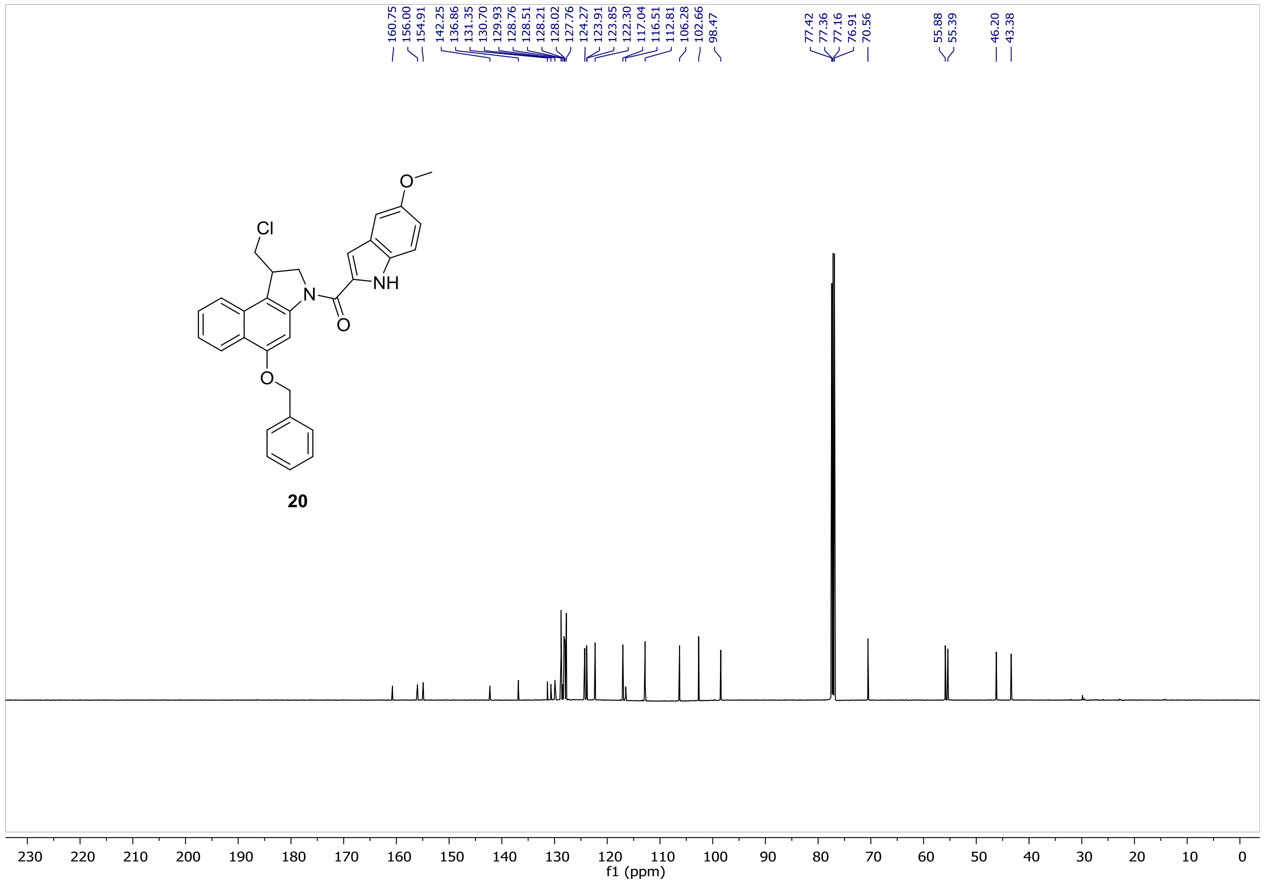

Supplementary Figure 34. ^{1}H and ^{13}C NMR of compound **20**.

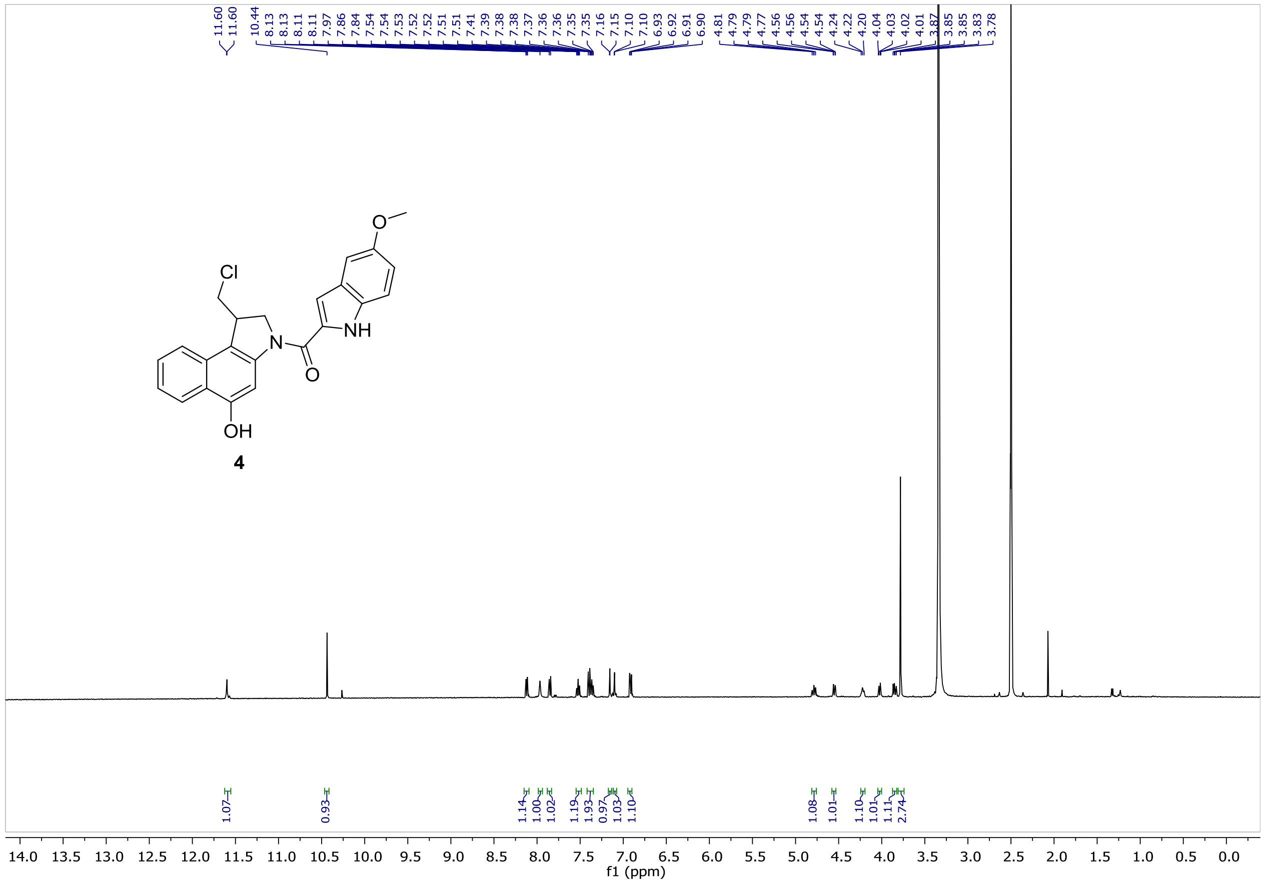

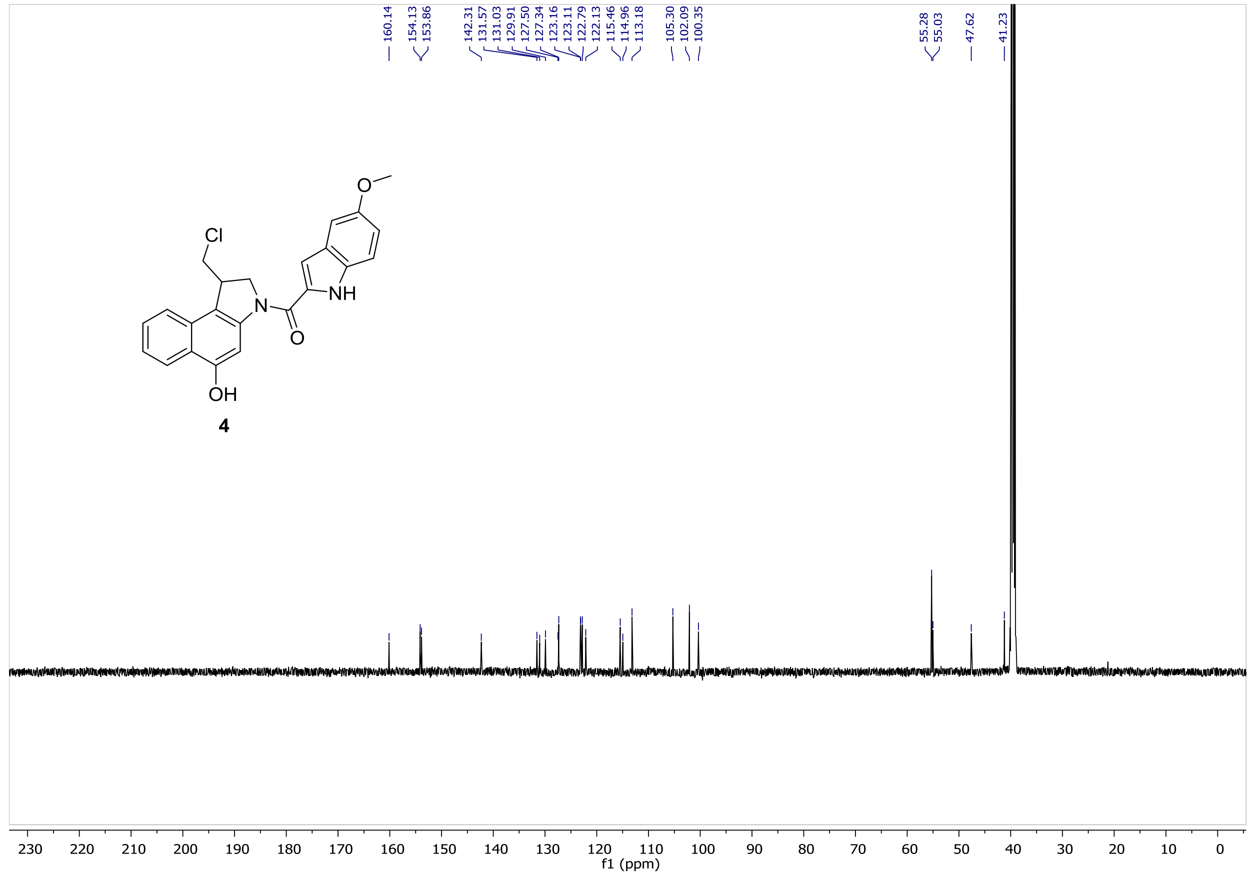

Supplementary Figure 35. ^{1}H and ^{13}C NMR of compound **4**.

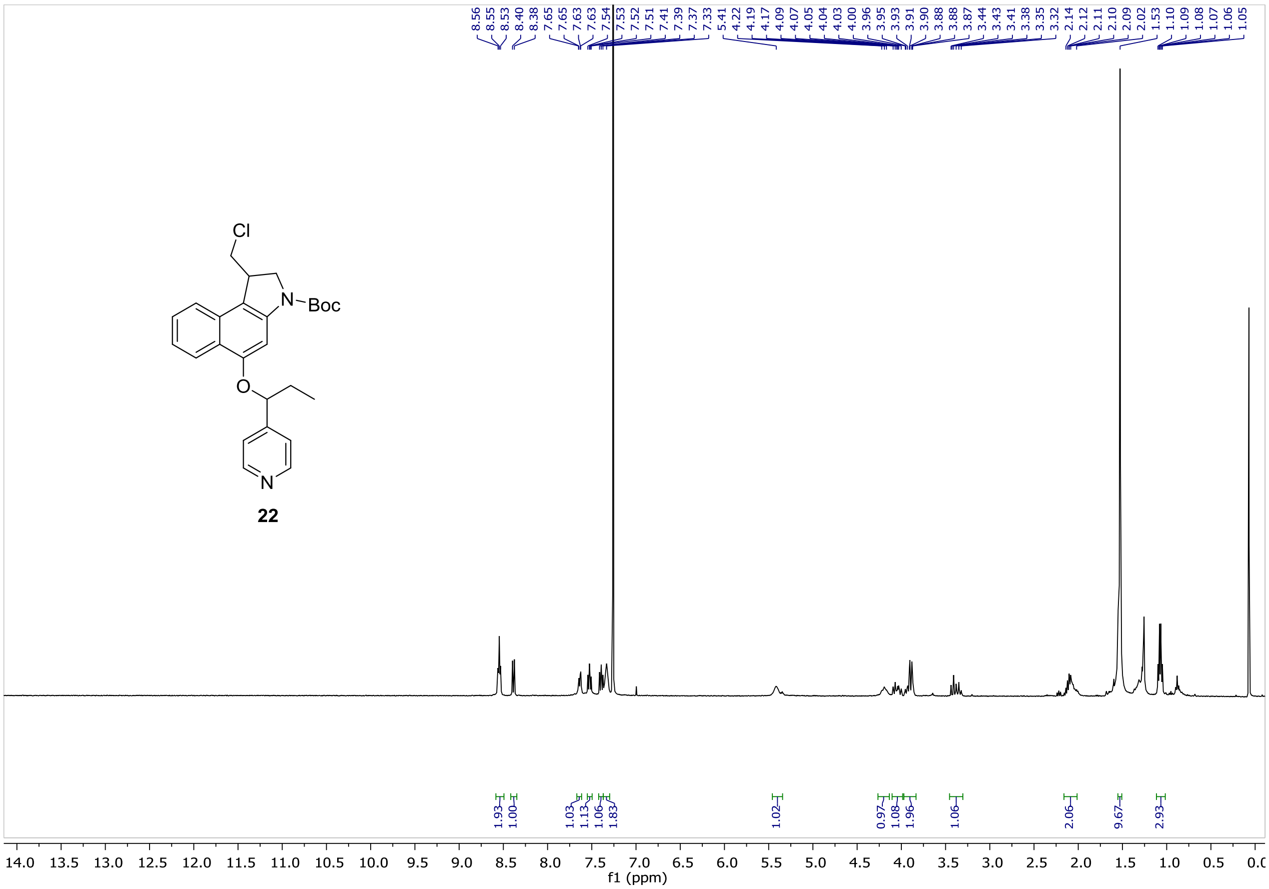

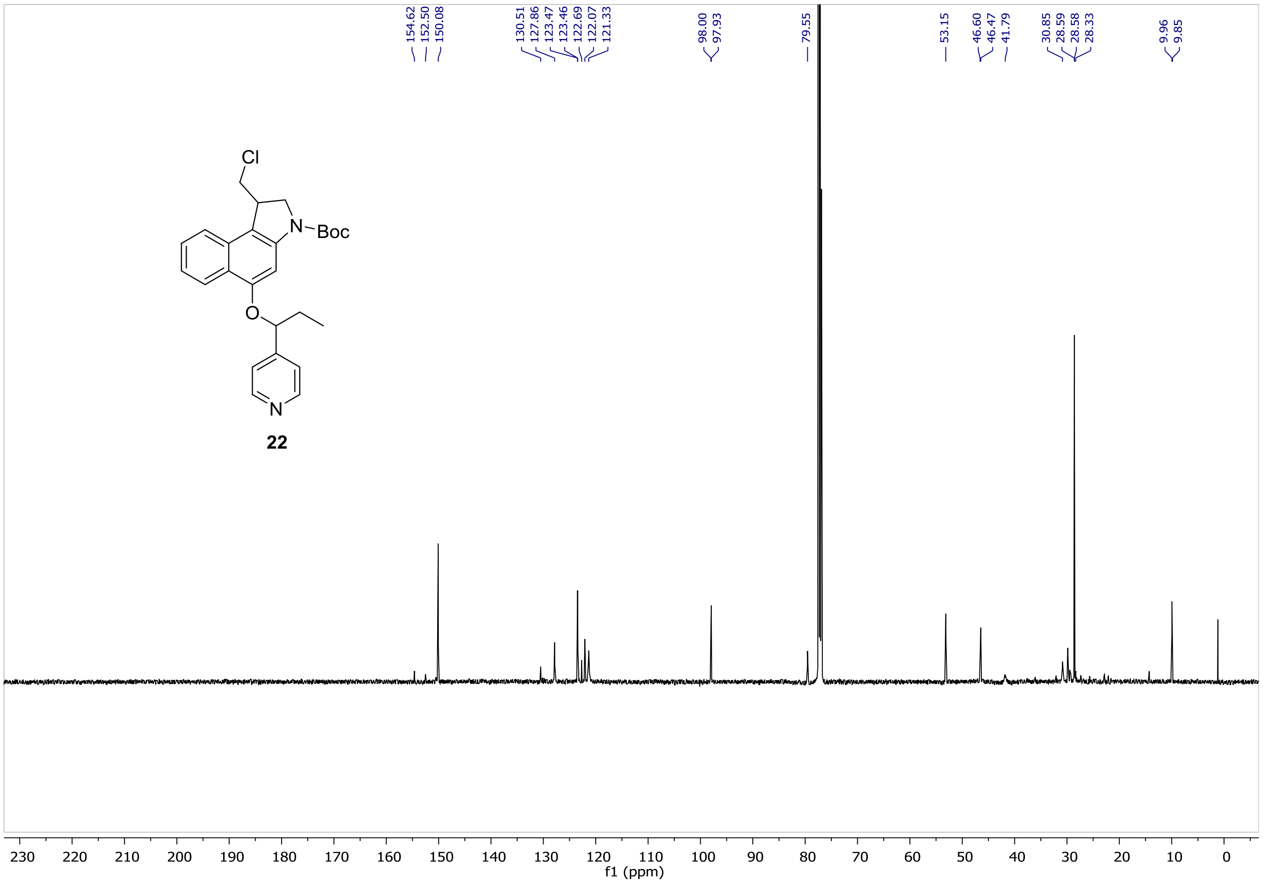

Supplementary Figure 36. ^{1}H and ^{13}C NMR of compound **22**.

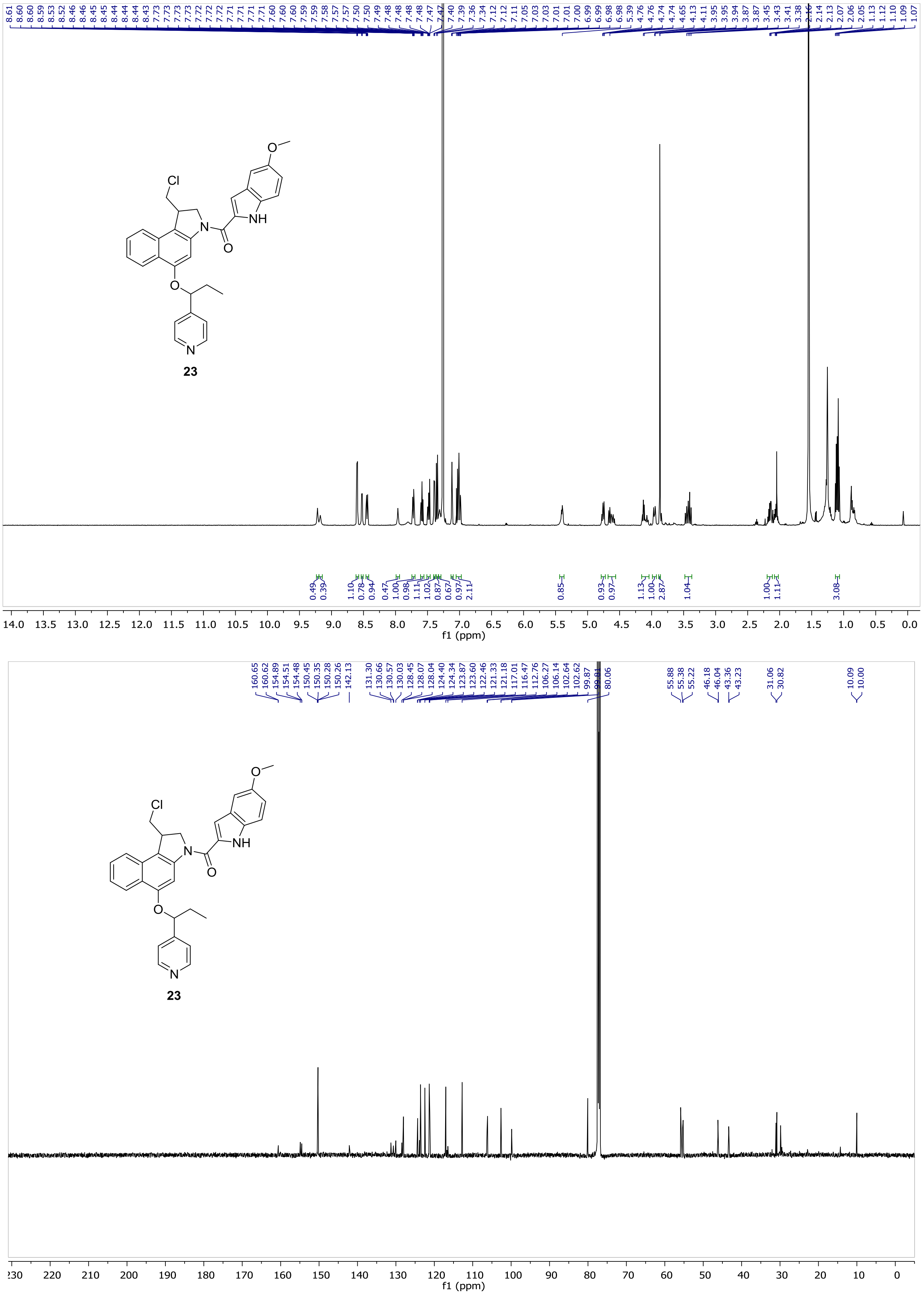

Supplementary Figure 37. ^{1}H and ^{13}C NMR of compound **23**.

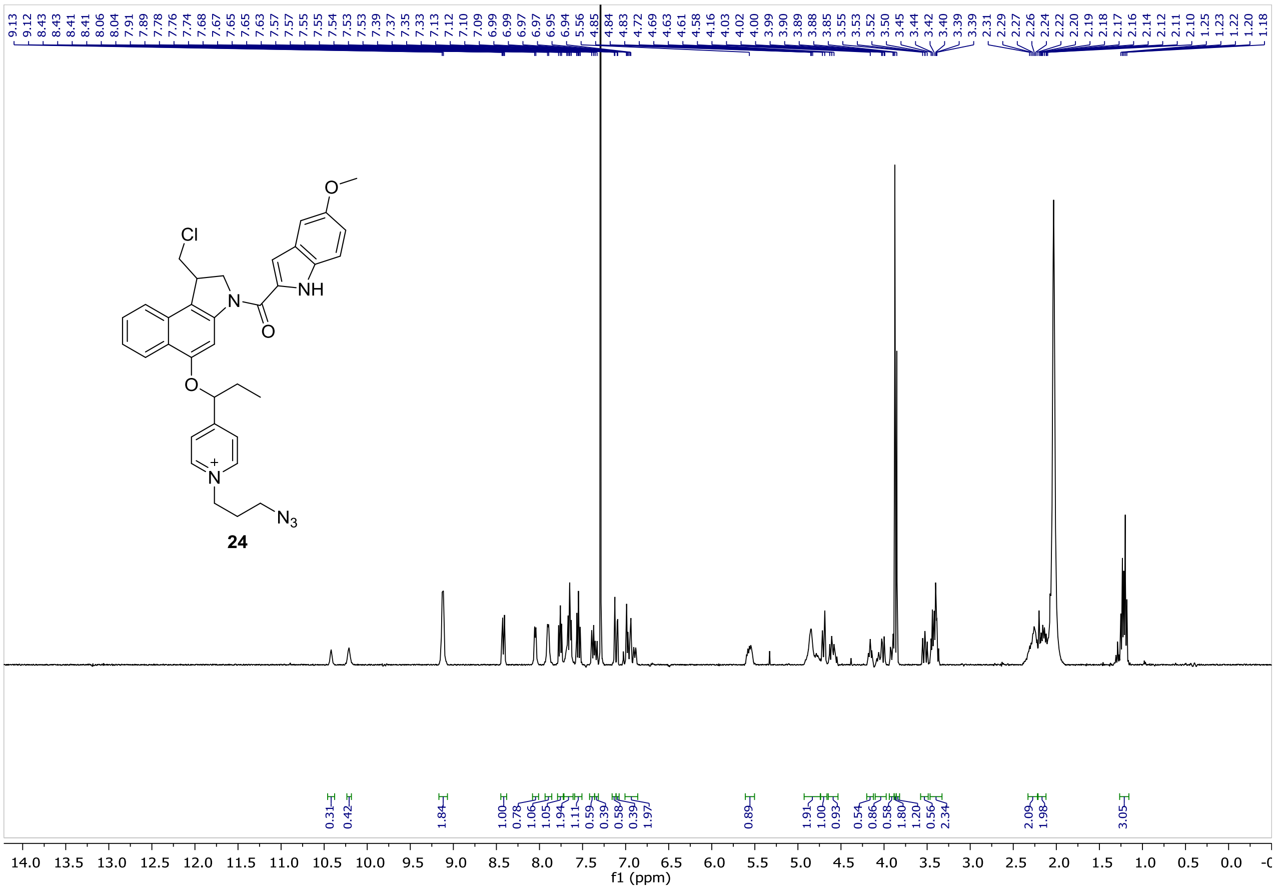

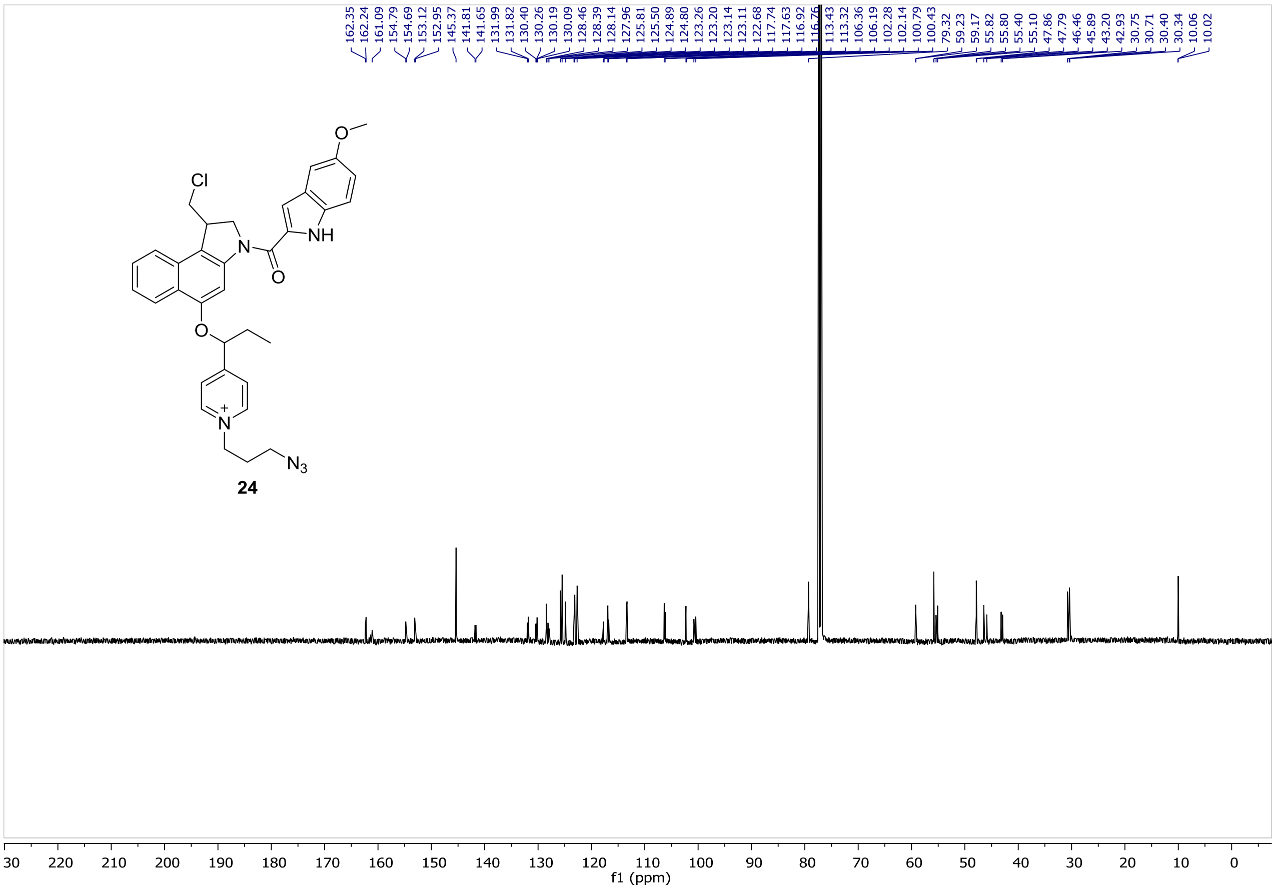

Supplementary Figure 38. ^{1}H and ^{13}C NMR of compound **24**.

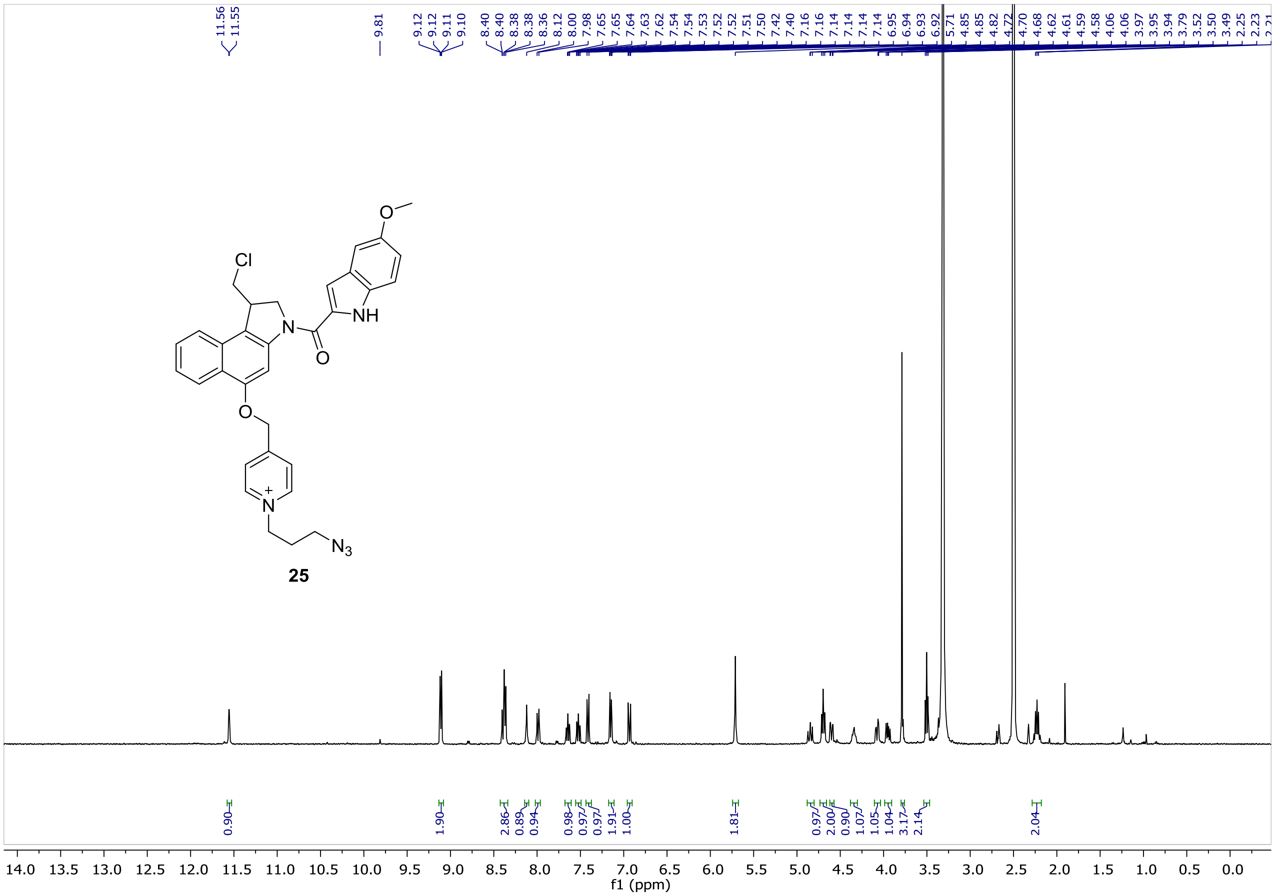

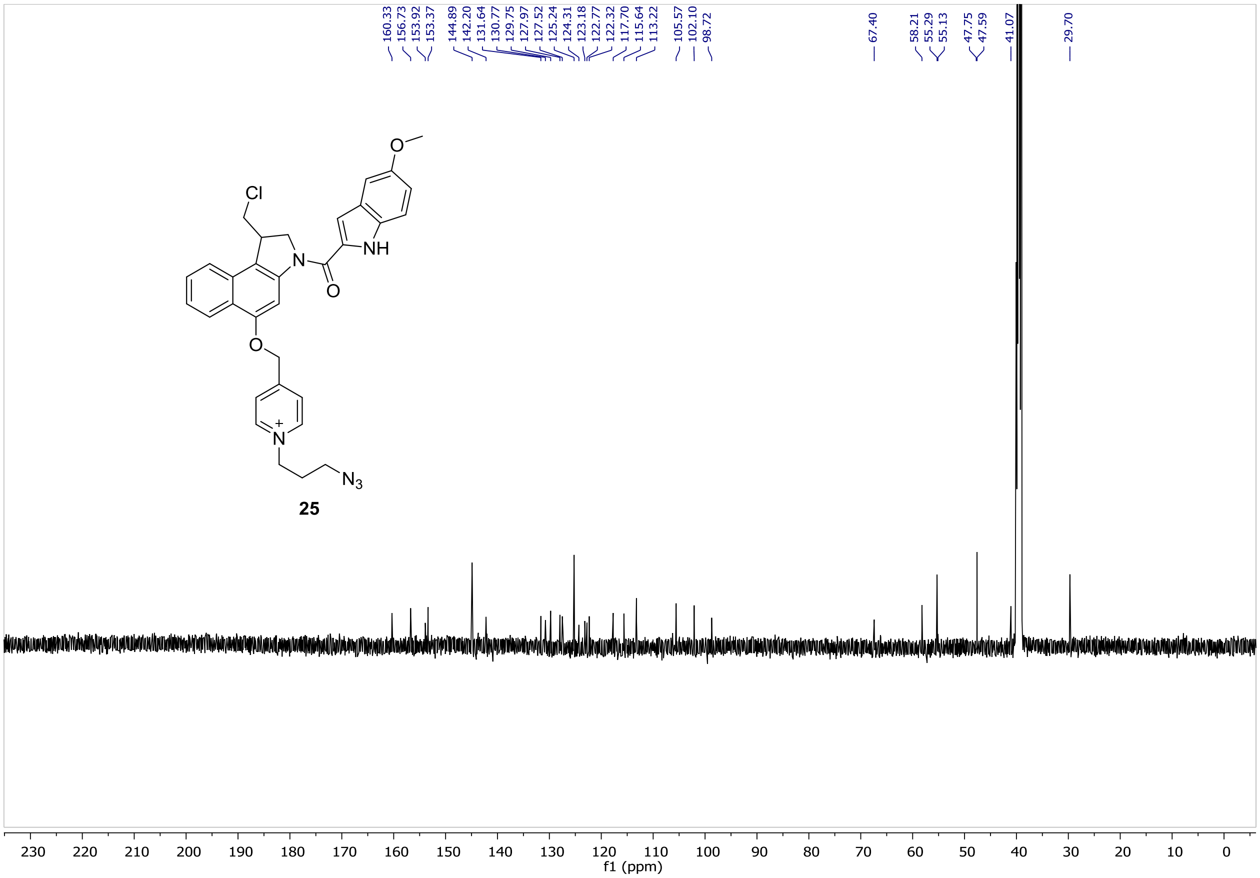

Supplementary Figure 39. ^{1}H and ^{13}C NMR of compound **25**.

Supplementary Methods

All reagents and solvents were purchased from commercial sources and were used without further purification. Anhydrous solvents were obtained by passing them through commercially available alumina columns (Innovative Technology, Inc., ® VA). All the reactions were carried out under nitrogen. Reverse phase column chromatography was performed using Isolera Biotage using SNAP Cartridge KP-C18-HS of 60 g or 12 g. HPLC purification was performed with an Agilent Technologies 1260 infinity HPLC using a ZORBAX 300SB-C18 column (9.4 x 250 mm). NMR spectra were recorded on Bruker 300 UltraShield for 300 MHz, Bruker AVANCE 3 HD for 400 MHz and Bruker 500 UltraShield for 500 MHz using $CDCl_3$, DMSO-*d6*, acetone-*d6* and Methanol-*d*4 as reference solvent. LC-MS spectra were recorded using a DIONEX Ultimate 3000 UHPLC coupled with a Thermo LCQ Fleet Mass Spectrometer System (electrospray ionization (ESI)) operated in positive mode. MALDI-TOF mass spectra were measured using a Bruker Daltonics Autoflex spectrometer. Fluorescence and luminescence intensities were measured using a Molecular Devices Spectra Max M5. Automated solid phase synthesis was carried out on an Intavis AG Multipep RS instrument. Fluorescence imaging was carried out using a Leica SP8. Cell counting and imaging of cells was carried out using a Molecular Devices™ ImageXpress Micro (IXM) XL automated microscope. Furimazine[1], 4-(bromomethyl)pyridine hydrobromide[2], 3-azidopropyl trifluoromethanesulfonate[3], bis(2,2′-bipyridine)-(5-amine-phenanthroline)ruthenium bis-(hexafluorophosphate)[4] , benzyl guanine[5, 6], raloxifene[7] and 3,5-disulfobenzoic acid sodium salt[8] were prepared according to cited procedures. Methotrexate was synthesized from 2,4-diamino-6-(hydroxymethyl)pteridine as previously described.[9, 10] SNAP-Pro_{30}-NLuc-cpDHFR plasmid was a kind gift from Kai Johnsson and was expressed and purified as previously described.[11]

Calculation of Förster distance between Nanoluc and $Ru(bpy)_2$phen

$$R_0 = 0.211\left(\kappa^2 n^{-4}\, Q_D\, J(\lambda)\right)^{\frac{1}{6}}$$

Orientation factor (κ^2) and refractive index (*n)* was assumed to have a value of 2/3 and 1.36 respectively.[12] Quantum yield of NanoLuc (Q_D) has been reported as 0.28.[13] The spectral overlap integral between the luminescence spectrum of SNAP-Pro_{30}-NLuc-cpDHFR and the absorption spectrum of $[Ru(bpy)_2phen\text{-}NH_2]^{2+}$ ($J(\lambda)$) was calculated as 4.041 x 10^{12} M^{-1} cm^{-1} $(nm)^4$ using a|e software from Fluortools.[14]

R_0 = 16.4±0.9 Å

Calculation of energy transfer efficiency

$$E_{RET} = 1 - \left(\frac{I_{DA}}{I_D}\right)$$

I_{DA} = donor emission in the presence of acceptor.
I_D = donor emission in the absence of acceptor.

Formula used as described.[12]

Calculated E_{RET} for NLuc - ruthenium = 0.64±0.08

Automated PNA synthesis.[15] Solid phase syntheses were performed with NovaPEG Rink amide resin obtained from EMD Millipore. PNAs were synthesized in 500 mL fritted tubes by using an Intavis MultiPep instrument in a fully automated fashion. The resin (5 mg 0.44 mmol/g, 2.2 µmol) was swollen in CH_2Cl_2 (300 µL) for 20 min then washed with DMF and CH_2Cl_2 and treated with a pre-activated (5 min) solution of the corresponding Mtt-protected PNA monomer (5 equiv), HATU (4 equiv), DIPEA (5 equiv), and 2,6-lutidine(7.5 equiv) for 20 min in NMP. This process was repeated once (double couplings). Each coupling was followed by a capping step with Ac_2O (5.3 equiv) and 2,6-lutidine (6.4 equiv) in DMF (150 µL per column).

Fmoc deprotection resin was treated with 20% piperidine in DMF (10 min x 2) followed by washing of resin with DCM, DMF and DCM.

Mtt deprotection.
The resin was treated with a solution of HOBt in a 1:1 mixture of hexafluoroisopropanol and 1,2-dichloroethane (200 µL / column) for 3 min, washed with DCM, this process was repeated 3 times after which the resin was washed with DCM, DMF and DCM.

General method for coupling of primary amine with carboxylic acid.
Carboxylic acid (Fmoc-O_2O_C-OH; Fmoc-Lys(Mtt)-OH; Fmoc-Gly-OH; Mtx(OtBu)-OH)(5 eq.) was preactivated in NMP with HATU (4.5 eq.), DIPEA (4 eq.) and 2,6-lutidine (4 eq.) for 10 mins. The activated carboxylic acid was then added to the resin and left for 10 min after which the coupling was repeated. The resin was then washed with DCM, DMF and DCM, followed by capping for 5 min and washed again with DCM, DMF and DCM.

General technique for PNA purification. Final compounds were purified using an Agilent 1100 series HPLC equipped with DAD and with a Agilent ZORBAX Eclipse XDB-C18 column (4.6 x 250 mm, 5µm); linear gradient from 100% H_2O 0.1% TFA to 100% MeCN 0.1% TFA with a flow rate of 3 mL/min).

Synthesis of BG-(Ru)(PNA)-MTX (1).
1 was synthesized as shown in Supplementary Figure 1 by general methods described above. Specific reactions not included in the general methods are described below.
i) Ruthenium coupling: the Mtt was removed with a solution of HOBt in a 1:1 mixture of HFIP and DCE (200 µL) and the resin was washed with DMF and CH_2Cl_2. In a separate Eppendorf, bis(2,2′-bipyridine)-(5-amine-phenanthroline)ruthenium bis-(hexafluorophosphate) (8.3 mg, 11 µmol, 5.0 equiv) and triphosgene (3.26 mg, 11 µmol, 5.0 equiv) were added in 100 µL DCE, then DIPEA (19.2 µL, 0.11 mmol, 50 equiv) was added and kept for a few minutes until the solution became clear, the solution was added to the resin and swirled for 10 h. The resin was washed with DMF and DCM.
ii) Coupling of benzyl guanine to linker: to the purified linker in DMF was added BG-GLA-NHS (1.5 eq.)[5, 6] and DIPEA (5 eq.) and the reaction was left for 12 hours after which **1** was isolated via RP-HPLC. **1** was dissolved in DMSO and stored in aliquots (0.4 mM) at -80 °C.

Preparation of ruthenium derivative

PNAs (5 mg 0.44 mmol/g, 2.2 µmol) with Lysine in the N-terminus were treated with 20% piperidine in DMF (200 µL, 30 min). The Mtt was removed with a solution of HOBt in a 1:1 mixture of HFIP and DCE (200 µL) and the resin was washed with DMF and CH_2Cl_2. In a separate Eppendorf, bis(2,2′-bipyridine)-(5-amine-phenanthroline)ruthenium bis-(hexafluorophosphate) (8.3 mg, 11 µmol, 5.0 equiv) and triphosgene (3.26 mg, 11 µmol, 5.0 equiv) were added in 100 µL DCE, then DIPEA (19.2 µL, 0.11 mmol, 50 equiv) was added and kept for a few minutes until the solution became clear, the solution was added to the resin and swirled for 10 h. The resin was washed with DMF and CH_2Cl_2. The resin was suspended in TFA (200 µL) for 2 h. The solution was filtered and the product precipitated with diethyl ether (2 mL, 10 times of the volume of TFA). The precipitate was pelleted by centrifugation and the supernatant was removed. The pellet was washed with diethyl ether then dissolved in 400 µL H_2O for HPLC purification as described in the general technique for PNA purification.

Preparation of PNA-Pyridinium derivatives. PNAs (5 mg 0.44 mmol/g, 2.2 µmol) with N-terminal Lysine were treated with a solution of 20% piperidine in DMF (200 µL, 30 min), then capped with a solution of Ac_2O (5.3 equiv) and 2,6-lutidine (6.4 equiv) in DMF (150 µL). Mtt group was removed with a solution of HOBt in a 1:1 mixture of HFIP and DCE (200 µL) for 3 x 3 min. The resin was then treated with a pre-activated (5 min) solution of 4-pentynoic acid (5 equiv), HATU (4 equiv), DIPEA (5 equiv) and 2,6-lutidine (7.5 equiv) for 60 min in NMP. After the reaction, the resin was washed with DMF and CH_2Cl_2. A solution of small molecule (**3**, **13**, **14**, **15**, **16**, **17**) (1.5 equiv in 200 µL NMP) was added to the resin followed by a solution of sodium ascorbate in H_2O (16.6 µL, 198 mg/mL, 16.5 µmol, 7.5 equiv), a solution of $CuSO_4$ in H_2O (4.2 µL, 21.4 mg/mL 0.55 µmol, 0.25 equiv) and TBTA (0.6 mg, 0.5 equiv). After 16 hours, the resin was washed with H_2O (6 x 250 µL), DMF (6 x 250 µL), and CH_2Cl_2 (6 x 250 µL). The resin was suspended in TFA (200 µL) for 2 h; the solution was filtered and the product precipitated with diethyl ether (2 mL, 10 times of the volume of TFA). The precipitate was pelleted by centrifugation and the supernatant was removed. The pellet was washed with diethyl ether, then dissolved in 400 µL H_2O for HPLC purification as described in the general technique for PNA purification.

Preparation of sulfonated PNA-Pyridinium derivatives. PNAs (5 mg 0.44 mmol/g, 2.2 µmol) with N-terminal Lysine were treated with a solution of HOBt in a 1:1 mixture of HFIP and DCE (200 µL) for 3 min (x 3) and then the resin was washed and treated with a pre-activated (5 min) solution of 4-pentynoic acid (5 equiv), HATU (4 equiv), DIPEA (5 equiv) and 2,6-lutidine (7.5 equiv) for 20 min in NMP twice. After the reaction, the resin was washed with DMF and CH_2Cl_2. The Fmoc group was removed with a solution of 20% piperidine in DMF (200 µL, 30 min). 3,5-disulfobenzoic acid (11 µmol, 5 equiv), HATU (4 equiv), DIPEA (5 equiv) and 2,6-lutidine (7.5 equiv) in 150 µL of DMF was added to the resin and swirled for 1 hour. After washings with H_2O, DMF and CH_2Cl_2, a solution of small molecule (**24, 25**) (1.5 equiv in 200 µL of NMP) was added to the resin followed by a solution of sodium ascorbate in H_2O (16.6 µL, 198 mg/mL, 16.5 µmol, 7.5 equiv), a solution of $CuSO_4$ in H_2O (4.2 µL, 21.4 mg/mL 0.55 µmol, 0.25 equiv) and TBTA (0.6 mg, 0.5 equiv). After 16 hours, the resin was washed with H_2O (6 x 250 µL), DMF (6 x 250 µL), and CH_2Cl_2 (6 x 250 µL). The resin was suspended in TFA (200 µL) for 2 h; the solution was filtered and the product precipitated with diethyl ether (2 mL, 10 times of the volume of TFA). The precipitate was pelleted by centrifugation and the supernatant was removed. The pellet was washed with diethyl ether, then dissolved in 400 µL H_2O for HPLC purification as described in the general technique for PNA purification.

PNA sequences

Sequence: BG-$(PEG)_3$-Lys-Gly-Lys(Ru)-Lys(A*GT*TG*)-PEG-MTX **1**

Chemical Formula: $C_{175}H_{222}N_{62}O_{44}Ru^{2+}$, Exact Mass: 3997.61, **LC-MS (ESI^+)** RT= 1.49 min. *m/z* found: 1333.08 $[M+3H]^{3+}$, 1000.33 $[M+4H]^{4+}$, 800.58 $[M+5H]^{5+}$, 667.08 $[M+6H]^{6+}$; **MALDI-TOF** calculated *m/z*: 3998.611 (100%), *m/z* found: 3999.834 $[M+H]^+$.

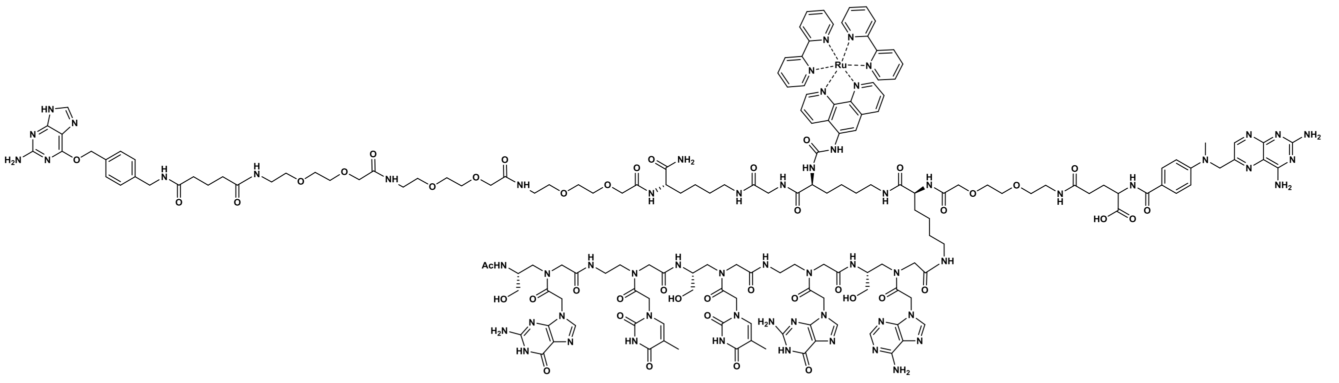

Sequence: Lys-C*AA*CT*-Pyridinium-Rhodamine (PNA-PyRho) **2**

Chemical Formula: $C_{101}H_{121}N_{38}O_{26}^+$, Exact Mass: 2281.32, **LC-MS (ESI^+)** RT= 1.58 min. *m/z* found: 1142.17 $[M+H]^{2+}$, 761.92 $[M+2H]^{3+}$, 571.83 $[M+3H]^{4+}$; **MALDI-TOF** calculated *m/z*: 1852.834 (100%), *m/z* found: 1852.920 [M-Rhodamine]$^+$.

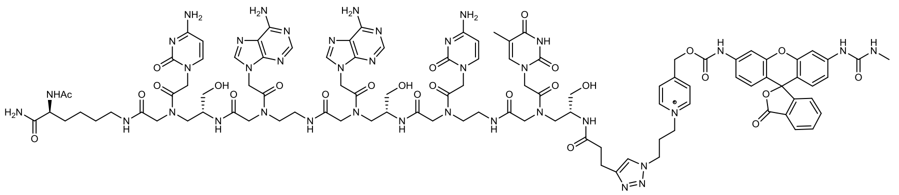

Sequence: (Ru)-Lys-A*GT*TG* **5**

Chemical Formula: $C_{100}H_{119}N_{39}O_{21}Ru^{2+}$, Exact Mass: 2259.78, **LC-MS (ESI^+)** RT= 1.27 min. *m/z* found: 1130.08 $[M]^{2+}$, 753.67 $[M+H]^{3+}$, 565.75 $[M+2H]^{4+}$, 452.75 $[M+3H]^{5+}$; **MALDI-TOF** calculated *m/z*: 2260.789 (100%), *m/z* found: 2262.101 $[M+H]^+$.

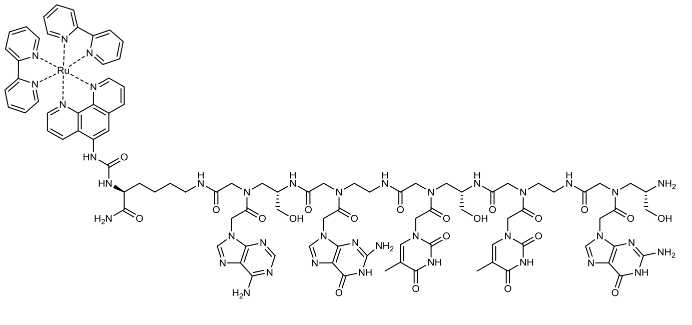

Sequence: Lys-C*AA*CT*-Pyridinium-Raloxifene (PNA-PyRal) **6**

Chemical Formula: $C_{106}H_{131}N_{36}O_{24}S^{+}$, Exact Mass: 2323.99, **LC-MS (ESI^{+})** RT= 1.51 min. *m/z* found: 1163.50 $[M+H]^{2+}$, 775.67 $[M+2H]^{3+}$, 582.17 $[M+3H]^{4+}$; **MALDI-TOF** calculated *m/z*: 1852.834 (100%), *m/z* found: 1854.294 $[M+H\text{-Raloxifene}]^{+}$.

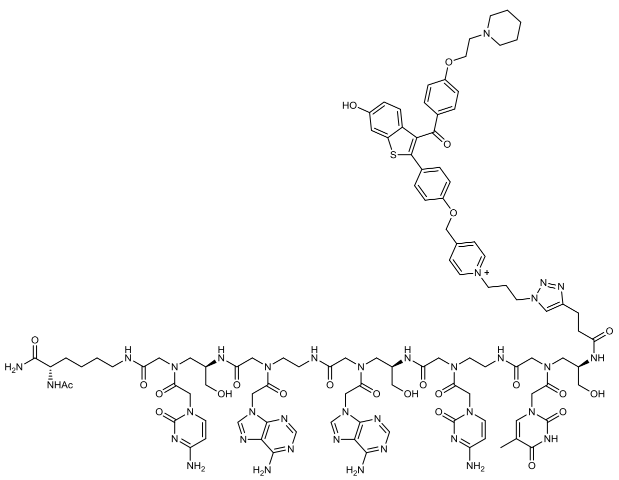

Sequence: Lys-C*AA*CT*-Pyridinium-Ibrutinib (PNA-PyIbr) **7**

Chemical Formula: $C_{104}H_{128}N_{41}O_{24}^{+}$, Exact Mass: 2335.01, **LC-MS (ESI^{+})** RT= 1.77 min. *m/z* found: 1168.17 $[M+H]^{2+}$, 779.33 $[M+2H]^{3+}$; **MALDI-TOF** calculated *m/z*: 1852.834 (100%), *m/z* found: 1853.797 $[M+H\text{-Ibrutinib}]^{+}$.

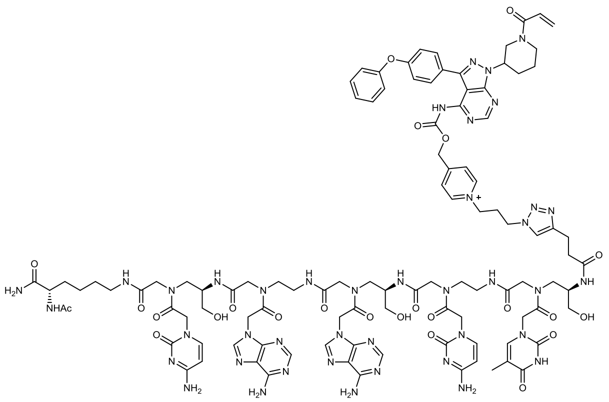

Sequence: Lys-C*AA*CT*-Pyridinium-Duocarmycin (PNA-PyDuo) **8**

Chemical Formula: $C_{106}H_{125}ClN_{37}O_{29}S_2^+$, Exact Mass: 2478.85, **LC-MS (ESI$^+$)** RT= 1.99 min. *m/z* found: 1240.92 $[M+H]^{2+}$, 827.58 $[M+2H]^{3+}$; **MALDI-TOF** calculated *m/z*: 2074.764 (100%), *m/z* found: 2073.490 [M-Duocarmycin]$^+$.

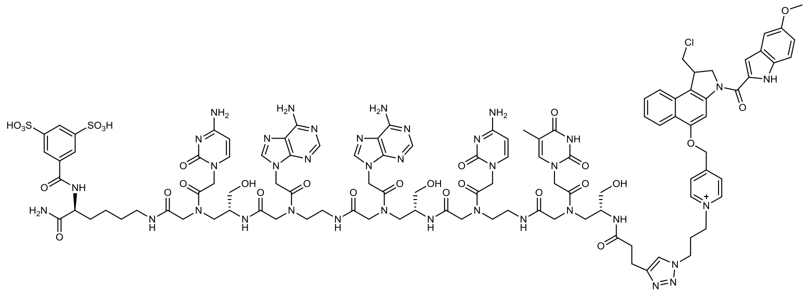

Sequence: Lys-C*AA*CT*-SecondaryPyridinium-Rhodamine (PNA-Py^{2}Rho) **10**

Chemical Formula: $C_{103}H_{125}N_{38}O_{26}^+$, Exact Mass: 2309.96, **LC-MS (ESI$^+$)** RT= 1.60 min. *m/z* found: 1156.00 $[M+H]^{2+}$, 771.17 $[M+2H]^{3+}$, 578.67 $[M+3H]^{4+}$; **MALDI-TOF** calculated *m/z*: 1880.866 (100%), *m/z* found: 1880.764 [M-Rhodamine]$^+$.

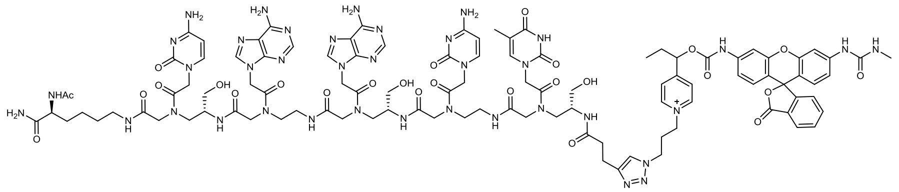

Sequence: Lys-C*AA*CT*-Lutidinium-Rhodamine (PNA-LutRho) **11**

Chemical Formula: $C_{103}H_{125}N_{38}O_{26}^+$, Exact Mass: 2309.96, **LC-MS (ESI$^+$)** RT= 1.63 min. *m/z* found: 1156.00 $[M+H]^{2+}$, 771.17 $[M+2H]^{3+}$, 578.75 $[M+3H]^{4+}$; **MALDI-TOF** calculated *m/z*: 1880.866 (100%), *m/z* found: 1880.747 [M-Rhodamine]$^+$.

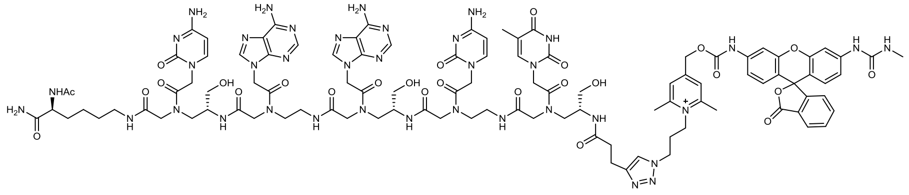

Sequence: Lys-C*AA*CT*-SecondaryPyridinium-Duocarmycin (PNA-Py^{2}Duo) **12**

Chemical Formula: $C_{108}H_{129}ClN_{37}O_{29}S_2^+$, Exact Mass: 2506.88, **LC-MS (ESI$^+$)** RT= 2.10 min. *m/z* found: 1254.75 $[M+H]^{2+}$, 837.08 $[M+2H]^{3+}$; **MALDI-TOF** calculated *m/z*: 2102.795 (100%), *m/z* found: 2102.716 $[M\text{-}Duocarmycin]^+$.

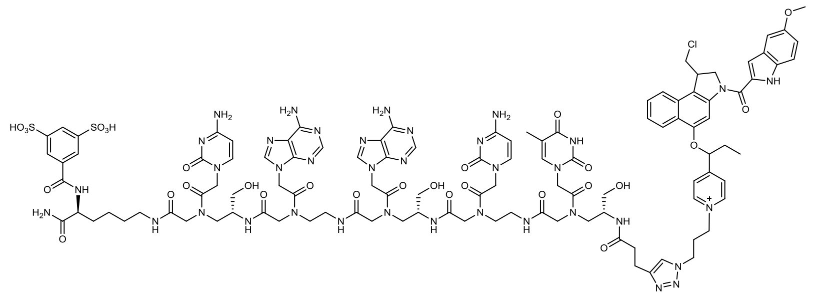

Synthesis of small molecules.

Synthesis of Pyridinium-Rhodamines

Methylurea-Rhodamine was prepared as previously described[16]

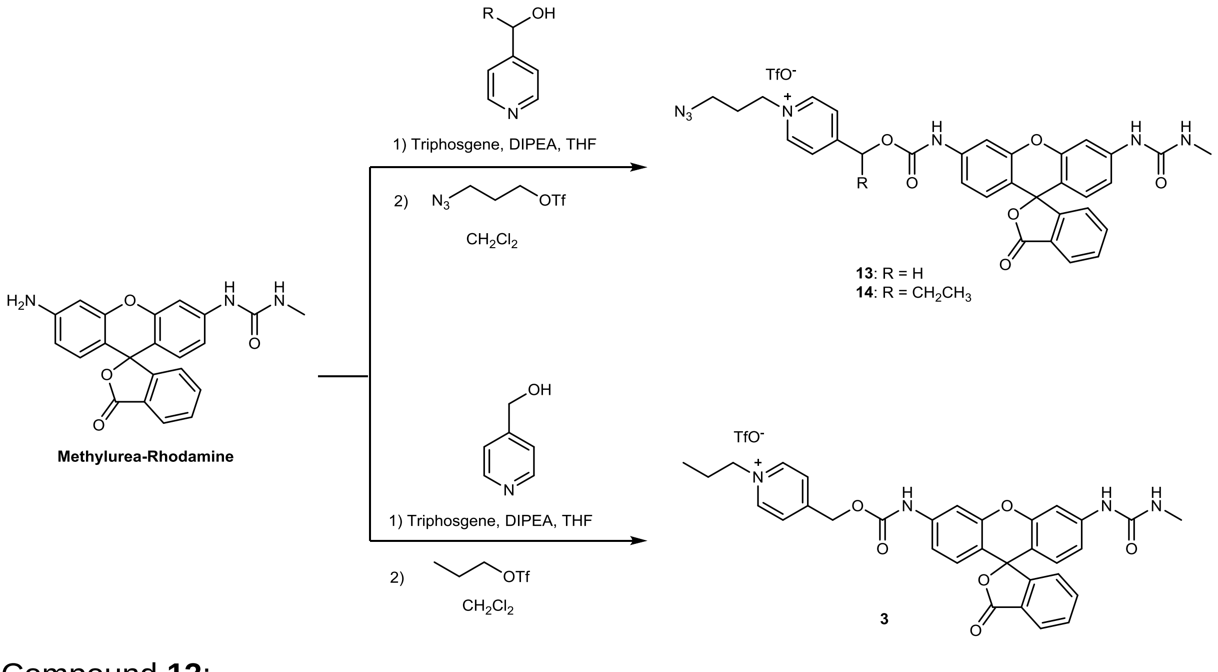

Compound **13**:

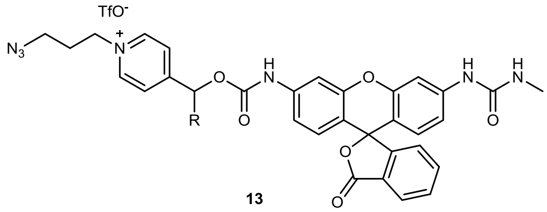

13

To a solution of methylurea-rhodamine (50 mg, 0.13 mmol, 1 equiv) in dry THF (3 mL) under nitrogen at 0 °C was added triphosgene (19.3 mg, 0.07 mmol, 0.5 equiv), followed by slow

addition of DIPEA (70 µL, 0.39 mmol, 3 equiv) dropwise. Upon addition of DIPEA the solution became colorless and the formation of the isocyanate was monitored via LC-MS by taking small aliquots of the reaction mixture and quenching in MeOH before injection into the LC-MS. 4-pyridine-methanol (43 mg, 0.39 mmol, 3 equiv) was then added and the reaction was heated at 60 °C overnight under nitrogen. The reaction mixture was then concentrated, dissolved in DMSO, loaded and purified using reverse phase Biotage Isolera ONE equipped with a Biotage SNAP Cartridge KP-C18-HS. The desired fractions were combined and concentrated to yield pyridine-rhodamine-methylurea. The solid was dissolved in dichloromethane, cooled to -78°C, azidopropyl trifluoromethanesulfonate (1.1 equiv) was added and the reaction was allowed to warm up to room temperature slowly. The reaction was monitored via LC-MS and once all the starting material had been consumed the reaction mixture was concentrated, dissolved in H_2O and DMSO and purified via RP-HPLC to give the desired N_3PyRho (**13**) compound as a dark red solid. **^{1}H NMR** (500 MHz, Acetone-*d6)* δ 9.18 (d, *J* = 6.3 Hz, 2H), 8.26 (d, *J* = 6.2 Hz, 2H), 7.98 (d, *J* = 7.5 Hz, 1H), 7.82 (d, *J* = 2.0 Hz, 1 H), 7.79 (td, *J* = 1.2 Hz, 7.5 Hz, 1H), 7.72 (td, *J* = 1.0 Hz, 7.6 Hz), 7.66 (d, *J* = 2.2 Hz, 1H), 7.29 (d, *J* = 7.7 Hz, 1H), 7.22 (dd, *J* = 2.2 Hz, 8.8 Hz, 1H), 7.13 (dd, *J* = 2.1 Hz, 8.8 Hz, 1H), 6.74 (d, *J* = 8.7 Hz, 1H), 6.62 (d, *J* = 8.6 Hz) 5.53 (s, 2H), 4.94 (t, *J* = 7.3 Hz, 2H), 3.61 (t, *J* = 6.4 Hz, 2H), 2.72 (s, 3H), 2.43 (m, 2H). **^{13}C NMR** (126 MHz, Acetone-*d6*)δ 169.66, 158.82, 154.04, 152.80, 152.58, 145.79, 136.18, 130.84, 129.48, 128.86, 127.66, 126.29, 125.46, 125.00, 115.26, 115.18, 115.09, 115.01, 114.86, 111.81, 106.80, 105.52, 105.46, 83.47, 64.54, 60.00, 48.77, 31.06, 26.51. Exact mass: 606.21 **LC-MS (ESI$^+$)**. RT= 1.95 min. *m/z* found: 606.33 $[M]^+$, 303.75 $[M]^{2+}$.

Compound **3**:

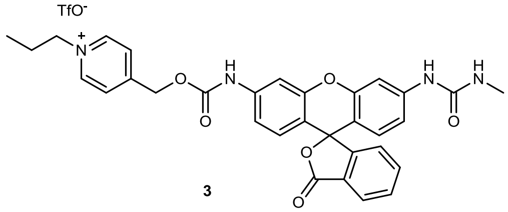

3

PrPyRho (**3**) was synthesized using the same method as for N_3PyRho, using propyl trifluoromethansulfonate instead of azidopropyl trifluoromethansulfonate. **^{1}H NMR** (500 MHz, MeOD) δ 8.96 (d, *J* = 6.8 Hz, 2H), 8.11 (d, *J* = 6.3 Hz, 2H), 8.02 (dt, *J* = 7.6, 1.0 Hz, 1H), 7.78 (td, *J* = 7.5, 1.2 Hz, 1H), 7.72 (td, *J* = 7.5, 1.0 Hz, 1H), 7.67 (d, *J* = 2.2 Hz, 1H), 7.62 (d, *J* = 2.1 Hz, 1H), 7.24 – 7.19 (m, 1H), 7.10 (dd, *J* = 8.7, 2.2 Hz, 1H), 6.89 (dd, *J* = 8.7, 2.2 Hz, 1H), 6.71 (d, *J* = 8.7 Hz, 1H), 6.64 (d, *J* = 8.6 Hz, 1H), 5.54 (s, 2H), 4.58 (t, J = 7.4 Hz, 2H), 2.78 (s, 3H), 2.05 (h, *J* = 7.4 Hz, 2H), 1.02 (t, *J* = 7.4 Hz, 3H). **^{13}C NMR** (126 MHz, MeOD) δ 171.37, 159.28, 158.46, 154.41, 154.32, 153.30, 153.17, 145.75, 143.89, 142.29, 136.73, 131.28, 129.61, 129.30, 127.85, 126.38, 125.91, 125.16, 115.62, 115.53, 114.89, 113.12, 107.14, 106.71, 64.88, 64.00, 26.78, 25.72, 10.70. Exact mass: 565.21. **LC-MS (ESI$^+$)** RT= 1.96 min. *m/z* found: 565.40 $[M]^+$, 283.36 $[M+H]^{2+}$. **HRMS** : 565.20441 (± 6.70 ppm).

Compound **14**:

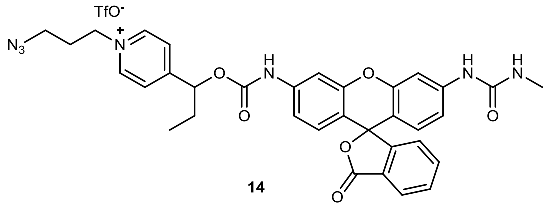

14

N_3Py^2Rho (**14**) was synthesized using the same method as for N_3PyRho, using 1-(pyridin-4-yl)propan-1-ol instead of 4-pyridine methanol.

^{1}H NMR (400 MHz, MeOD) δ 8.87 (d, *J* = 6.5 Hz, 2H), 8.03 (d, *J* = 6.4 Hz, 2H), 7.92 (d, *J* = 7.6 Hz, 1H), 7.67 (t, *J* = 7.4 Hz, 1H), 7.64 – 7.59 (m, 1H), 7.57 – 7.55 (m, 1H), 7.49 (d, *J* = 2.0 Hz, 0.5H), 7.42 (d, *J* = 2.1 Hz, 0.5H), 7.10 (dd, *J* = 7.6, 3.4 Hz, 1H), 6.99 (dd, *J* = 8.7, 2.2 Hz, 0.5H), 6.93 (dd, *J* = 8.7, 2.2 Hz, 0.5H), 6.78 (dd, *J* = 8.6, 2.2 Hz, 1H), 6.58 (d, *J* = 8.7 Hz, 1H), 6.53 (d, *J* = 8.7 Hz, 1H), 5.81 – 5.76 (m, 1H), 4.60 (t, *J* = 7.2 Hz, 2H), 3.41 (t, *J* = 6.3 Hz, 2H), 2.24 – 2.13 (m, 2H), 1.98 – 1.86 (m, 2H), 0.97 (t, *J* = 7.3 Hz, 3H). **^{13}C NMR** (126 MHz, MeOD) δ 163.18, 158.46, 154.40, 153.17, 146.23, 143.88, 142.28, 136.71, 131.28, 130.80, 129.58, 129.29, 127.85, 126.31, 125.90, 125.16, 115.60, 113.11, 107.08, 106.69, 76.92, 60.21, 31.19, 30.03, 28.13, 26.77, 9.79.. Exact mass: 634.24. **LC-MS (ESI$^+$)** RT= 2.07 min. *m/z* found: 634.33 $[M]^+$, 317.75 $[M+H]^{2+}$. **HRMS** : 634.23676 (± 6.52 ppm).

Compound **15**:

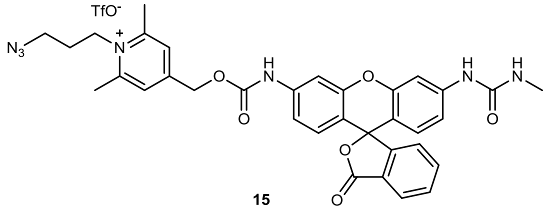

15

$N_3LutRho$ (**15**) was synthesized using the same method as for N_3PyRho, using (2,6-dimethylpyridin-4-yl)methanol instead of 4-pyridine methanol. The crude mixture was used for the click reaction with PNA without further purification.
Exact mass: 634.24. **LC-MS (ESI$^+$)** RT= 2.11 min. *m/z* found: 634.27 $[M]^+$, 317.80 $[M+H]^{2+}$.

Synthesis of Pyridinium-Raloxifene derivative (N_3PyRal) 16

Raloxifene was synthesized according to a previously reported procedure.[7]

Compounds **16a,b**:

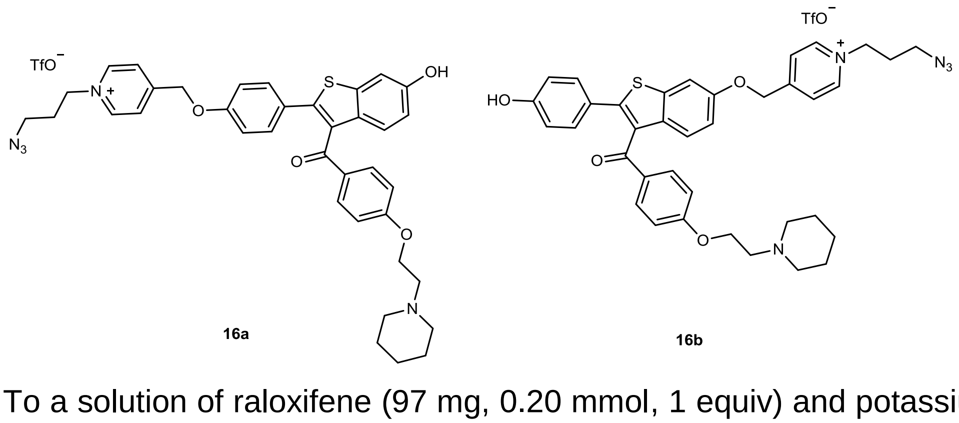

To a solution of raloxifene (97 mg, 0.20 mmol, 1 equiv) and potassium carbonate (90 mg, 0.65 mmol, 3.2 equiv) in dry DMF (3 mL) was added , 4-(bromomethyl)pyridine hydrobromide[2] (66 mg, 0.26 mmol, 1.3 equiv) in DMF (2.5 mL) dropwise over 2h. The reaction mixture was poured into sodium bicarbonate solution and extracted with EtOAc (3x). The organic phase was then washed with water, followed by brine, dried over Na_2SO_4, filtered and concentrated *in vacuo*. The residue was loaded onto a short silica gel column and eluted with DCM:MeOH (99:1→90:10) to obtain a mixture of the two isomers. The obtained residue (39 mg, 0.07 mmol,

1 equiv) was then dissolved in DCM and cooled to -40 °C, followed by the addition of 3-azidopropyl trifluoromethanesulfonate[3] (0.07 mmol, 1 equiv). The reaction was then allowed to reach room temperature slowly and was monitored by LC-MS until all starting material had been consumed. The crude mixture was used for the click reaction with PNA without further purification. Exact mass: 648.26, **LC-MS (ESI$^+$)** RT = 1.86 min. *m/z* found: 648.18 $[M]^+$, 324.79 $[M+H]^{2+}$.

Synthesis of Pyridinium-Ibrutinib derivative (N_3PyIbr) 17

Ibrutinib (racemic) was synthesized according to a previously reported procedure.[17, 18]

Compound **17**:

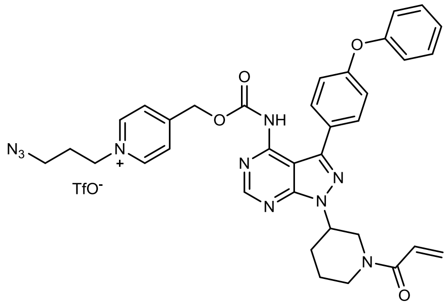

17

NaH (60% dispersion in mineral oil) (2 mg, 0.09 mmol, 4 equiv) was dissolved in DMF (2.3 mL) and stirred for 15 minutes at room temperature. Ibrutinib (10 mg, 0.023 mmol, 1 equiv) was added to the previous suspension at 0 °C and the mixture was stirred for 1 hour before the addition of 4-nitrophenyl(pyridin-4-ylmethyl)carbonate (13 mg, 0.046 mmol, 2 equiv). The mixture was allowed to reach room temperature and stirred overnight. The mixture was poured into 50 mL of water and extracted with EtOAc (3 x 15 mL); the organic layers were combined, washed with brine (20 mL), dried over Na_2SO_4 and concentrated. The crude product (10 mg) was used in the next step without further purifications.

The crude residue was dissolved in CH_2Cl_2 (1.7 mL) and cooled to -78 °C. 3-azidopropyl trifluoromethanesulfonate (5 mg, 0.0174 mmol, 1 equiv) was added dropwise; the mixture was allowed to reach room temperature and stirred for 3 hours. The solution was then concentrated and purified by RP-HPLC. **17** was obtained as a pale yellow solid. **^{1}H NMR** (500 MHz, Chloroform-*d*, rotamers) δ 9.05 – 8.98 (m, 2H), 8.48 – 8.39 (m, 1H), 8.02 – 7.89 (m, 3H), 7.44 – 7.32 (m, 2H), 7.20 – 7.02 (m, 6H), 6.68 – 6.49 (m, 1H), 6.35 – 6.23 (m, 1H), 5.78 – 5.62 (m, 1H), 5.36 (s, 2H), 4.98 – 4.87 (m, 1H), 4.82 (t, *J* = 7.1 Hz, 2H), 4.63 – 4.54 (m, 0.5H), 4.25 – 4.15 (m, 0.5H), 4.09 – 3.97 (m, 1H), 3.83 – 3.73 (m, 0.5H), 3.50 (t, *J* = 5.9 Hz, 2H), 3.47 – 3.37 (m, 0.5H), 3.30 – 3.19 (m, 1H), 2.99 – 2.87 (m, 0.5H), 2.46 – 2.34 (m, 1H), 2.34 – 2.22 (m, 2H), 2.07 – 1.99 (m, 1H), 1.80 – 1.65 (m, 1H), 0.91 – 0.78 (m, 1H). **^{13}C NMR** (126 MHz, $CDCl_3$) δ 165.92, 161.43, 158.62, 157.51, 156.66, 153.92, 144.95, 130.80, 130.11, 130.08, 128.46, 127.60, 127.15, 125.95, 125.77, 124.09, 120.07, 119.49, 119.38, 118.81, 115.17, 64.60, 59.47, 54.11, 53.01, 50.12, 47.78, 46.25, 46.07, 42.33, 30.48, 30.30, 25.32, 23.96, 20.30. Exact mass: 659.2837, **LC-MS (ESI$^+$)** RT= 2.36 min. *m/z* found: 659.26 $[M]^+$. **HRMS** : 659.27936 (± 6.58 ppm).

Synthesis of Ibrutinib-Cy3 conjugate (Ibr-Cy3) 9

Intermediate **18** was synthesized as previously described[17,19]

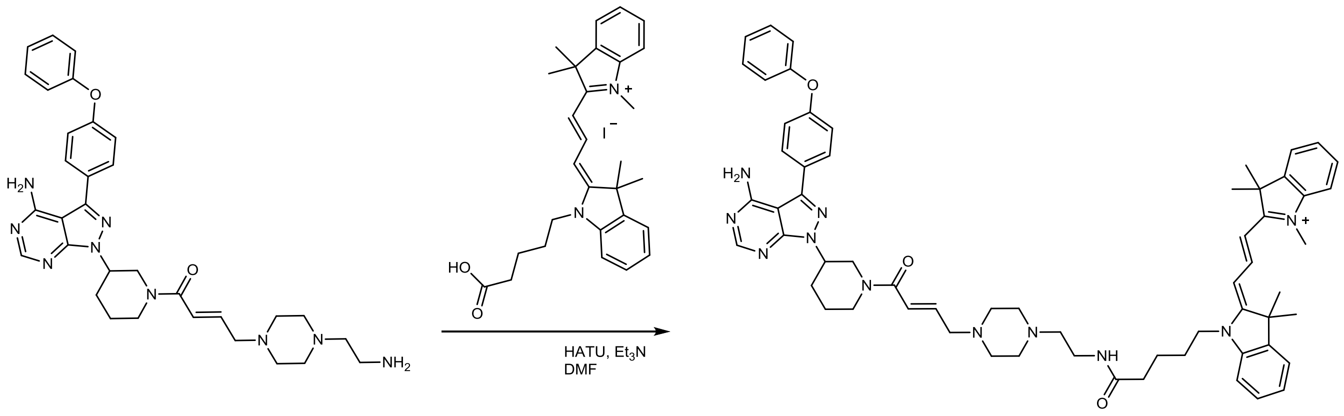

18 9

Compound **9**:

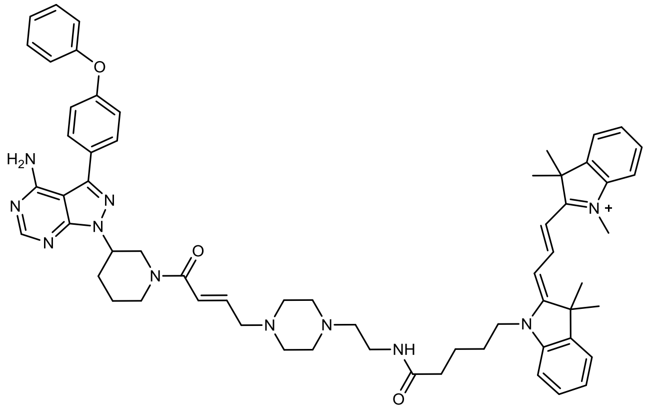

9

To a solution of Cy3-COOH (7.4 mg, 0.013 mmol, 1 equiv) in DMF (1 mL) was added HATU (4.7 mg, 0.012 mmol, 0.92 equiv) and Et_3N (7 µl, 0.05 mmol, 3.8 equiv) and the reaction was stirred at room temperature for 10 minutes after which **18** (5.8 mg, 0.01 mmol, 0.76 equiv) was added in DMF and the reaction was stirred at room temperature for 1 hour. The reaction mixture was then diluted in water and purified by RP-HPLC giving Ibr-Cy3 (1.1 mg) as a dark red solid. Isomers: **^{1}H NMR** (400 MHz, Chloroform-d) δ 11.82 (s, 0.3H), 11.69 (s, 0.15H), 8.47 – 8.20 (m, 2H), 7.58 (d, J = 8.2 Hz, 1.4H), 7.48 – 7.34 (m, 5H), 7.33 – 7.28 (m, 1.77H), 7.24 – 7.13 (m, 2.85H), 7.09 (d, J = 8.0 Hz, 1H), 6.93 – 6.73 (m, 1H), 6.59 – 6.37 (m, 1H), 6.31 – 6.21 (m, 0.5H), 4.94 – 4.77 (m, 0.85H), 4.57 (d, J = 12.2 Hz, 0.25H), 4.20 (s, 0.5H), 4.13 – 3.97 (m, 1.85H), 3.86 – 3.48 (m, 10.85H), 3.19 (d, J = 81.9 Hz, 22.20H), 2.49 – 2.21 (m, 3.75H), 2.12 – 1.99 (m, 0.8H), 1.92 – 1.72 (m, 9H), 0.91 – 0.76 (m, 2H).**^{13}C NMR** (126 MHz, $CDCl_3$) δ 174.73, 174.05, 159.86, 155.84, 153.79, 151.66, 150.68, 147.03, 146.00, 142.64, 141.83, 140.49, 140.44, 132.18, 130.27, 129.96, 129.88, 129.29, 129.16, 125.84, 125.29, 124.70, 124.64, 122.27, 122.19, 120.04, 119.37, 111.23, 111.06, 103.92, 103.51, 97.33, 97.17, 77.37, 56.83, 54.13, 53.50, 49.35, 49.25, 48.49, 46.21, 44.22, 42.35, 35.42, 34.03, 33.95, 30.27, 29.85, 28.27, 28.19, 26.90, 25.19, 23.77, 22.54. Exact mass: 1006.5814, **LC-MS (ESI$^+$)** RT = 2.37 min. *m/z* found: 1006.57 $[M]^+$, 504.00 $[M+H]^{2+}$.336.33 $[M+2H]^{3+}$. **MALDI-TOF** calculated *m/z*: 1006.581 (100%), *m/z* found: 1006.711 $[M]^+$. **HRMS** : 1006.57654 (± 4.86 ppm).

Synthesis of Duocarmycin derivative (Duo-OMe) 4

Derivative **19** was synthesized according to a previously reported procedure.[20]

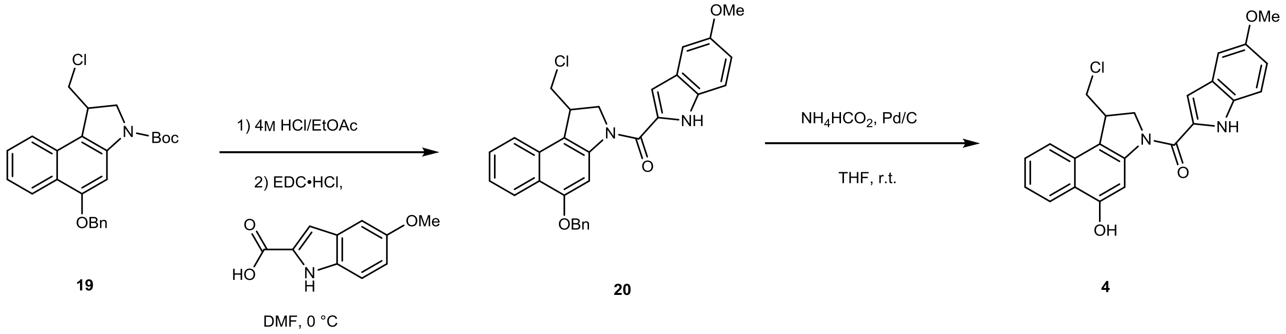

Compound **20**:

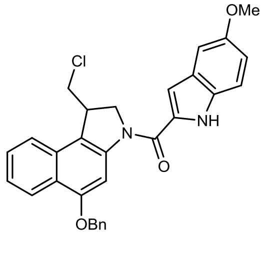

19 (5 mg, 0.012 mmol, 1 equiv) was treated with 4 M HCl/EtOAc (0.3 mL) at room temperature and stirred for 1 hour. Then the mixture was concentrated and dried under vacuum. The residue was dissolved in DMF (0.5 mL) and cooled to 0 °C. 5-methoxy-1H-indole-2-carboxylic acid (2.5 mg, 0.012 mmol, 1 equiv) and EDC·HCl (6.8 mg, 0.035 mmol, 3 equiv) were added and the mixture was stirred at 0 °C for 2 hours. Then the mixture was diluted with $NaHCO_3$ saturated solution (10 mL) and extracted with EtOAc (3 x 5 mL). The organic layers were combined, washed with brine (2 x 5 mL), dried over Na_2SO_4 and concentrated. The crude was purified by preparative TLC on silica gel (Hexane/EtOAc 2:1) affording **20** as yellow oil (4 mg, 65%). **^{1}H NMR** (400 MHz, Chloroform-*d*) δ 9.33 (s, 1H), 8.36 (dd, *J* = 8.3, 1.0 Hz, 1H), 8.19 (s, 1H), 7.73 (dd, *J* = 8.3, 0.9 Hz, 1H), 7.62 – 7.52 (m, 3H), 7.48 – 7.32 (m, 5H), 7.15 (d, *J* = 2.3 Hz, 1H), 7.07 (dd, *J* = 2.3, 0.9 Hz, 1H), 7.03 (dd, *J* = 8.9, 2.4 Hz, 1H), 5.32 (q, *J* = 11.5 Hz, 2H), 4.84 (dd, *J* = 10.8, 1.9 Hz, 1H), 4.70 (dd, *J* = 10.8, 8.3 Hz, 1H), 4.16 (ddt, *J* = 9.9, 8.3, 2.8 Hz, 1H), 4.05 – 3.95 (m, 1H), 3.88 (s, 3H), 3.55 – 3.43 (m, 1H). **^{13}C NMR** (126 MHz, $CDCl_3$) δ 160.75, 156.00, 154.91, 142.25, 136.86, 131.35, 130.70, 129.93, 128.76, 128.51, 128.21, 128.02, 127.76, 124.27, 123.91, 123.85, 122.30, 117.04, 116.51, 112.81, 106.28, 102.66, 98.47, 77.42, 77.36, 77.16, 76.91, 70.56, 55.88, 55.39, 46.20, 43.38. Exact mass: 496.16 **MS (ESI$^+$)** *m/z* found: 497.33 $[M+H]^+$.

Compound **4**:

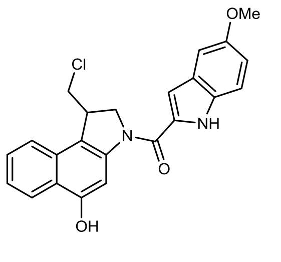

4

20 (4 mg, 0.008 mmol, 1 equiv) was dissolved in THF (170 µL). Pd/C 10% (2.5 mg) was added under stirring and a 25% (w/w) aqueous solution of NH_4HCO_2 (15 µL, 8 equiv) was added dropwise. After stirring for 2 hours at room temperature, the mixture was filtered through Celite and washed with Et_2O. The filtrates were dried over Na_2SO_4 and concentrated. The residue was purified by RP-HPLC affording **Duo-OMe** (**4**) as a white foam (1.5 mg, 46%). **^{1}H NMR** (500 MHz, DMSO-d_6) δ 11.60 (d, J = 2.2 Hz, 1H), 10.44 (s, 1H), 8.11 (dd, J = 1.3 Hz, 1H), 7.97 (s, 1H), 7.85 (d, J = 8.4 Hz, 1H), 7.52 (ddd, J = 8.4, 6.8, 1.3 Hz, 1H), 7.42 – 7.31 (m, 2H), 7.16 (d, J = 2.4 Hz, 1H), 7.10 (d, J = 2.2 Hz, 1H), 6.91 (dd, J = 8.9, 2.4 Hz, 1H), 4.79 (dd, J = 10.9, 8.9 Hz, 1H), 4.55 (dd, J = 10.9, 2.1 Hz, 1H), 4.26 – 4.18 (m, 1H), 4.02 (dd, J = 11.1, 3.2 Hz, 1H), 3.85 (dd, J = 11.1, 7.5 Hz, 1H), 3.78 (s, 3H). **^{13}C NMR** (126 MHz, DMSO) δ 160.14, 154.13, 153.86, 142.31, 131.57, 131.03, 129.91, 127.50, 127.34, 123.16, 123.11, 122.79, 122.13, 115.46, 114.96, 113.18, 105.30, 102.09, 100.35, 55.28, 55.03, 47.62, 41.23. Exact mass: 406.11, **LC-MS (ESI$^+$)** RT= 2.78 min. *m/z* found: 407.19 $[M+H]^+$.

Synthesis of Secondary-Pyridinium-Duocarmycin derivative (N_3Py^2Duo-OMe) 24

Compound **21** was synthesized according to a previously reported procedure.[20]

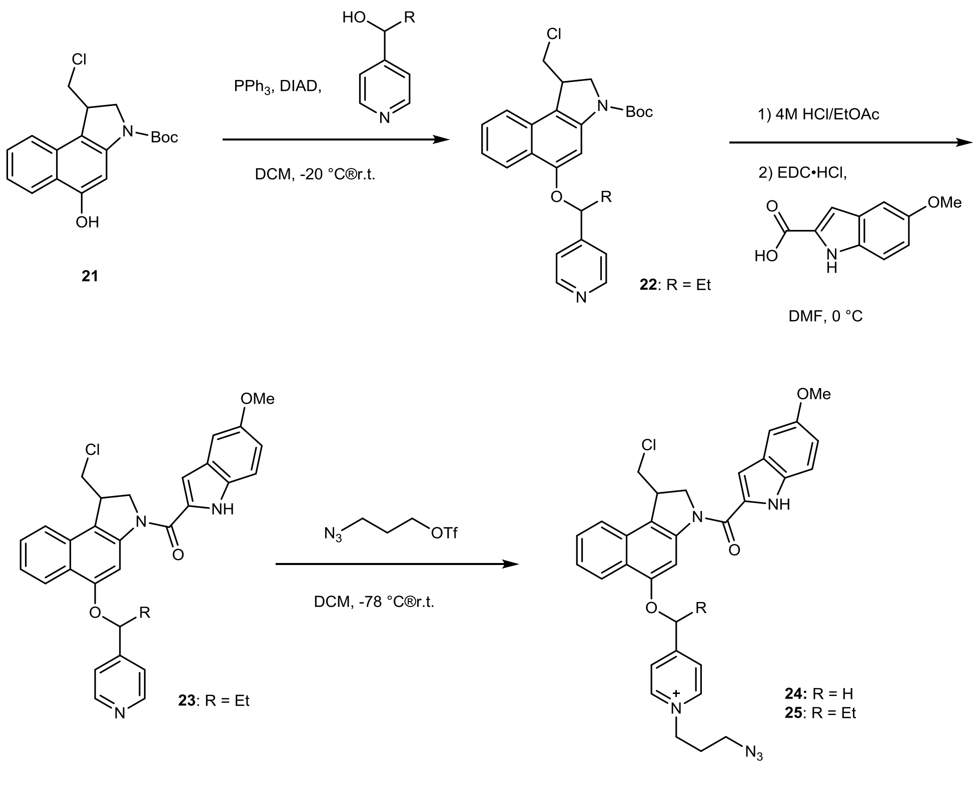

Compound **22**:

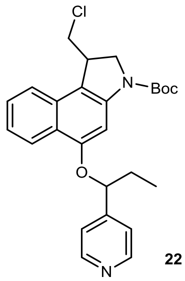

22

21 (70 mg, 0.21 mmol, 1 equiv), 1-(pyridin-4-yl)propan-1-ol (58 mg, 0.42 mmol, 2 equiv) and PPh_3 (125 mg, 0.46 mmol, 2.2 equiv) were dissolved in CH_2Cl_2 (4 mL) in and the mixture was cooled to -20 °C. After the dropwise addition of DIAD (83 µL, 0.42 mmol, 2 equiv), the mixture was allowed to reach room temperature and stirred for 2 hours. Then the mixture was concentrated over silica and purified by flash chromatography on silica gel (Hexane/EtOAc 5:1) affording **22** as a pale yellow solid (58 mg, 61%). **^{1}H NMR** (400 MHz, Chloroform-*d*) δ 8.58 – 8.52 (m, 2H), 8.39 (d, *J* = 8.4 Hz, 1H), 7.67 – 7.60 (m, 1H), 7.56 – 7.49 (m, 1H), 7.44 – 7.36 (m, 1H), 7.33 (s, 1H), 5.41 (s, 1H), 4.26 – 4.14 (m, 1H), 4.11 – 3.98 (m, 1H), 3.97 – 3.82 (m, 2H), 3.45 – 3.29 (m, 1H), 2.16 – 1.99 (m, 2H), 1.53 (s, 9H), 1.07 (td, *J* = 7.4, 5.4 Hz, 1H). **^{13}C NMR** (126 MHz, $CDCl_3$) δ 154.62, 152.50, 150.08, 130.51, 127.86, 123.47, 123.46, 122.69, 122.07, 121.33, 98.00, 97.93, 79.55, 53.15, 46.60, 46.47, 41.79, 30.85, 28.59, 28.58, 28.33, 9.96, 9.85. Exact mass: 452.19 **MS (ESI$^+$)** *m/z* found: 453.24 $[M+H]^+$.

Compound **23**:

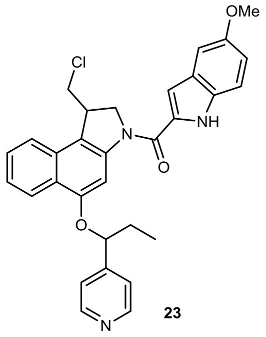

23

22 (15 mg, 0.033 mmol, 1 equiv) was treated with 4 M HCl/EtOAc (0.5 mL) at room temperature and stirred for 1 hour. Then the mixture was concentrated and dried under vacuum. The residue was dissolved in DMF (1.0 mL) and cooled to 0 °C. 5-methoxy-1H-indole-2-carboxylic acid (6.3 mg, 0.033 mmol, 1 equiv) and EDC·HCl (20 mg, 0.099 mmol, 3 equiv) were added and the mixture was stirred at 0 °C for 2 hours. Then the mixture was diluted with $NaHCO_3$ saturated solution (10 mL) and extracted with EtOAc (3 x 5 mL). The organic layers were combined, washed with brine (2 x 5 mL), dried over Na_2SO_4 and concentrated. The crude was purified by flash chromatography on silica gel (Hexane/EtOAc from 6:4 to 2:8) affording **23** as a white solid (13 mg, 75%). **^{1}H NMR** (500 MHz, Chloroform-*d*) δ 9.22 (s, 1H), 9.18 (s, 1H), 8.66 – 8.56 (m, 1H), 8.52 (d, *J* = 5.3 Hz, 1H), 8.49 – 8.39 (m, 1H), 7.96 (s, 1H), 7.74 – 7.70 (m, 1H), 7.61 – 7.56 (m, 1H), 7.51 – 7.45 (m, 1H), 7.39 (d, *J* = 5.2 Hz, 1H), 7.35 (d, *J* = 8.9 Hz, 1H), 7.31 (s, 1H), 7.13 – 7.10 (m, 1H), 7.05 – 6.97 (m, 2H), 5.42 – 5.37 (m, 1H), 4.79 – 4.72 (m, 1H), 4.68 – 4.57 (m, 1H), 4.16 – 4.04 (m, 1H), 3.99 – 3.92 (m, 1H), 3.87 (d, *J* = 1.4 Hz, 3H), 3.48 – 3.37 (m, 1H), 2.19 – 2.12 (m, 1H), 2.09 – 2.02 (m, 1H), 1.14 – 1.05 (m, 3H). **^{13}C NMR** (126 MHz, $CDCl_3$) δ 160.65, 160.62, 154.89, 154.51, 154.48,

150.45, 150.35, 150.28, 150.26, 142.13, 131.30, 130.66, 130.57, 130.03, 128.45, 128.07, 128.04, 124.40, 124.34, 123.87, 123.60, 122.46, 121.33, 121.18, 117.01, 116.47, 112.76, 106.27, 106.14, 102.64, 102.62, 99.87, 99.81, 80.06, 55.88, 55.38, 55.22, 46.18, 46.04, 43.36, 43.23, 31.06, 30.82, 10.09, 10.00. Exact mass: 525.18 **MS (ESI$^+$)** *m/z* found: 526.28 $[M+H]^+$.

Compound **24**:

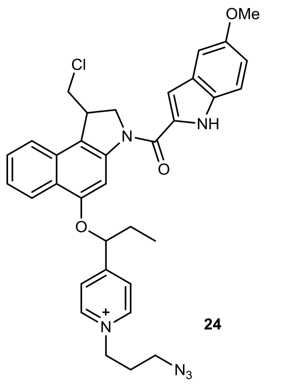

24

23 (5 mg, 0.01 mmol, 1 equiv) was dissolved in CH_2Cl_2 (300 µL) and cooled to -78 °C. Then 3-azidopropyl trifluoromethanesulfonate[3] (2.3 mg, 0.01 mmol, 1 equiv) was added dropwise. The mixture was stirred at -78 °C for 10 minutes; then it was allowed to reach room temperature and stirred for additional 2 hours. The mixture was concentrated and the residue was purified by RP-HPLC affording N_3Duo-OMe (**24**) as a pale yellow foam (2.55 mg, 35%). Pair of diastereomers (60:40 ratio): **^{1}H NMR** (400 MHz, Chloroform-*d*) δ 10.42 (s, 0.3H), 10.21 (s, 0.5H), 9.12 (d, *J* = 5.9 Hz, 2H), 8.42 (dd, *J* = 8.3, 3.0 Hz, 1H), 8.05 (d, *J* = 5.7 Hz, 1H), 7.90 (d, *J* = 5.7 Hz, 1H), 7.80 – 7.73 (m, 1H), 7.72 – 7.61 (m, 2H), 7.55 (ddd, *J* = 8.2, 6.8, 1.2 Hz, 1H), 7.38 (d, *J* = 8.9 Hz, 0.6H), 7.34 (d, *J* = 9.0 Hz, 0.4H), 7.13 (d, *J* = 2.4 Hz, 0.6H), 7.09 (d, *J* = 2.4 Hz, 0.4H), 7.01 – 6.85 (m, 2H), 5.61 – 5.51 (m, 1H), 4.92 – 4.74 (m, 2H), 4.74 – 4.67 (m, 1H), 4.65 – 4.53 (m, 1H), 4.21 – 4.12 (m, 0.6H), 4.10 – 3.97 (m, 0.8H), 3.95 – 3.87 (m, 0.6H), 3.88 (s, 1.8H), 3.85 (s, 1.2H), 3.57 – 3.49 (m, 0.6H), 3.47 – 3.33 (m, 2.4H), 2.35 – 2.21 (m, 2H), 2.22 – 2.09 (m, 2H), 1.25 – 1.15 (m, 3H). **^{13}C NMR** (126 MHz, $CDCl_3$) δ 162.35, 162.24, 161.09, 154.79, 154.69, 153.12, 152.95, 145.37, 141.81, 141.65, 131.99, 131.82, 130.40, 130.26, 130.19, 130.09, 128.46, 128.39, 128.14, 127.96, 125.81, 125.50, 124.89, 124.80, 123.26, 123.20, 123.14, 123.11, 122.68, 117.74, 117.63, 116.92, 116.76, 113.43, 113.32, 106.36, 106.19, 102.28, 102.14, 100.79, 100.43, 79.32, 59.23, 59.17, 55.82, 55.80, 55.40, 55.10, 47.86, 47.79, 46.46, 45.89, 43.20, 42.93, 30.75, 30.71, 30.40, 30.34, 10.06, 10.02. Exact mass: 609.24, **LC-MS (ESI$^+$)** RT= 2.68 min. *m/z* found: 609.47 $[M]^+$. **HRMS** : 609.23328 (± 6.92 ppm).

Synthesis of Pyridinium-Duocarmycin derivative (N_3PyDuo-OMe) 25

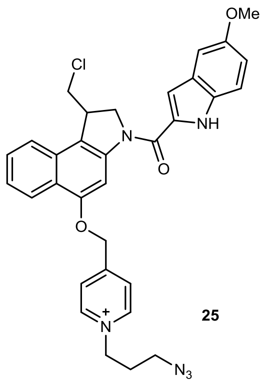

25

Primary pyridinium derivatives were synthesized according to the synthetic strategy used for the secondary Py derivatives and using 4-pyridinemethanol as pyridine source.

^{1}H NMR (400 MHz, DMSO-d_6) δ 11.56 (d, J = 2.2 Hz, 1H), 9.16 – 9.07 (m, 2H), 8.44 – 8.34 (m, 3H), 8.12 (s, 1H), 7.99 (d, J = 8.3 Hz, 1H), 7.65 (ddd, J = 8.3, 6.8, 1.3 Hz, 1H), 7.52 (ddd, J = 8.3, 6.8, 1.1 Hz, 1H), 7.41 (d, J = 8.9 Hz, 1H), 7.19 – 7.11 (m, 2H), 6.93 (dd, J = 8.9, 2.5 Hz, 1H), 5.71 (s, 2H), 4.85 (dd, J = 11.0, 9.2 Hz, 1H), 4.70 (t, J = 7.2 Hz, 2H), 4.60 (dd, J = 11.0, 2.3 Hz, 1H), 4.39 – 4.29 (m, 1H), 4.07 (dd, J = 11.1, 3.2 Hz, 1H), 3.95 (dd, J = 11.1, 6.6 Hz, 1H), 3.79 (s, 3H), 3.50 (t, J = 6.5 Hz, 2H), 2.23 (p, J = 6.8 Hz, 2H). **^{13}C NMR** (126 MHz, DMSO) δ 160.33, 156.73, 153.92, 153.37, 144.89, 142.20, 131.64, 130.77, 129.75, 127.97, 127.52, 125.24, 124.31, 123.18, 122.77, 122.32, 117.70, 115.64, 113.22, 105.57, 102.10, 98.72, 67.40, 58.21, 55.29, 55.13, 47.75, 47.59, 41.07, 29.70. Exact mass: 581.20, **LC-MS (ESI$^+$)** RT= 2.49 min. m/z found: 581.42 $[M]^+$.

Supplementary References

1. Hall, M.P. et al. Engineered Luciferase Reporter from a Deep Sea Shrimp Utilizing a Novel Imidazopyrazinone Substrate. *ACS Chemical Biology* **7**, 1848-1857 (2012).
2. Liu, Z.Z. et al. Semiconductor quantum dots photosensitizing release of anticancer drug. *Chem. Commun.* **47**, 1482-1484 (2011).
3. Kramer, J.R. & Deming, T.J. Preparation of Multifunctional and Multireactive Polypeptides via Methionine Alkylation. *Biomacromolecules* **13**, 1719-1723 (2012).
4. Lou, K. & Lovell, J.F. A quenched binuclear ruthenium(II) dimer activated by another photosensitizer. *ChemComm*. **50**, 3231-3233 (2014).
5. Keppler, A. et al. A general method for the covalent labeling of fusion proteins with small molecules in vivo. *Nat Biotechnol* **21**, 86-89 (2003).
6. Lemercier, G., Gendreizig, S., Kindermann, M. & Johnsson, K. Inducing and sensing protein-protein interactions in living cells by selective cross-linking. *Angew Chem Int Ed Engl* **46**, 4281-4284 (2007).
7. Yang, Y., Zhang, T., Huang, W. & Shen, Z. Piperidine Nucleophilic Substitution Without Solvent: An Efficient Synthesis of Raloxifene. *Synthetic Communications* **44**, 3271-3276 (2014).
8. Winkler, D.E. & Whetstone, R.R. Some Observations on the Pechmann Reaction1. *The Journal of Organic Chemistry* **26**, 784-787 (1961).
9. Kralovec, J. et al. Synthesis of methotrexate-antibody conjugates by regiospecific coupling and assessment of drug and antitumor activities. *Journal of Medicinal Chemistry* **32**, 2426-2431 (1989).
10. Francis, C.L. et al. Total Synthesis of Methotrexate-γ-TRIS-Fatty Acid Conjugates. *Australian Journal of Chemistry* **55**, 635-645 (2002).
11. Griss, R. et al. Bioluminescent sensor proteins for point-of-care therapeutic drug monitoring. *Nat Chem Biol* **10**, 598-603 (2014).
12. Lakowicz, J.R. Principles of Fluorescence Spectroscopy, Edn. 3rd. (Springer, 2006).
13. Suzuki, K. et al. Five colour variants of bright luminescent protein for real-time multicolour bioimaging. *Nat Commun* **7**, 13718/DOI: 10.1038/ncomms13718 (2016).
14. UV-Vis-IR Spectral Software 1.2, Fluortools, www.fluortools.com
15. Chouikhi, D. et al. Expanding the Scope of PNA-Encoded Synthesis (PES): Mtt-Protected PNA Fully Orthogonal to Fmoc Chemistry and a Broad Array of Robust Diversity-Generating Reactions. *Chem. Eur. J.* **18**, 12698-12704 (2012).
16. Gorska, K., Manicardi, A., Barluenga, S. & Winssinger, N. DNA-templated release of functional molecules with an azide-reduction-triggered immolative linker. *Chemical Communications* **47**, 4364-4366 (2011).
17. Liu, N. et al. Direct and two-step bioorthogonal probes for Bruton's tyrosine kinase based on ibrutinib: a comparative study. *Organic & Biomolecular Chemistry* **13**, 5147-5157 (2015).
18. WO2016/079693 A Process For The Preparation Of Ibrutinib.
19. Machleidt, T. et al. NanoBRET-A Novel BRET Platform for the Analysis of Protein-Protein Interactions. *ACS Chem Biol* **10**, 1797-1804 (2015).
20. Tietze, L.F. et al. Asymmetric synthesis and biological evaluation of glycosidic prodrugs for a selective cancer therapy. *ChemMedChem* **3**, 1946-1955 (2008).
